# Optimal cerebrovascular reactivity thresholds for the determination of individualized intracranial pressure thresholds in traumatic brain injury: a CAHR-TBI cohort study

**DOI:** 10.1186/s13054-025-05619-w

**Published:** 2025-10-06

**Authors:** Kevin Y. Stein, Donald Griesdale, Mypinder Sekhon, Francis Bernard, Clare Gallagher, Eric P. Thelin, Rahul Raj, Marcel Aries, Logan Froese, Andreas H. Kramer, Frederick A. Zeiler

**Affiliations:** 1 Department of Biomedical Engineering, Price Faculty of Engineering, University of Manitoba, Winnipeg, MB Canada; 2 Rady Faculty of Health Sciences, Max Rady College of Medicine, University of Manitoba, Winnipeg, MB Canada; 3 Department of Anesthesiology, Pharmacology, and Therapeutics, University of British Columbia, Vancouver, BC Canada; 4 Division of Critical Care, Department of Medicine, University of British Columbia, Vancouver, BC Canada; 5 Section of Critical Care, Department of Medicine, University of Montreal, Montreal, QC Canada; 6 Section of Neurosurgery, University of Calgary, Calgary, AB Canada; 7 Department of Clinical Neurosciences, University of Calgary, Calgary, AB Canada; 8 Hotchkiss Brain Institute, University of Calgary, Calgary, AB Canada; 9 Medical Unit Neurology, Karolinska University Hospital, Stockholm, Sweden; 10 Department of Clinical Neuroscience, Karolinska Institutet, Stockholm, Sweden; 11 Department of Neurosurgery, University of Helsinki and Helsinki University Hospital, Helsinki, Finland; 12 Department of Intensive Care, School of Mental Health and Neurosciences, Maastricht University Medical Center+, University Maastricht, Maastricht, Netherlands; 13 Department of Critical Care Medicine, University of Calgary, Calgary, AB Canada; 14 Section of Neurosurgery, Department of Surgery, Rady Faculty of Health Sciences, University of Manitoba, Winnipeg, MB Canada; 15 Pan Am Clinic Foundation, Winnipeg, MB Canada

**Keywords:** Personalized physiologic targets, Intracranial pressure, Individualized intracranial pressure thresholds, Cerebrovascular reactivity, Critical thresholds, Traumatic brain injury

## Abstract

It has been demonstrated that patient-specific intracranial pressure (ICP) thresholds are possible to derive using the function intersectionality between ICP and cerebrovascular reactivity (CVR). Such individualized ICP (iICP) thresholds represent a potential personalized medicine approach to neurocritical care management. However, it is currently unknown how various CVR thresholds compare in regard to deriving iICP. Here we attempt to identify the CVR thresholds that are best suited for iICP derivation. Leveraging 365 patient data sets from the CAnadian High-Resolution TBI (CAHR-TBI) Research Collaborative, iICP was derived using three ICP-based CVR indices: the pressure reactivity index (PRx); the pulse amplitude index (PAx); and the RAC index, and thresholds ranging from − 1 to + 1, in 0.05 increments. Patients were dichotomized based on 6-month outcome scores into Alive vs. Dead and Favorable vs. Unfavorable outcome. 2 × 2 tables were created for each threshold, grouping patients by outcome and whether their mean ICP was greater or less than their calculated iICP. Chi-squares were calculated for each table and subsequently plotted. The thresholds that produced the largest Chi-square values were identified as those able to derive the iICP with the greatest ability to predict outcomes. Next, Spearman rank correlation testing was used to evaluate associations between iICP, for each threshold, and measures of cerebral physiologic insult burden. With consideration of yield data, ability to predict outcome, and association with cerebral physiologic insult burden, a threshold of + 0.05 was identified for PRx. No optimal threshold could be identified for PAx or RAC.

**Supplementary Information:**

The online version contains supplementary material available at 10.1186/s13054-025-05619-w.

## Background

The devastating outcomes associated with acute biomechanical trauma to the brain, commonly known as traumatic brain injury (TBI), stem from both primary and secondary injury processes. Primary brain injury refers to the immediate structural damage inflicted on the brain at the time of the incident, while secondary brain injury refers to the ongoing damage caused by disruptions in cerebral physiology following the initial injury [1]. Since primary brain injury is relatively irreversible, moderate-to-severe TBI management is primarily focused on mitigating secondary injury processes, particularly intracranial hypertension and cerebral ischemia [2]. In order to prevent these two highly detrimental processes, current management guidelines recommend therapeutically maintaining intracranial pressure (ICP) below a threshold of 20 or 22 mmHg and cerebral perfusion pressure (CPP) within a target range of 60–70 mmHg [3, 4].

Over the past decades, numerous therapeutic advancements have significantly improved our ability to achieve these clinical targets. Despite this, however, there has been little to no improvement in the poor morbidity and mortality rates associated with moderate-to-severe TBI [1, 5, 6]. A possible explanation for this shortcoming is the “one size fits all” approach of the current management paradigm, which fails to consider individual phenotype and the dynamic nature of cerebral physiology. A growing body of literature is demonstrating that significant heterogeneity in injury-response exists within the population [7–12]. Additionally, it has been shown that a substantial amount of cerebral physiologic insult burden is unresponsive to current guideline-based management strategies and that treatment response can vary drastically between individual patients [12–16]. This exposes the significant limitations of the current management approach and calls for the development of personalized medicine methods that can be tailored to the individual patient.

Patient-specific ICP thresholds have emerged as a promising personalized medicine concept, drawing from the same fundamental principles underlying cerebral perfusion pressure optimum (CPPopt). These individualized ICP (iICP) thresholds were first introduced in 2014 by Lazaridis and colleagues, when the authors plotted ICP against the pressure reactivity index (PRx; correlation between ICP and mean arterial pressure [MAP]) and manually identified the ICP value past which PRx became persistently deranged (PRx > + 0.20) [17]. They found that iICP was identifiable in approximately 68% of patients and demonstrated a greater ability to predict long-term outcomes compared to guideline-based ICP thresholds. Six years later, Zeiler and colleagues expanded on this work by developing a semi-autonomous algorithm with 83.2% accuracy [18]. Using a multicentered cohort, the authors were able to identify an iICP in 65.3% of patients and found that mean hourly dose of ICP above iICP thresholds was a stronger predictor of mortality than dose above the conventional thresholds of 20 or 22 mmHg.

Despite the promising findings of these prior works, both studies relied, at least in part, on manual inspection of plots to identify iICP, thereby hindering any future development of a continuous iICP derivation algorithm that can be implemented at the patient bedside. Moreover, both studies exclusively used PRx and a threshold of + 0.20 to derive iICP, which raises the question of whether alternative cerebrovascular reactivity indices and thresholds might be more effective for deriving iICP. This is especially concerning given that indices based on the pulse amplitude of ICP (AMP), such as the pulse amplitude index (PAx – correlation between AMP and MAP) and the RAC index (the correlation (R) between AMP (A) and CPP (C)), have been suggested to be more strongly associated with long-term outcomes than PRx, in certain subgroup populations [19–21].

In response to these shortcomings, the MAIN-HUB lab (located in Winnipeg, Canada) developed an autonomous algorithm that is able to identify iICP with high accuracy, > 99% in the tested dataset [22], and without the need for manual inspection. Using this algorithm, a validation study was conducted where the feasibility of deriving iICP using PAx and RAC was demonstrated [22]. Additionally, PRx-, PAx-, and RAC-based iICP were compared for their ability to predict long-term outcomes, with results suggesting that PRx may provide the greatest utility in this regard. However, only a few thresholds were analyzed in this study. Without an in-depth comparison of these indices, using a comprehensive range of thresholds, it is not possible to make any conclusive statements on which index is best suited for deriving iICP. Furthermore, it remains unknown which thresholds provide the greatest utility in identifying iICP. Therefore, in this study, we perform a comprehensive thresholding analysis to identify the ideal PRx, PAx, and RAC thresholds for deriving iICP based on yield data, ability to predict outcome, and association with measures of cerebral physiologic insult burden.

## Methods

### Study design

We conducted a retrospective multicentered thresholding analysis leveraging existing archived data from the Canadian High Resolution-TBI (CAHR-TBI) Research Collaborative [23]. This ongoing multicenter research collaborative collects high-resolution physiologic data from all adult (≥ 18 years) moderate-to-severe TBI patients who are admitted for invasive physiologic monitoring to the intensive care unit (ICU) of one of four university-affiliated hospitals: Foothills Medical Centre (University of Calgary), Health Sciences Centre Winnipeg (University of Manitoba; Shared Health Manitoba), Maastricht University Medical Center+ (University of Maastricht), and Vancouver General Hospital (University of British Columbia). Demographic information, admission characteristics, imaging findings, and clinical outcome scores are also collected. All data is prospectively recorded in a fully de-identified fashion and archived into ongoing locally approved databases. Data collected during the following periods were available at the time of this analysis: 2011 to 2021 at the University of Calgary, 2019 to 2024 at the University of Manitoba, 2017 to 2022 at the University of Maastricht, and 2014 to 2019 at the University of British Columbia.

### Ethics

All aspects of data collection and anonymous data transfer between centers have been approved by each site’s local research ethics authority: the University of Calgary Conjoint Health Research Ethics Board (CHREB, H20-03759), the University of Manitoba Biomedical Research Ethics Board (BREB, H2017:181, H2017:188, H2020:118, B2023:001, H2024:217, and H2024:266), the University of Maastricht Medical Ethics Committee (16-4-243), and the University of British Columbia Clinical Research Ethics Board (CREB, REB20-0482). All methods were carried out in accordance with the relevant guidelines and regulations. Due to the retrospective nature of the study, and the fact that data collection was performed in an entirely anonymized manner, the University of Manitoba Biomedical Research Ethics Board waived the need to obtain informed consent.

### Patient population

All patients included in the CAHR-TBI Research Collaborative sustained moderate-to-severe TBI, defined as having an admission Glasgow Coma Scale (GCS) of less than 13. Patients received standard care as per the Brain Trauma Foundation (BTF) guidelines, which included invasive monitoring of ICP and arterial blood pressure (ABP), as well as maintenance of ICP below 20 or 22 mmHg and CPP above 60 mmHg [3, 4]. In accordance with local practice, elevated CPP was generally not treated. Additionally, neither cerebrovascular reactivity nor near-infrared spectroscopy (NIRS)-based regional cerebral oxygen saturation (rSO₂) monitoring were considered in clinical decision making. Physiologic data recording was initiated following ICU admission and placement of invasive monitoring devices, preferably within 24 h of injury, and proceeded until discontinuation of invasive monitoring by the treating physician. Post-discharge, patients had their long-term outcomes assessed at a 6-month follow-up appointment using the Glasgow Outcome Scale (GOS).

### Physiologic data collection

Continuous ICP monitoring was carried out using an intraparenchymal pressure probe (Codman ICP MicroSensor, Codman & Shurtlef Inc., Raynham, MA, USA; NEUROVENT-TEMP, RAUMEDIC, Helmbrechts, Germany; Camino ICP Monitor, Natus, Middleton, WI, USA), placed in the frontal lobe, or an external ventricular drain (EVD; Medtronic, Minneapolis, MN). ABP was monitored using a radial or femoral line connected to a pressure transducer (Baxter Healthcare Corp. CardioVascular Group, Irvine, CA, USA; Edwards, Irvine, CA, USA) that was zeroed at the level of the tragus [24, 25]. In select patients, rSO_2_ and/or brain tissue oxygenation (PbtO_2_) were also monitored. In such cases a NIRS regional oximeter (INVOS 5100 C or 7100, Covidien-Medtronic, Minneapolis, MN) and intra-parenchymal brain tissue oxygenation probe (Licox Brain Tissue Oxygen Monitoring System; Integra LifeSciences Corp., Plainsboro, New Jersey), placed in the frontal lobe, were used. All high-frequency, full waveform physiologic data was recorded from bedside ICU monitors in time-series at a frequency of at least 100 Hz using Intensive Care Monitoring “Plus” software (ICM +) (Cambridge Enterprise Ltd, Cambridge, UK, http://icmplus.neurosurg.cam.ac.uk) via direct digital data transfer or analog-to-digital conversion (DT9804/DT9826, Data Translations, Marlboro, MA, USA).

### Signal processing

Post-acquisition signal processing was performed using ICM + software. Signal artifact clearing was performed by the data collection team prior to the conception of this study’s objective or methodology. Artifact removal was performed using both manual and automated techniques. This included omitting data segments with implausible values, which were defined as values below 0 mmHg or above 100 mmHg, for ICP, or 300 mmHg, for ABP. Data segments that lacked waveform morphology (i.e. static signals) were also removed. Such segments represent various non-physiologic events, such as radial line flushing, monitor disconnection, and patient movement. For datasets where ICP was monitored using an EVD, drain opening artifacts were cleared manually.

After thorough clearing of artifacts, Fourier analysis of the fundamental amplitude of the ICP pulse waveform was performed on sequential 10-second intervals to derive AMP [26, 27]. A 10-second non-overlapping moving average filter was then applied to down-sample ICP and ABP (producing MAP) in order to concentrate on the frequency range associated with cerebral vasomotion and limit the influence of the respiratory cycle [5, 28, 29]. CPP was calculated using the following formula: CPP = MAP – ICP.

To assess cerebrovascular reactivity, three ICP-based indices (PRx, PAx, and RAC) were derived. PRx was calculated as the minute-by-minute updating Pearson correlation between 300-second windows of ICP and MAP [30–32], while PAx and RAC were calculated similarly using AMP and MAP, and AMP and CPP, respectively [19, 27]. To evaluate cerebral compliance, the compensatory reserve index (RAP; correlation (R) between AMP (A) and ICP (P)) was also derived [27, 33]. Lastly, following common practice in cerebral physiologic research [31, 34, 35], including all existing works on iICP [17, 18, 22], all data was down-sampled to minute-by-minute resolution for computational efficiency and output as a comma-separated values file for each patient.

### iICP derivation

**Fig. 1:**
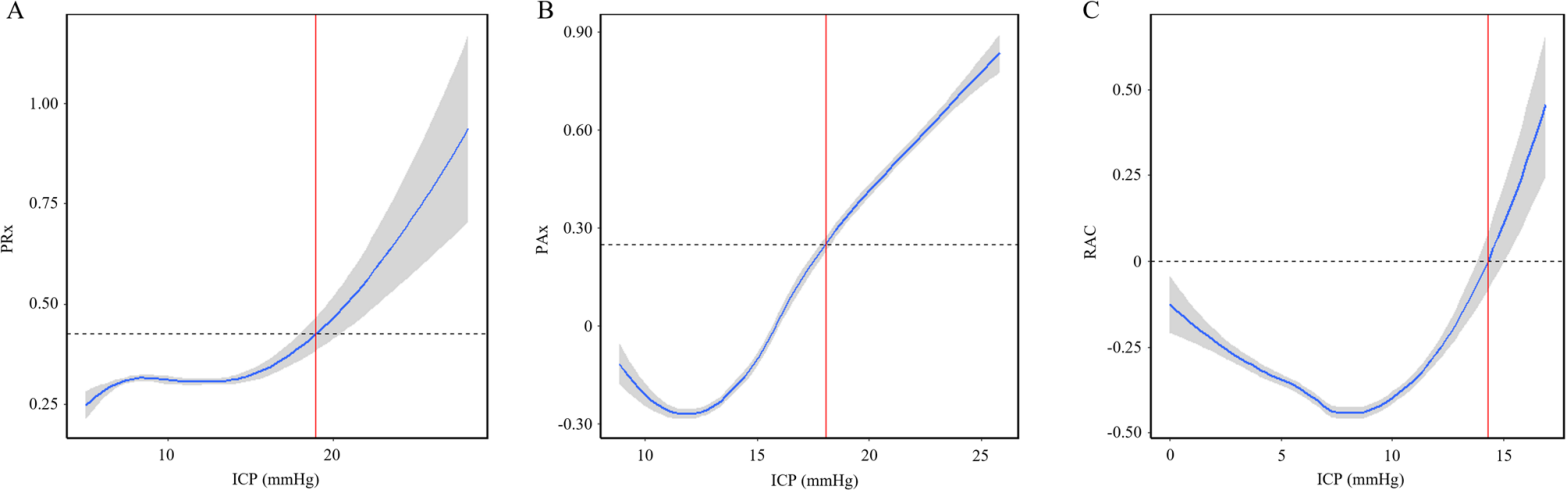
Example of iICP identification using LOESS curves of the ICP vs. cerebrovascular reactivity relationship iICP derivation using: (**A**) PRx > 0.35, (**B**) PAx > 0.25, (**C**) RAC > 0. These thresholds were selected based on a subset of values reported in the current literature on critical CVR thresholds for outcome prediction but are in no way universally accepted. Dashed line indicates the cerebrovascular reactivity threshold used for iICP derivation. Vertical red line indicates the identified iICP value. Grey regions surrounding each curve represent the 95% confidence intervals. *AMP = pulse amplitude of ICP*,* CPP = cerebral perfusion pressure*,* CVR = cerebrovascular reactivity*,* iICP = individualized intracranial pressure threshold*,* LOESS = locally weighted scatterplot smoothing*,* PAx = pulse amplitude index*,* PRx = pressure reactivity index*,* RAC = correlation (R) between slow-waves of AMP (A) and CPP (C)*

The MAIN-HUB lab recently developed an autonomous algorithm for the derivation of iICP using R Statistical Computing software (R Core Team (2020). R: A language and environment for statistical computing. R Foundation for Statistical Computing, Vienna, Austria. URL https://www.R-project.org/) [22]. In short, the algorithm does the following. First, a locally weighted scatterplot smoothing (LOESS) model is fit to the patient’s dataset (entire recording period), between ICP and PRx (any cerebrovascular reactivity index can be used in place of PRx). Next, PRx is estimated for every 0.01 mmHg increment of the dataset’s ICP range (minimum to maximum ICP). A 95% confidence interval is also calculated for each estimate using bootstrapping methods. The modeled data is then plotted, and the first intersection point between the LOESS curve and the set PRx threshold (any value between − 1 and + 1 can be chosen) where the curve remains above threshold for at least 10 mmHg following intersection (or to the end of the data range when less than a 10 mmHg range exists following intersection) is identified as the iICP threshold. This requirement to stay above threshold following intersection functions to prevent identifying points where the curve only transiently crosses threshold. When tested on the Winnipeg datasets, the algorithm was found to be more than 99% accurate upon manual inspection [22]. Example LOESS curves generated by this algorithm can be found in Fig. 1.

For the purposes of this study, the custom iICP derivation algorithm from the MAIN-HUB lab was run using three ICP-based cerebrovascular reactivity indices (PRx, PAx, and RAC) and thresholds ranging from − 1 to + 1 in 0.05 increments (41 thresholds per index, 123 index-threshold combinations). A filtered version of iICP, designated as iICP.ci, was also created by including only iICP values that were associated with a 95% confidence interval of less than 0.2 a.u. in its LOESS plot.

### Statistical analysis

Statistical analysis was conducted in R statistical computing software using the following packages: *purrr*, *ggplot2*, and *gridExtra*. First, mean values of all physiologic variables, along with % time spent above/below literature-defined thresholds, were calculated for each patient. Additionally, mean hourly doses of ICP above guideline-based thresholds of 20 mmHg and 22 mmHg, as well as above each rendition of iICP, were calculated following the methodology laid out by Zeiler and colleagues [18]. Physiologic variables and demographic data were summarized as medians and IQRs or as raw counts, where applicable. Histograms were then generated for the index-threshold pairs to illustrate the distributions of iICP values they produced for the patient cohort. The iICP derivation yield of each index-threshold pair was also calculated.

Next, patients were dichotomized based on their 6-month GOS score into Alive (GOS > 1) versus Dead (GOS = 1) and Favorable (GOS > 3) versus Unfavorable (GOS ≤ 3). The iICPs derived using the various index-threshold pairs were then compared for outcome prediction ability using a sequential chi-square method, similar to that of previous studies investigating critical thresholds of cerebrovascular reactivity indices [31, 36–38]. Sequential 2 × 2 tables were constructed for each index-threshold pair, grouping patients by outcome (Alive vs. Dead or Favorable vs. Unfavorable) and by whether their mean ICP was greater than or less than their calculated iICP value. Pearson’s Chi-squares were then calculated for each 2 × 2 table and tabulated. Next, the resulting Chi-square values of the index-threshold pairs were plotted alongside their respective iICP derivation yields. Only pairs associated with an iICP yield greater than 15% had their Chi-square value tabulated and plotted, as those with smaller yields were likely to produce unreliable statistics. Index-threshold pairs with the largest statistically significant Chi-square values were identified as those able to produce iICP with the greatest ability to predict long-term outcomes.

Next, for each index-threshold pair, Spearman rank correlation coefficients were calculated between mean hourly dose above its derived iICP and various measures of cerebral physiologic insult burden: % time with CPP < 60mmHg, % time with CPP > 70mmHg, % time with PRx > 0.25, % time with PAx > 0.25, % time with RAC > 0, and % time with PbtO_2_ < 20mmHg. These measures were used as they reflect different aspects of cerebral physiologic insult burden and, thus, offer valuable insight into how effectively a given iICP approach may help minimize secondary brain injury. The correlation coefficients and p values of index-threshold pairs with iICP derivation yields greater than 15% were then tabulated. Chi-square analysis and Spearman rank correlation testing were repeated for iICP.ci to evaluate for differences when only iICP values associated with small confidence intervals were included. Finally, sub-group Chi-square analyses were performed using the following dichotomizations: age (< 40 vs. ≥ 40 years), sex (male vs. female), GCS-motor score (≤ 3 vs. > 3), and Marshall computerized tomography (CT) score (≤ 3 vs. > 3). For all statistical testing, the threshold for statistical significance was set to *p* < 0.05.

### Identifying ideal thresholds

Using the results of the iICP derivation yields, Chi-square analysis, and Spearman rank correlation testing, the ideal PRx, PAx, and RAC thresholds for iICP derivation were determined. Since a negative index value is generally acknowledged to represent a state of intact cerebrovascular reactivity, we decided to not consider any PRx or PAx thresholds below 0 as physiologically relevant for deriving iICP. For RAC, we chose a cutoff point of −0.20 instead, since recent literature suggests that RAC may have a more negative transition point (between intact and impaired reactivity) [32, 39]. This approach was employed to prevent the identification of thresholds that are highly unlikely to be physiologically meaningful, as a threshold within the “intact” range would not be expected, in theory, to effectively identify cerebrovascular physiological insult.

## Results

### Patient population

A total of 365 patients were included from the CAHR-TBI Research Collaborative (120 originating from the University of Calgary, 125 from the University of Manitoba, 51 from the University of Maastricht, and 69 from the University of British Columbia). ICP and ABP recordings were available for all included patients; however, PbtO_2_ and NIRS-based rSO_2_ data were only available for 106 and 137 patients, respectively. 31 patients did not have a 6-month GOS score recorded due to loss to follow-up. The median age of this cohort was 38 years (IQR: 24–55) and approximately 78% of patients were male. The median GCS of the cohort was 6 (IQR: 4–7), with 64% patients alive at 6-month follow-up and 50% being assessed as having a favorable outcome. The median duration of physiologic data recording was about 97 h (IQR: 51–173). A complete summary of demographics and cerebral physiology of this patient cohort can be found in Table 1. Histograms illustrating the distributions of iICP and iICP.ci values produced for the cohort by each threshold (only those associated with a yield greater than 15%) can be found in Supplemental Appendices A-C, for PRx, PAx, and RAC, respectively. Generally, with greater thresholds, fewer iICP values remained when filtered for those with small confidence intervals (iICP.ci).

**Table 1:** Patient cohort demographics and cerebral physiology summary

Variable		Median (IQR) or Raw numbers (%)
Number of patients		365
Age (years)		38 (24–55)
Sex	Male	283 (78%)
	Female	81 (22%)
	NA	1
Admission GCS		6 (4–7)
Admission GCS – Motor		4 (1–5)
Admission pupil response	Bilaterally reactive	237 (67%)
	Unilaterally unreactive	63 (18%)
	Bilaterally unreactive	55 (15%)
	NA	10
Marshall CT score		3 (2–5)
GOS		3 (1–5)
Alive vs. Dead	Alive (GOS > 1)	213 (64%)
	Dead (GOS = 1)	121 (36%)
	NA	31
Favorable vs. Unfavorable outcome	Favorable (GOS 4–5)	166 (50%)
	Unfavorable (GOS 1–3)	168 (50%)
	NA	31
Hypoxia episode	Yes	61 (28%)
	No	160 (72%)
	NA	144
Hypotension episode	Yes	34 (16%)
	No	185 (84%)
	NA	146
Recording duration (hours)		96.95 (51.02–173)
Mean MAP (mmHg)		87.26 (81.71–93.98)
Mean ICP (mmHg)		12.47 (8.46–16.04)
% Time ICP > 20 mmHg		5.069 (0.7931–19.1)
% Time ICP > 22 mmHg		2.521 (0.2874–11.63)
Mean CPP (mmHg)		74.64 (70.2–80.96)
% Time CPP < 60 mmHg		4.69 (1.324–9.843)
% Time CPP > 70 mmHg		67.87 (48.88–85.96)
Mean PRx		0.11 (0.00–0.23)
% Time PRx > 0		61.65 (49.21–74.59)
% Time PRx > 0.25		36.68 (24.86–50.84)
% Time PRx > 0.35		27.74 (17.51–39.62)
Mean PAx		−0.02 (−0.13–0.11)
% Time PAx > 0		47.38 (35.09–63.61)
% Time PAx > 0.20		26.55 (16.51–42.42)
% Time PAx > 0.25		22.1 (13.71–36.17)
Mean RAC		−0.28 (−0.45 – −0.10)
% Time RAC > 0		22.08 (11.56–37.97)
Mean RAP		0.67 (0.51–0.77)
% Time RAP > 0.40		82.3 (70.21–90.9)
rSO_2_ (%)		70.08 (64.15–75.6)
Mean COx		0.02 (−0.02–0.08)
% Time COx > 0.20		24.02 (17.81–31.22)
Mean COx_a		0.06 (0.01–0.11)
% Time COx_a > 0.20		28.84 (21.65–37.23)
Mean PbtO2 (mmHg)		22.84 (13.67–31.37)
% Time PbtO2 < 15 mmHg		8.32 (1.53–74.4)
% Time PbtO2 < 20 mmHg		11.48 (2.006–52.74)

*ABP = arterial blood pressure*,* AMP = pulse amplitude of ICP*,* COx = cerebral oxygenation index (correlation between rSO*_*2*_ *and CPP)*,* COx_a = cerebral oxygenation index (correlation between rSO2 and ABP)*,* CPP = cerebral perfusion pressure*,* CT = computed tomography*,* GCS = Glasgow Coma Scale*,* GOS = Glasgow Outcome Scale*,* ICP = intracranial pressure*,* IQR = interquartile range*,* MAP = mean arterial pressure*,* PAx = pulse amplitude index (correlation between AMP and MAP)*,* PbtO2 = brain tissue oxygen tension*,* PRx = pressure reactivity index (correlation between ICP and MAP)*,* RAC = correlation (R) between slow waves of AMP (A) and CPP (C)*,* RAP = compensatory reserve index (correlation between AMP and ICP)*,* rSO*_*2*_ *= regional cerebral oxygen saturation*

### Chi-square outcome analysis and derivation yields

**Fig. 2:**
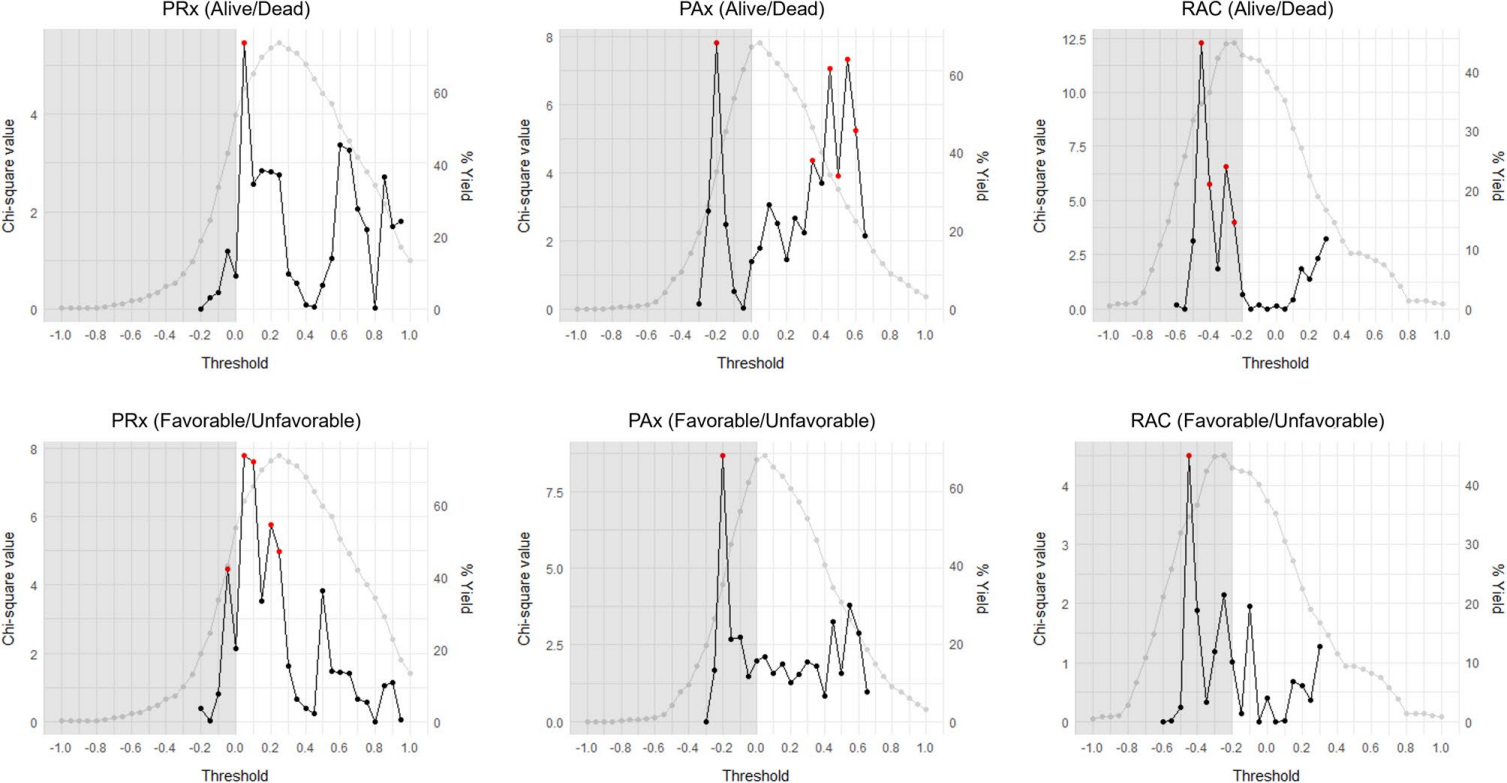
Chi-square outcome plots of iICP derived using various cerebrovascular reactivity thresholds.*AMP = pulse amplitude of ICP*,* CPP = cerebral perfusion pressure*,* ICP = intracranial pressure*,* iICP = individualized intracranial pressure thresholds*,* MAP = mean arterial pressure*,* PAx = pulse amplitude index (correlation between AMP and MAP)*,* PRx = pressure reactivity index (correlation between ICP and MAP)*,* RAC = correlation (R) between slow waves of AMP (A) and CPP (C)*.*The grey points illustrate the percent yields of each threshold in deriving iICP*.* The black and red points illustrate the chi-square values for predicting outcome (Alive vs Dead or Favorable vs Unfavorable) of each threshold that produced a percent yield greater than 15%. Red points indicate thresholds that reached statistical significance*,* p *<* 0.05*.* Greyed out area represents the range where cerebrovascular reactivity is generally considered to be intact*

The results of the Chi-square thresholding analysis for iICP can be found tabulated in Supplemental Appendices D-F (for PRx, PAx, and RAC, respectively) and plotted in Fig. 2. The results for iICP.ci can be found tabulated in Supplemental Appendices G-I (for PRx, PAx, and RAC, respectively) and plotted in Supplemental Appendix J. The 31 patients who did not have a 6-month GOS score recorded were excluded from this part of the analysis.

For PRx-based iICP, the threshold producing the greatest, and only statistically significant, Chi-square value for mortality prediction was + 0.05 (χ^2^ = 5.48). For favorable outcome prediction, five thresholds were able to reach statistical significance; however, + 0.05 (χ^2^ = 7.79) was able to produce the greatest Chi-square here as well. Additionally, the other four thresholds all fell within the close vicinity of + 0.05. The iICP derivation yield for this threshold was 61.37%. The largest yield was produced when using PRx > + 0.25 (73.97%); however, this threshold failed to produce a statistically significant Chi-square in this analysis. Testing for iICP.ci derivation generated similar results; however, was associated with slightly greater Chi-squares for + 0.05 in both mortality prediction (χ^2^ = 5.86) and favorable outcome prediction (χ^2^ = 8.10), as well as lower yields for all thresholds (+ 0.05 produced a yield of 54.79%).

For PAx-based iICP, the threshold producing the greatest Chi-square for mortality prediction was − 0.20 (χ^2^ = 7.82). However, it should be noted that this falls within the range of thresholds that is generally considered to represent intact cerebrovascular reactivity (−1 to ~ 0). Unlike with PRx, which produced a single distinct peak, we observed an additional cluster of statistically significant thresholds on the PAx plot, between + 0.35 and + 0.60. Although these thresholds produced Chi-square values (χ^2^ = 3.89–7.33) that were smaller than that of − 0.20, those of + 0.45 (χ^2^ = 7.06) and + 0.55 (χ^2^ = 7.33) were only marginally smaller. When testing for favorable outcome prediction, only − 0.20 (χ^2^ = 8.68) was able to produce a statistically significant Chi-square. The iICP yields for − 0.20, + 0.45, and + 0.55 were 35.07%, 34.25%, and 26.03%, respectively, which are considerably lower than the largest yield at 68.22%, associated with the threshold + 0.05. When testing iICP.ci derivation, only a single distinct peak was observed at −0.20 for both mortality prediction (χ^2^ = 10.19) and favorable outcome prediction (χ^2^ = 9.43). As with PRx, yields for all thresholds were again found to be smaller when deriving iICP.ci (29.86%, 12.88%, and 9.59% for − 0.20, + 0.45, and + 0.55, respectively).

For RAC, iICP derived using a threshold of −0.45 produced the greatest Chi-square (χ^2^ = 12.31) for predicting mortality. It should be noted that this threshold falls within the range of RAC thresholds that is generally considered to represent intact cerebrovascular reactivity (−1 to ~ −0.20). Three other thresholds also reached statistical significance; however, all of these fell in the relative proximity of − 0.45 and produced significantly smaller Chi-square values (χ^2^ = 3.99–6.56). For favorable outcome prediction, only − 0.45 produced a statistically significant Chi-square (χ^2^ = 4.51). The iICP yield for − 0.45 was 34.52%, just over 10% smaller than the largest yield of 44.93%, associated with the threshold − 0.25. Testing for iICP.ci derivation resulted in a nearly identical pattern, except Chi-squares were greater for − 0.45 in both mortality prediction (χ^2^ = 16.33) and favorable outcome prediction (χ^2^ = 7.43), and yields were lower for all thresholds (25.48% for − 0.45).

### Associations with cerebral physiologic insult burden metrics

The results of the Spearman rank correlation testing of PRx thresholds for iICP derivation can be found in Table 2. For % time with CPP < 60mmHg, the thresholds 0 (ρ = 0.22, *p* = 0.002) and + 0.35 (ρ = 0.22, *p* < 0.001) produced the strongest correlation coefficients; while + 0.05 produced a slightly weaker correlation (ρ = 0.17, *p* = 0.009). For % time with CPP > 70mmHg, 0 (ρ = −0.22, *p* = 0.002) and + 0.65 (ρ = −0.22, *p* = 0.004) had the strongest correlations, however, + 0.05 also resulted in a relatively strong correlation (ρ = −0.20, *p* = 0.002). For both % time with PAx > 0.25 and % time with RAC > 0, −0.20 produced the strongest correlations (ρ = 0.58, *p* < 0.001; ρ = 0.37, *p* < 0.001), while + 0.05 produced slightly more modest correlations (ρ = 0.49, *p* < 0.001; ρ = 0.30, *p* < 0.001). Finally, for % time with PbtO_2_ < 20mmHg, none of the thresholds were able to produce statistically significant correlations. The results for iICP.ci can be found in Supplemental Appendix K.

**Table 2:**
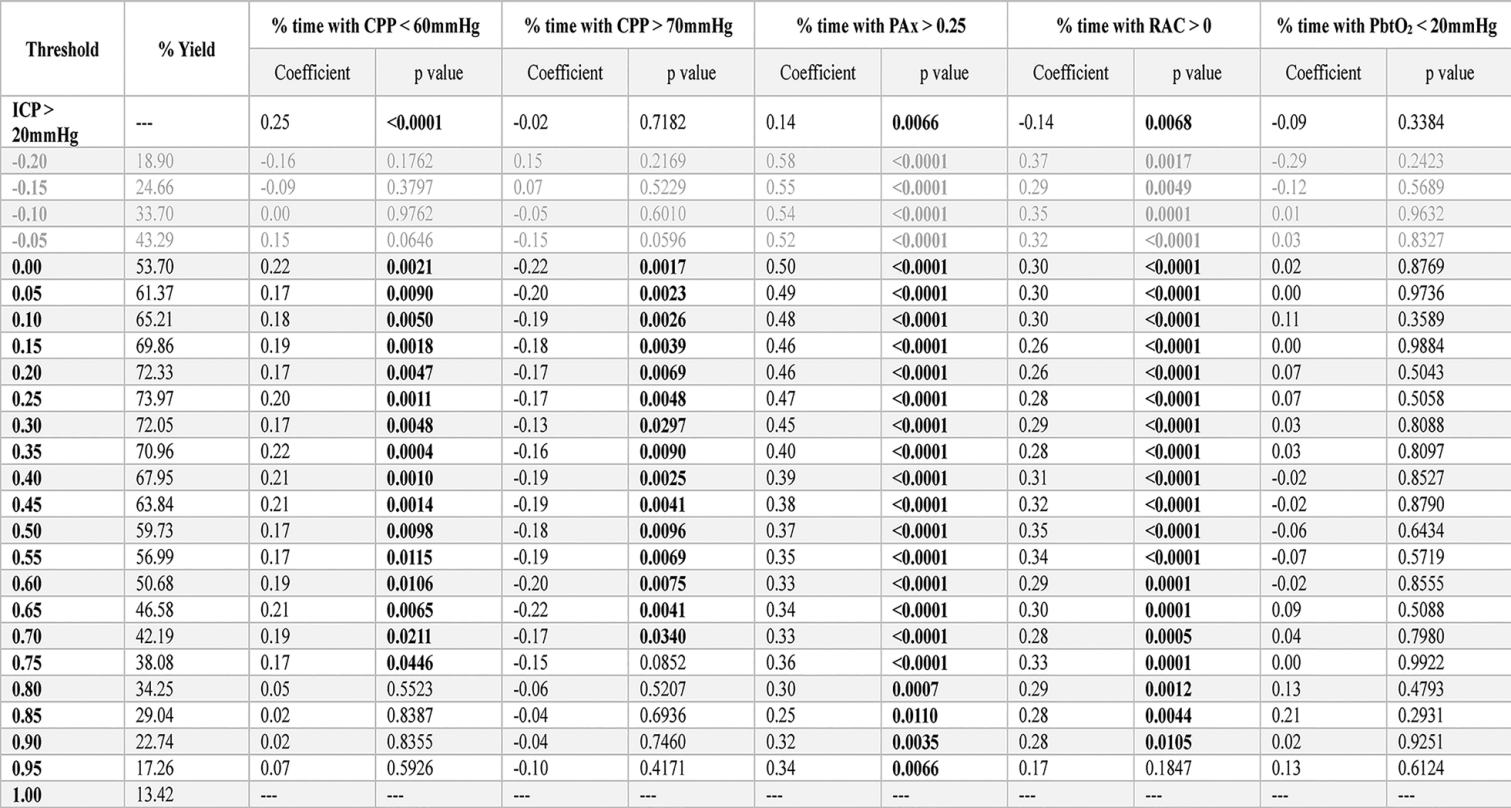
Spearman rank correlation analysis between iICP derived using various PRx thresholds and measures of cerebral physiologic insult burden

Bolded p values are those reaching statistical significance, *p* < *0.05*. *Grey rows represent the PRx range where cerebrovascular reactivity is generally considered to be intact*.* Thresholds in the intact range that did not produce the minimum 15% iICP yield are not displayed*. *AMP* = *pulse amplitude of ICP*, *CPP* = *cerebral perfusion pressure*, *ICP* = *intracranial pressure*, *iICP* = *individualized intracranial pressure thresholds*, *MAP* = *mean arterial pressure*, *PAx* = *pulse amplitude index (correlation between AMP and MAP)*, *PbtO2* = *brain tissue oxygen tension*, *PRx* = *pressure reactivity index (correlation between ICP and MAP)*, *RAC* = *correlation (R) between slow waves of AMP (A) and CPP (C)*

For PAx-based iICP, the results of the Spearman rank correlation testing can be found in Table 3. The threshold + 0.55 was able to produce the strongest correlations for both % time with CPP < 60mmHg (ρ = 0.29, *p* = 0.005) and % time with CPP > 70mmHg (ρ = −0.22, *p* = 0.03). For both of these insult burden measures, − 0.20 failed to produce statistically significant correlations. For % time with PRx > 0.25, + 0.05 produced the strongest correlation (ρ = 0.48, *p* < 0.001), beating out both − 0.20 (ρ = 0.33, *p* < 0.001) and + 0.55 (ρ = 0.34, *p* < 0.001). For % time with RAC > 0, + 0.40 produced the strongest correlation (ρ = 0.39, *p* < 0.001), with + 0.55 producing an, only marginally, weaker correlation (ρ = 0.36, *p* < 0.001). No thresholds were able to derive iICP with a statistically significant correlation with % time with PbtO_2_ < 20mmHg. The results for iICP.ci can be found in Supplemental Appendix L.

**Table 3:**
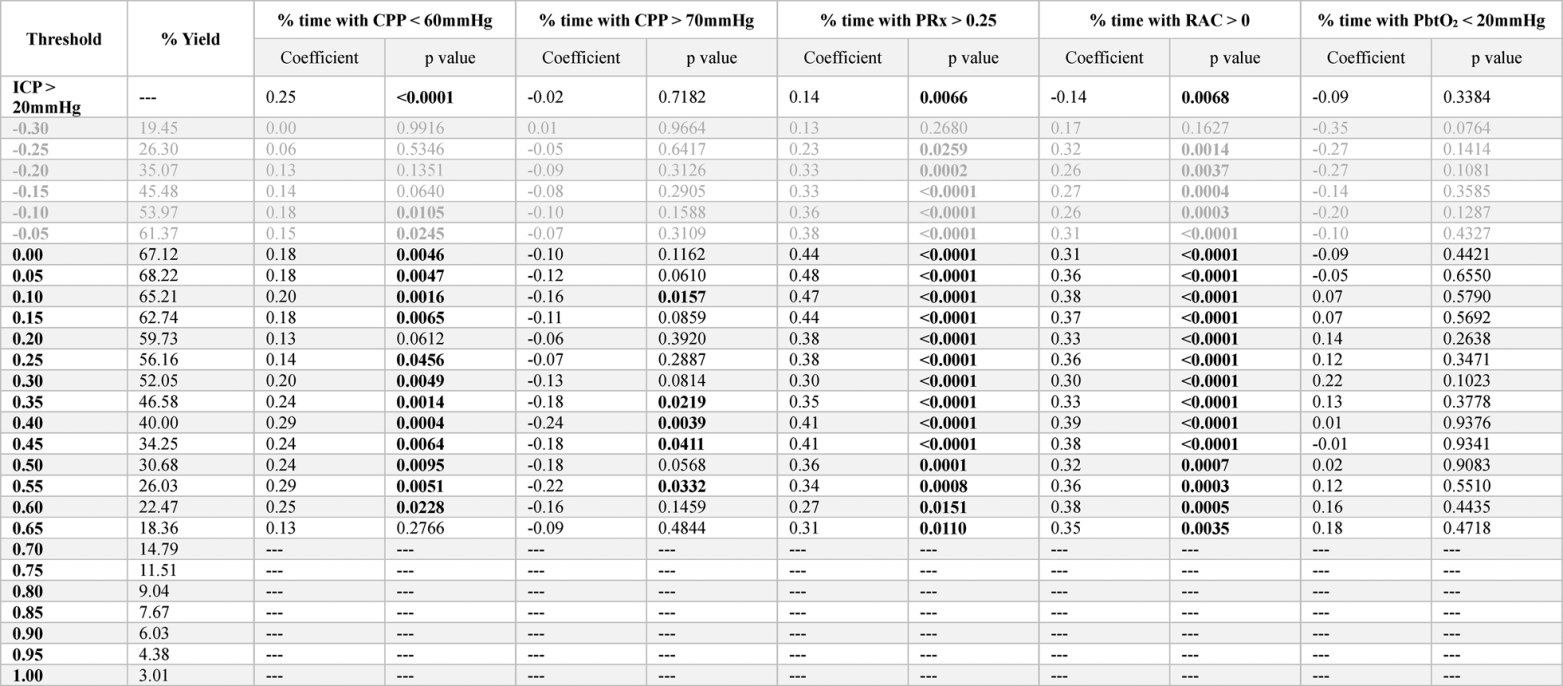
Spearman rank correlation analysis between iICP derived using various PAx thresholds and measures of cerebral physiologic insult burden

Bolded p values are those reaching statistical significance, *p* < *0.05*. *Grey rows represent the PAx range where cerebrovascular reactivity is generally considered to be intact*. *Thresholds in the intact range that did not produce the minimum 15% iICP yield are not displayed*. *AMP* = *pulse amplitude of* *ICP*, *CPP *= *cerebral perfusion pressure*, *ICP* = *intracranial pressure*, *iICP* = *individualized intracranial pressure thresholds*, *MAP* = *mean arterial pressure*, *PAx* = *pulse amplitude index (correlation between AMP and MAP)*, *PbtO2* = *brain tissue oxygen tension*, *PRx* = *pressure reactivity index (correlation between ICP and MAP)*, *RAC* = *correlation (R) between slow waves of AMP (A) and CPP (C)*

For iICP derived using RAC, the results of the Spearman rank correlation testing can be found in Table 4. For % time with CPP < 60mmHg, % time with CPP > 70mmHg, and % time with PRx > 0.25, the threshold that was able to produce the strongest correlations was + 0.30 (ρ = 0.51, *p* < 0.001; ρ = −0.43, *p* < 0.001; ρ = 0.48, *p* < 0.001; respectively); however, it should be noted that the iICP derivation yield for this threshold was only a mere 16.71%. From these three cerebral physiologic insult burden metrics, the threshold of − 0.45, identified during the Chi-square analysis, was only able to produce a statistically significant correlation with % time with CPP < 60mmHg (ρ = 0.29, *p* = 0.001). For % time with PAx > 0.25, + 0.20 was able to produce the strongest correlation (ρ = 0.62, *p* < 0.001). Lastly, for % time with PbtO_2_ < 20mmHg, −0.25 was the only threshold able to produce a statistically significant correlation (ρ = −0.33, *p* = 0.015). The results for iICP.ci can be found in Supplemental Appendix M.

**Table 4:**
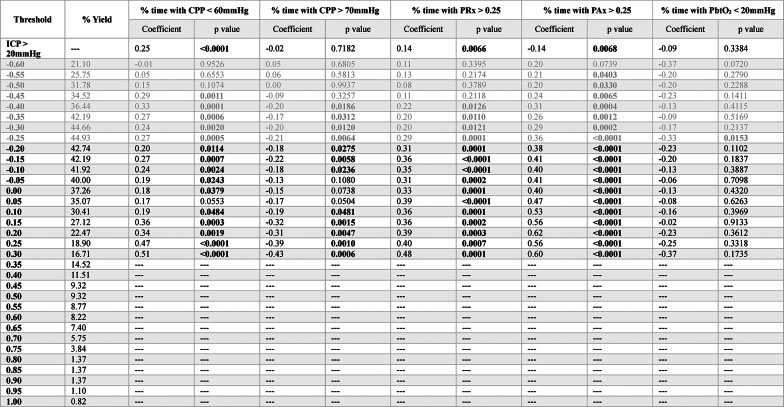
Spearman rank correlation analysis between iICP derived using various RAC thresholds and measures of cerebral physiologic insult burden

Bolded p values are those reaching statistical significance, *p* < *0.05*. *Grey rows represent the RAC range where cerebrovascular reactivity is generally considered to be intact*. *Thresholds in the intact range that did not produce the minimum 15% iICP yield are not displayed*. *AMP* = *pulse amplitude of* *ICP*, *CPP *= *cerebral perfusion pressure*, *ICP* = *intracranial pressure*, *iICP* = *individualized intracranial pressure thresholds*, *MAP* = *mean arterial pressure*, *PAx* = *pulse amplitude index (correlation between AMP and MAP)*, *PbtO2* = *brain tissue oxygen tension*, *PRx* = *pressure reactivity index (correlation between ICP and MAP)*, *RAC* = *correlation (R) between slow waves of AMP (A) and CPP (C)*

### Subgroup analysis

The Chi-square results when patients were dichotomized based on age into those who were < 40 years of age and those who were ≥ 40 years of age can be found in Supplemental Appendices N-P. The results when patients were dichotomized based on sex can be found in Supplemental Appendices Q-S. The GCS-motor dichotomization results can be found in Supplemental Appendices T-V. The results of the dichotomization based on Marshall CT score can be found in Supplemental Appendices W-Y. The threshold that performed best in the < 40 age and male cohort was the same as the threshold that performed best in the main Chi-square analysis (PRx > + 0.05). However, the ≥ 40 and female cohorts failed to produce any statistically significant results. The GCS-motor and Marshall CT score dichotomizations did not result in any meaningful results.

## Discussion

### Identifying critical thresholds

In this study, we performed a thorough thresholding analysis in hopes of identifying cerebrovascular reactivity thresholds that provide the most utility in deriving iICP. Beginning with PRx, a distinct threshold emerged during the Chi-square outcome analysis, + 0.05. This was the only threshold able to produce a statistically significant Chi-square for both mortality and favorable outcome prediction. Though it was unable to produce the strongest Spearman rank correlations with the cerebral physiologic insult burden measures, it was able to produce relatively strong correlations, and no other threshold was able to consistently produce the strongest correlations either. Additionally, the iICP derivation yield for + 0.05 was relatively high at 61.37%, only about 12% less than the largest yield observed. Based on the above findings, we identified + 0.05 as the likely optimal threshold for PRx-based iICP derivation. However, it is worth noting, that patients spent a relatively limited amount of time with severely elevated ICP, as shown in Table 1. This may have influenced the identification of + 0.05 as the optimal PRx threshold, as most of the data points are likely concentrated at lower ICP values and, by extension (given the relationship between ICP and PRx), at lower PRx values. Thus, the statistical strength observed at this threshold may, at least in part, reflect the underlying distribution of the dataset rather than a definitive physiologic inflection point.

For PAx-based iICP, the Chi-square analysis suggested a threshold of −0.20, as it was the threshold that produced the greatest peak when plotted. Furthermore, it was the only threshold to produce statistically significant Chi-squares when filtering for iICP with tight confidence bands, iICP.ci. However, this threshold falls within the range generally considered to represent intact reactivity (−1 to ~ 0), and is therefore, as discussed in the methods, unlikely to be a physiologically relevant threshold for iICP derivation. Although another peak was observed at + 0.55 on the mortality prediction plot, with a Chi-square only marginally smaller than that of − 0.20, this threshold was unable to produce a statistically significant Chi-square for favorable outcome prediction. It also failed to achieve statistical significance when iICP was filtered for small confidence bands, iICP.ci. Upon Spearman rank correlation testing, − 0.20 did produce the strongest correlation with any of the insult burden measures and even failed to produce a statistically significant correlation with both % time with CPP < 60mmHg and % time with CPP > 70mmHg. On the other hand, + 0.55 was able to produce the strongest correlations for these two cerebral physiologic insult burden metrics, as well as produce relatively strong associations for both % time with PRx > 0.25 and % time with RAC > 0. This seems promising for the threshold; however, + 0.55 is quite high considering that the highest identified critical PAx threshold for outcome prediction has been + 0.25 [36, 38]. Additionally, the iICP derivation yield associated with + 0.55 was abysmal at a mere 26.03%. Therefore, there appears to be no clear ideal threshold for PAx-based iICP derivation.

The Chi-square analysis for RAC-based iICP seemed to suggest − 0.45 as the ideal threshold, since it was the threshold that produced the most distinct Chi-square peak for both mortality and favorable outcome prediction. However, upon Spearman rank correlation testing, the threshold was unable to produce statistically significant correlations with % time with CPP > 70mmHg, % time with PRx > 0.25, and % time with PbtO_2_ < 20mmHg. Furthermore, this threshold is well within the negative range of RAC values, and thus, unlikely represents a physiologically relevant threshold for deriving iICP. Although + 0.30 was able to produce the strongest correlations for % time with CPP < 60 mmHg, % time with CPP > 70mmHg, and % time with PRx > 0.25, it was unable to produce any statistically significant associations with outcome prediction upon Chi-square analysis. Additionally, + 0.30 was associated with an incredibly low derivation yield (16.71%), making it unviable for iICP derivation. Due to the lack of any meaningful results, no ideal threshold for RAC-based iICP could be identified. Moreover, the utility of RAC for deriving iICP remains highly unclear in general, given the complexity of interpreting this index (RAC provides insight into not only cerebrovascular reactivity but also cerebral compensatory reserve) [20]. Therefore, more work is needed to evaluate the role of RAC in deriving personalized physiologic metrics.

This study provides the first comprehensive comparison of cerebrovascular reactivity thresholds for deriving iICP. Unlike previous studies that derived iICP using an arbitrarily selected threshold, we provided an in-depth evaluation of how threshold choice influences the performance of iICP. This work will inform future works, specifically algorithm development, in threshold selection. However, it is important to clarify that the thresholds identified in this study do not represent cerebrovascular reactivity cut-off values that are themselves most predictive of outcome. Rather, they solely reflect the optimal thresholds for deriving iICP, defined as those that produced iICP values most strongly associated with clinical outcomes and multimodal cerebral physiology.

### Additional findings

Through this thresholding analysis, we made multiple additional interesting observations that deserve highlighting. Firstly, the findings of this study did not completely fall in line with the critical outcome prediction thresholds identified for the three cerebrovascular reactivity indices in recent literature. This is especially true for PAx and RAC, as we were unable to identify ideal thresholds for these. The current literature suggests critical thresholds in the ranges of 0 to + 0.25 and − 0.10 to + 0.05, for PAx and RAC, respectively [36, 38]. These critical thresholds were identified through association work between the cerebrovascular reactivity indices, as stand-alone parameters (not in deriving iICP), and long-term outcome using similar chi-square analyses. It would have been reasonable to expect that these critical thresholds would have been identified as the ideal thresholds for deriving iICP as well; however, this does not seem to be the case. For PRx, the literature has generally pointed towards a critical threshold within the range of + 0.25 to + 0.35 for mortality prediction [31, 36, 38]. However, there is some literature supporting a threshold of + 0.05. In one study by Sorrentino and colleagues, + 0.05 was identified as a critical threshold for favorable outcome prediction [31]. Furthermore, there is extensive pre-clinical animal literature suggesting that a PRx around 0 detects the lower limit of autoregulation [32, 40–42]. These studies provide some reassurance for the PRx threshold we identified here.

Second, PAx and RAC were associated with lower iICP derivation yields when compared to PRx. This mirrors recent findings from studies comparing the three indices for CPPopt derivation [43, 44]. One possible explanation for this is that, due to the highly controlled nature of ICP in the ICU setting, there may be too little variation in AMP to produce the needed variability in these indices to generate well fitted LOESS. This may result in identification of fewer iICP values (lower yield) or identification of inaccurate iICP values, both of which can blunt the ability of iICP to predict outcome. Therefore, it is likely that PRx represents the most practical cerebrovascular reactivity index for deriving iICP, since a low yields would significantly limit any clinical utility of iICP. However, we cannot make any conclusive statements on the underlying reasoning for this difference in yields. Additionally, it is important to note that these yields were produced using the entire recording periods of patients, and that a continuous multi-window weighted approach to iICP derivation, which would be necessary for clinical application, may produce different results. We, therefore, suggest that future iICP work not exclude these indices until further work has confirmed that they are inferior to PRx for iICP derivation.

Next, during Chi-square analysis, it was observed that favorable outcome prediction tended to produce greater Chi-squares than mortality prediction for PRx-based iICP, while producing smaller values than mortality prediction for RAC-based iICP. This suggests that PRx-based iICP is better at predicting favorable outcome than predicting mortality, while RAC-based iICP is better at predicting mortality than predicting favorable outcome. Also, for mortality prediction, RAC-based iICP produced greater Chi-squares than PAx-based iICP, which produced greater Chi-squares than PRx-based iICP. On the other hand, for favorable outcome prediction, PRx- and PAx-based iICP produced greater Chi-squares than RAC-based iICP. This suggests that RAC-based iICP may be best able to predict mortality, but the worst for predicting favorable outcome.

Regarding iICP.ci, it is interesting to see that at higher thresholds, more iICP values were filtered out than at the lower thresholds (see Supplemental Appendices A-C). This suggests that at higher thresholds, confidence in the accuracy of the identified iICP diminishes. iICP.ci also generally produced greater Chi-square values for outcome prediction, but lower yields, than compared to unfiltered iICP. This suggests that iICP based on confidence band size may result in greater ability to predict outcome, but at the expense of yield. Lastly, the subgroup analysis for age and sex was unable to identify any thresholds that were able to achieve significance for the ≥ 40 age group and female group. This may potentially suggest that various patient-specific factors can affect the utility of iICP, as well as the ideal cerebrovascular threshold for its derivation. However, this finding may be a result of differences in group sizes. Further work will be needed to investigate the role that patient demographics, injury severity, and treatment regimen has on iICP derivation.

### Limitations

Despite the important findings uncovered in this thresholding analysis, there are a couple noteworthy limitations that must be addressed. Firstly, the main limitation of this thresholding analysis is that we generated iICP using patients’ entire recording periods. Therefore, it remains unknown whether the ideal thresholds identified here would be applicable to a continuously derived iICP. Currently, no continuous iICP algorithm exists; however, once one is developed, a further thresholding analysis may be necessary to confirm the idealness of the identified thresholds for deriving iICP in real-time.

Another limitation of this study is that the chi-square analysis failed to produce smooth plots where the chi-square values gradually increase, peak at an “ideal” threshold, and then gradually decrease (similar to what is seen for the yield curves). Rather, the generated plots present an erratic curve with sudden spikes. This questions whether the thresholds found to produce the strongest chi-square values are physiologically significant and not just mere statistical anomalies. Future work using datasets from outside the CAHR-TBI collaborative, such as high-resolution datasets from the CENTER-TBI and TRACK-TBI studies [45, 46], will be needed to validate our findings.

Since cerebral hemodynamics exhibit significant variation throughout the different phases of post-TBI recovery, it is highly possible that the ideal thresholds for iICP derivation vary over the course of a patient’s time in the ICU [36, 47]. In this study we did not consider such variations in cerebral physiology, thus limiting our findings. Future studies should consider stratifying monitoring periods across patients’ times in the ICU to better understand how these variations in cerebral physiology may affect optimal iICP derivation.Next, though the patient cohort used in this study was quite large, only a portion of them had PbtO_2_ recordings available (*n* = 106). This may have underpowered any tests involving this physiologic variable and may possibly explain why only one index-threshold pair was able to generate an iICP that produced a statistically significant association with % time with PbtO_2_ < 20mmHg. Future work with larger PbtO_2_ datasets is warranted to better shed light on the association between iICP and this important cerebral physiologic parameter.

Lastly, this study is limited by the scope of data available in the CAHR-TBI database. For instance, the database does not document whether patients underwent decompressive craniectomy, a procedure that recent literature suggests may influence cerebrovascular reactivity [48]. The absence of this information restricts our ability to account for a potentially important confounding variable. Furthermore, the lack of contemporary CT scoring systems, such as the Rotterdam or Helsinki CT scores, and the use of GOS, rather than the more detailed extended version (GOSE), limit our analyses and may affect the precision and generalizability of our findings.

### Future directions

Unlike traditional static, population-based ICP thresholds, patient-specific ICP thresholds account for an individual’s dynamic cerebral autoregulatory status and may, in the future, enable treatment that is tailored to the individual’s specific physiologic needs. However, despite the promising preliminary findings regarding iICP, limited literature exists on the concept as of now. Additionally, the current state of the iICP concept is not conducive to clinical application. Firstly, the current algorithm requires a patient’s entire recording period, allowing only for the calculation of an “after the fact” threshold that is not usable to guide treatment. Moreover, it is only able to produce a singular threshold for a dataset and, therefore, does not take into account the dynamic nature of cerebral physiology over a patient’s time in the ICU. Another limitation of the current algorithm is that it fails to provide any assessment of curve fit characteristics. This prevents the clinical end-user from being able to gauge the quality of the output iICP value.

To circumvent these shortcomings, an algorithm that can continuously derive iICP in real-time is needed. Such an algorithm would require a sophisticated sliding multi-window weighted approach that, for each update interval (i.e. every minute), generates LOESS plots for various window lengths, scores plots based on a variety of factors (curve shape, confidence bands, data range, etc.), and calculates a weighted average to identify an iICP value. A similar strategy has been successfully leveraged in recent renditions of CPPopt [35, 49]. The algorithm should also present a summary of curve fit characteristics with each iICP calculation to allow for output quality assessment. Additionally, once a continuous algorithm is created, an assessment of whether the ideal CVR threshold for iICP derivation varies over different phases of the ICU stay (e.g., first 24 h vs. later periods) will be needed. This will provide valuable insight into how cerebrovascular reactivity impairment and iICP behavior evolve over time.

Following the development of such a continuous iICP derivation algorithm, thorough outcome analyses will be needed to provide preliminary insight into the prognostic utility of continuously derived iICP. Additionally, evaluation of the association between iICP and measures of cerebral physiologic insult burden will also be needed to shed light on the potential impact that iICP-directed care could have on minimizing secondary brain injury. However, to conclusively determine if iICP-directed care offers any real clinical benefit, a clinical trial would be needed.

Next, if iICP is to ever become implemented clinically, work will be needed to enable bedside implementation. This will require tailoring any continuously updating algorithm to the specific needs of the bedside environment and developing a user-interface that allows clinical end-users to efficiently use and adjust output values. Finally, while iICP represents a promising individualized approach to managing ICP, the integration of additional personalized cerebral physiologic metrics may further enhance the precision and utility of this tool. These include CPPopt, the mean arterial pressure optimum (MAPopt), and the bispectral index optimum (BISopt). In conjunction, these personalized metrics may help mitigate each other’s limitations, supporting a more comprehensive and effective strategy for bedside decision-making in neurocritical care.

## Conclusion

In this study, we used a multicentered dataset to identify optimal cerebrovascular reactivity thresholds for the derivation of iICP. Based on yield data, outcome prediction ability, and association with measures of cerebral physiologic insult burden, we were able to identify a threshold of + 0.05 for PRx-based iICP derivation; however, we were unable to identify an ideal threshold for either PAx- or RAC-based iICP. Despite the promising potential of iICP, the concept, in its current form, is significantly hindered by its use of patients’ entire recording periods, preventing any possibility of clinical application. Therefore, the development of a continuous iICP derivation algorithm, using a sliding multi-window weighted approach, is drastically needed. Once such an algorithm is developed, our findings require validation to ensure that they hold true for continuously derived iICP.

## Supplementary Information

Supplementary Material 1

## Data Availability

The datasets analyzed in this study are currently not publicly available as the Canadian and EU jurisdictions, including the research ethics boards and regional privacy bodies under which data was collected, do not allow for data sharing.

## Abbreviations

ABP: Arterial blood pressure
AMP: Pulse amplitude of ICP
BTF: Brain trauma foundation
CAHR-TBI: Canadian high resolution-TBI [Research collaborative]
COx: Cerebral oxygenation index (correlation between rSO_2_ and CPP)
COx_a: Cerebral oxygenation index (correlation between rSO_2_ and ABP)
CPP: Cerebral perfusion pressure
CPPopt: Cerebral perfusion pressure optimum
CT: Computed tomography
EVD: External ventricular drain
GCS: Glasgow coma scale
GOS: Glasgow outcome scale
ICM +: Intensive care monitoring “Plus” software
ICP: Intracranial pressure
ICU: Intensive care unit
iICP: individualized intracranial pressure
iICP.ci: iICP values filtered for those associated with a 95% confidence interval of less than 0.2 a.u
IQR: Interquartile range
LOESS: Locally weighted scatterplot smoothing
MAP: Mean arterial pressure
NIRS: Near infrared spectroscopy
PAx: Pulse amplitude index (correlation between AMP and MAP)
PbtO_2_: Brain tissue oxygenation
PRx: Pressure reactivity index (correlation between ICP and MAP)
RAC: Correlation (R) between AMP (A) and CPP (C)
RAP: Compensatory reserve index (correlation between AMP and ICP)
rSO₂: Regional cerebral oxygen saturation
TBI: Traumatic brain injury
