# Supplementary material for: Optimal cerebrovascular reactivity thresholds for the determination of individualized intracranial pressure thresholds in traumatic brain injury: a CAHR-TBI cohort study
Source: Crit Care. 2025 Oct 6;29:420. doi: 10.1186/s13054-025-05619-w (PMC12502327; doi:10.1186/s13054-025-05619-w)
Supplement: Supplementary file 1 — Supplementary Material 1 [file 13054_2025_5619_MOESM1_ESM.docx]

**Optimal Cerebrovascular Reactivity Thresholds for the Determination of Individualized Intracranial Pressure Thresholds in Traumatic Brain Injury: a CAHR-TBI Cohort Study**

Kevin Y. Stein,^1,2^ Donald Griesdale,^3^ Mypinder Sekhon,^3,4^ Francis Bernard,^5^ Clare Gallagher,^6-8^ Eric P. Thelin,^9,10^ Rahul Raj,^11^ Marcel Aries,^12^ Logan Froese,^9,10^ Andreas Kramer,^7,8,13^ Frederick A. Zeiler^1,10,14,15^

1. Department of Biomedical Engineering, Price Faculty of Engineering, University of Manitoba, Winnipeg, Manitoba, Canada
2. Max Rady College of Medicine, Rady Faculty of Health Sciences, University of Manitoba, Winnipeg, Manitoba, Canada
3. Department of Anesthesiology, Pharmacology, and Therapeutics, University of British Columbia, Vancouver, British Columbia, Canada
4. Division of Critical Care, Department of Medicine, University of British Columbia, Vancouver, British Columbia, Canada
5. Section of Critical Care, Department of Medicine, University of Montreal, Montreal, Quebec, Canada
6. Section of Neurosurgery, University of Calgary, Calgary, Alberta, Canada
7. Department of Clinical Neurosciences, University of Calgary, Calgary, Alberta, Canada
8. Hotchkiss Brain Institute, University of Calgary, Calgary, Alberta, Canada
9. Medical Unit Neurology, Karolinska University Hospital, Stockholm, Sweden
10. Department of Clinical Neuroscience, Karolinska Institutet, Stockholm, Sweden
11. Department of Neurosurgery, University of Helsinki and Helsinki University Hospital, Helsinki, Finland
12. Department of Intensive Care, Maastricht University Medical Center+ and School of Mental Health and Neurosciences, University Maastricht, Maastricht, Netherlands
13. Department of Critical Care Medicine, University of Calgary, Calgary, Alberta, Canada
14. Section of Neurosurgery, Department of Surgery, Rady Faculty of Health Sciences, University of Manitoba, Winnipeg, Manitoba, Canada
15. Pan Am Clinic Foundation, Winnipeg, Manitoba, Canada

**Supplemental Material**

**Tables of Contents:**

| **A** | Histograms of iICP derived using various PRx thresholds | 4 |
| --- | --- | --- |
| **B** | Histograms of iICP derived using various PAx thresholds | 5 |
| **C** | Histograms of iICP derived using various RAC thresholds | 6 |
| **D** | Chi-square outcome analysis of iICP derived using various PRx thresholds | 7 |
| **E** | Chi-square outcome analysis of iICP derived using various PAx thresholds | 8 |
| **F** | Chi-square outcome analysis of iICP derived using various RAC thresholds | 9 |
| **G** | Chi-square outcome analysis of iICP.ci derived using various PRx thresholds | 10 |
| **H** | Chi-square outcome analysis of iICP.ci derived using various PAx thresholds | 11 |
| **I** | Chi-square outcome analysis of iICP.ci derived using various RAC thresholds | 12 |
| **J** | Chi-square outcome plots of iICP.ci derived using various cerebrovascular reactivity thresholds | 13 |
| **K** | Spearman rank correlation analysis between iICP.ci derived using various PRx thresholds and measures of cerebral physiologic insult burden | 14 |
| **L** | Spearman rank correlation analysis between iICP.ci derived using various PAx thresholds and measures of cerebral physiologic insult burden | 15 |
| **M** | Spearman rank correlation analysis between iICP.ci derived using various RAC thresholds and measures of cerebral physiologic insult burden | 16 |
| **N** | Chi-square outcome analysis of iICP derived using various PRx thresholds dichotomized by age | 17 |
| **O** | Chi-square outcome analysis of iICP derived using various PAx thresholds dichotomized by age | 18 |
| **P** | Chi-square outcome analysis of iICP derived using various RAC thresholds dichotomized by age | 19 |
| **Q** | Chi-square outcome analysis of iICP derived using various PRx thresholds dichotomized by sex | 20 |
| **R** | Chi-square outcome analysis of iICP derived using various PAx thresholds dichotomized by age | 21 |
| **S** | Chi-square outcome analysis of iICP derived using various RAC thresholds dichotomized by age | 22 |
| **T** | Chi-square outcome analysis of iICP derived using various PRx thresholds dichotomized by sex | 23 |
| **U** | Chi-square outcome analysis of iICP derived using various PAx thresholds dichotomized by age | 24 |
| **V** | Chi-square outcome analysis of iICP derived using various RAC thresholds dichotomized by age | 25 |
| **W** | Chi-square outcome analysis of iICP derived using various PRx thresholds dichotomized by sex | 26 |
| **X** | Chi-square outcome analysis of iICP derived using various PAx thresholds dichotomized by age | 27 |
| **Y** | Chi-square outcome analysis of iICP derived using various RAC thresholds dichotomized by age | 28 |

Supplemental Appendix A. Histograms of iICP derived using various PRx thresholds


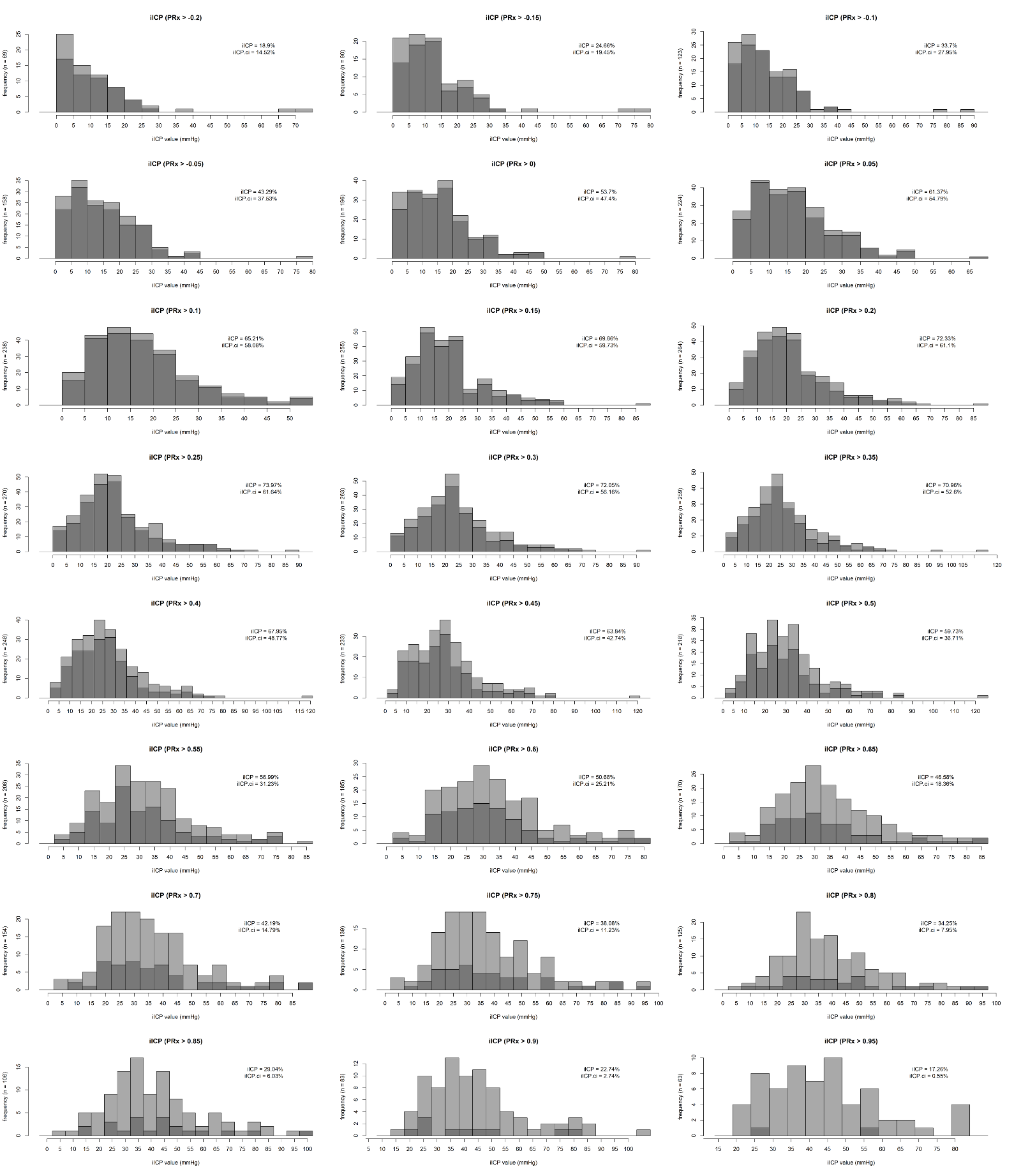


*ICP = intracranial pressure, iICP = individualized intracranial pressure thresholds, iICP.ci = individualized intracranial pressure thresholds with confidence intervals less than 0.2 a.u., MAP = mean arterial pressure, PRx = pressure reactivity index (correlation between ICP and MAP).*

*Histograms illustrate the distribution of derived iICP values for each threshold that produced a percent yield greater than 15%. The dark grey bars indicate the proportion of derived iICP values that remained after filtering for those with confidence intervals less than 0.2 a.u. (denoted iICP.ci). Percent yields of iICP and iICP.ci for each threshold are listed on their respective plots.*

Supplemental Appendix B. Histograms of iICP derived using various PAx thresholds
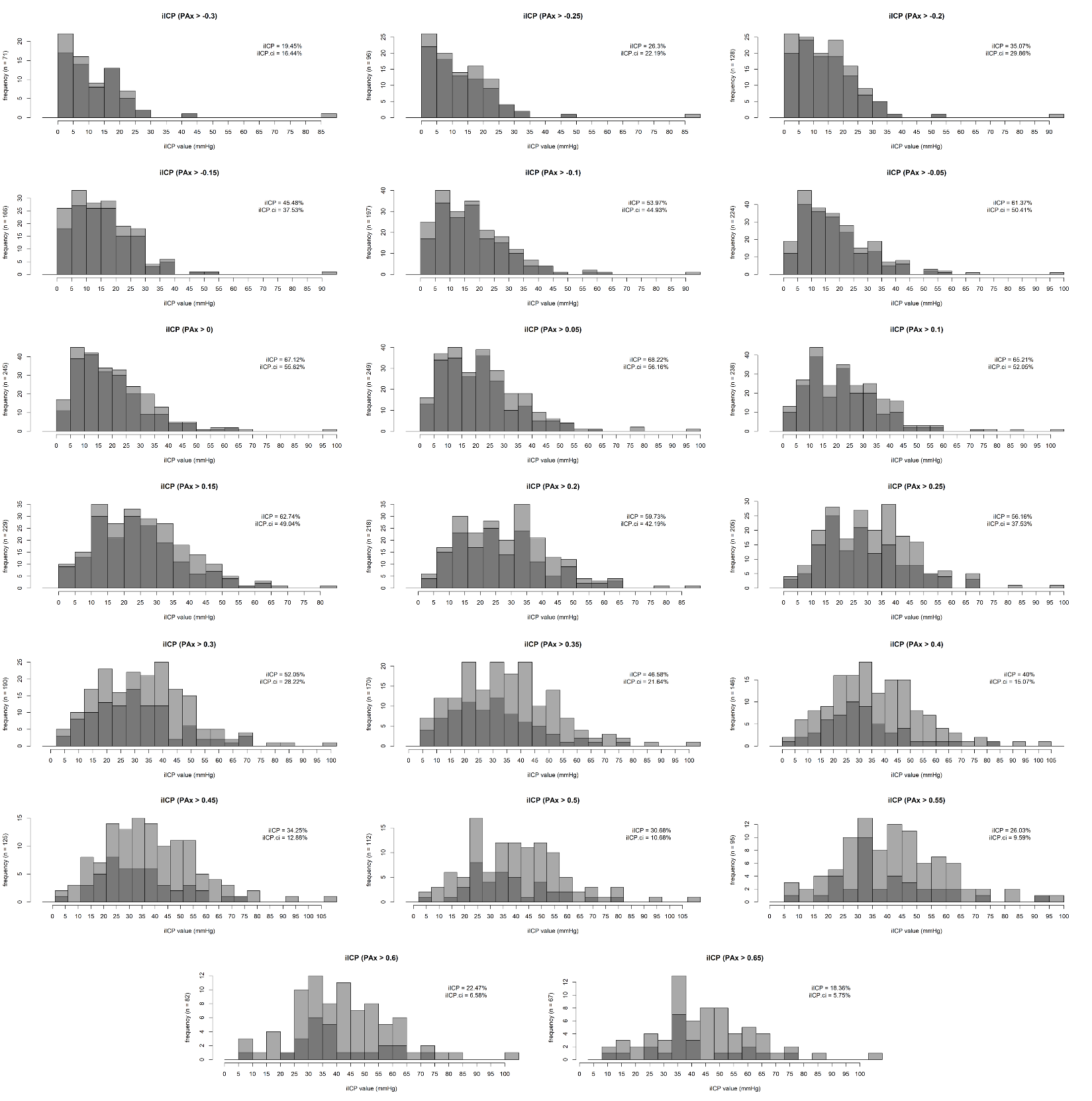


*AMP = pulse amplitude of ICP, ICP = intracranial pressure, iICP = individualized intracranial pressure thresholds, iICP.ci = individualized intracranial pressure thresholds with confidence intervals less than 0.2 a.u., MAP = mean arterial pressure, PAx = pulse amplitude index (correlation between AMP and MAP).*

*Histograms illustrate the distributions of the derived iICP values for each threshold that produced a percent yield greater than 15%. The dark grey bars indicate the proportion of derived iICP values that remain after filtering for those with confidence intervals less than 0.2 a.u. (denoted iICP.ci). Percent yields of iICP and iICP.ci for each threshold are listed on their respective plots.*

Supplemental Appendix C. Histograms of iICP derived using various RAC thresholds
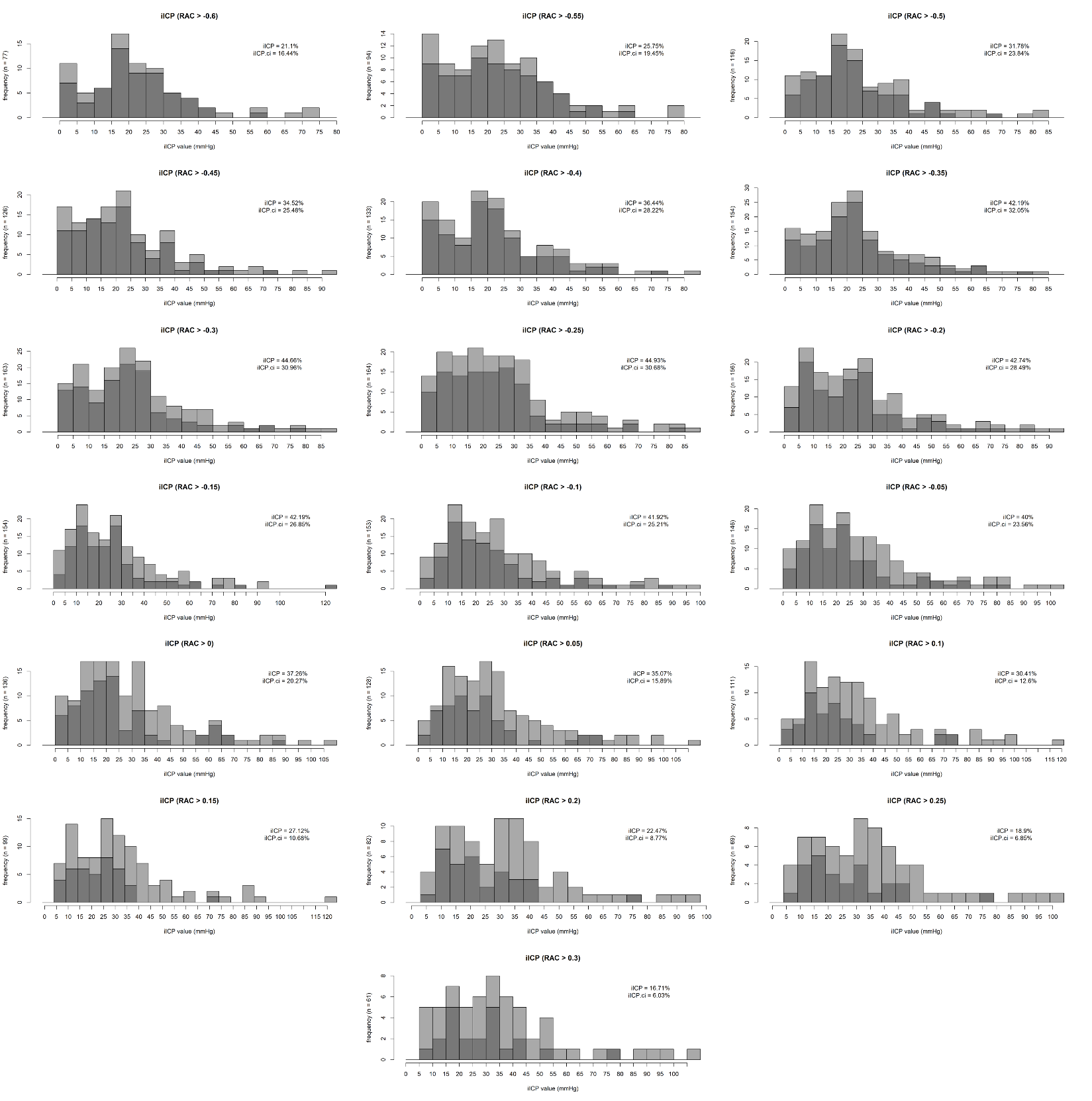


*AMP = pulse amplitude of ICP, CPP = cerebral perfusion pressure, ICP = intracranial pressure, iICP = individualized intracranial pressure thresholds, iICP.ci = individualized intracranial pressure thresholds with confidence intervals less than 0.2 a.u., RAC = correlation (R) between slow waves of AMP (A) and CPP (C).*

*Histograms illustrate the distributions of the derived iICP values for each threshold that produced a percent yield greater than 15%. The dark grey bars indicate the proportion of derived iICP values that remain after filtering for those with confidence intervals less than 0.2 a.u. (denoted iICP.ci). Percent yields of iICP and iICP.ci for each threshold are listed on their respective plots.*

Supplemental Appendix D. Chi-square outcome analysis of iICP derived using various PRx thresholds

| Threshold | % Yield | Alive vs Dead | | | Favorable vs Unfavorable | | |
| --- | --- | --- | --- | --- | --- | --- | --- |
|  |  | **χ^2^** | **p value** | **Adjusted p value** | **χ^2^** | **p value** | **Adjusted p value** |
| ICP > 20mmHg | --- | 22.90 | **<0.0001** | --- | 10.66 | **0.0011** | --- |
| -1.00 | 0.27 | --- | --- | --- | --- | --- | --- |
| -0.95 | 0.27 | --- | --- | --- | --- | --- | --- |
| -0.90 | 0.27 | --- | --- | --- | --- | --- | --- |
| -0.85 | 0.27 | --- | --- | --- | --- | --- | --- |
| -0.80 | 0.27 | --- | --- | --- | --- | --- | --- |
| -0.75 | 0.55 | --- | --- | --- | --- | --- | --- |
| -0.70 | 1.10 | --- | --- | --- | --- | --- | --- |
| -0.65 | 1.37 | --- | --- | --- | --- | --- | --- |
| -0.60 | 2.19 | --- | --- | --- | --- | --- | --- |
| -0.55 | 2.47 | --- | --- | --- | --- | --- | --- |
| -0.50 | 3.56 | --- | --- | --- | --- | --- | --- |
| -0.45 | 4.66 | --- | --- | --- | --- | --- | --- |
| -0.40 | 6.30 | --- | --- | --- | --- | --- | --- |
| -0.35 | 7.12 | --- | --- | --- | --- | --- | --- |
| -0.30 | 9.59 | --- | --- | --- | --- | --- | --- |
| -0.25 | 13.15 | --- | --- | --- | --- | --- | --- |
| -0.20 | 18.90 | 0.00 | 1.0000 | 1.0000 | 0.40 | 0.5270 | 0.6418 |
| -0.15 | 24.66 | 0.22 | 0.6360 | 0.7632 | 0.01 | 0.9214 | 0.9615 |
| -0.10 | 33.70 | 0.33 | 0.5636 | 0.7119 | 0.82 | 0.3657 | 0.5851 |
| -0.05 | 43.29 | 1.19 | 0.2751 | 0.5079 | 4.45 | 0.0350 | 0.1680 |
| 0.00 | 53.70 | 0.67 | 0.4143 | 0.6215 | 2.13 | 0.1442 | 0.4326 |
| 0.05 | 61.37 | 5.48 | **0.0193** | 0.3279 | 7.79 | **0.0053** | 0.0696 |
| 0.10 | 65.21 | 2.56 | 0.1093 | 0.3279 | 7.61 | **0.0058** | 0.0696 |
| 0.15 | 69.86 | 2.84 | 0.0917 | 0.3279 | 3.51 | 0.0610 | 0.2091 |
| 0.20 | 72.33 | 2.81 | 0.0939 | 0.3279 | 5.75 | **0.0165** | 0.1320 |
| 0.25 | 73.97 | 2.76 | 0.0967 | 0.3279 | 4.97 | **0.0258** | 0.1548 |
| 0.30 | 72.05 | 0.72 | 0.3967 | 0.6215 | 1.62 | 0.2025 | 0.4722 |
| 0.35 | 70.96 | 0.52 | 0.4725 | 0.6516 | 0.66 | 0.4161 | 0.5874 |
| 0.40 | 67.95 | 0.08 | 0.7804 | 0.8919 | 0.39 | 0.5348 | 0.6418 |
| 0.45 | 63.84 | 0.04 | 0.8329 | 0.9086 | 0.24 | 0.6270 | 0.7166 |
| 0.50 | 59.73 | 0.48 | 0.4887 | 0.6516 | 3.82 | 0.0505 | 0.2020 |
| 0.55 | 56.99 | 1.04 | 0.3082 | 0.5283 | 1.48 | 0.2231 | 0.4722 |
| 0.60 | 50.68 | 3.38 | 0.0661 | 0.3279 | 1.45 | 0.2290 | 0.4722 |
| 0.65 | 46.58 | 3.27 | 0.0706 | 0.3279 | 1.40 | 0.2361 | 0.4722 |
| 0.70 | 42.19 | 2.05 | 0.1521 | 0.4050 | 0.66 | 0.4156 | 0.5874 |
| 0.75 | 38.08 | 1.62 | 0.2025 | 0.4050 | 0.56 | 0.4530 | 0.6040 |
| 0.80 | 34.25 | 0.02 | 0.8930 | 0.9318 | 0.00 | 1.0000 | 1.0000 |
| 0.85 | 29.04 | 2.72 | 0.0989 | 0.3279 | 1.06 | 0.3023 | 0.5182 |
| 0.90 | 22.74 | 1.70 | 0.1917 | 0.4050 | 1.14 | 0.2855 | 0.5182 |
| 0.95 | 17.26 | 1.79 | 0.1814 | 0.4050 | 0.07 | 0.7893 | 0.8611 |
| 1.00 | 13.42 | --- | --- | --- | --- | --- | --- |

*Bolded p values are those reaching statistical significance, p < 0.05. Adjusted p values were calculated using the False Discovery Rate method. Grey rows represent the PRx range where cerebrovascular reactivity is generally considered to be intact.*

*ICP = intracranial pressure, iICP = individualized intracranial pressure thresholds, MAP = mean arterial pressure, PRx = pressure reactivity index (correlation between ICP and MAP), χ^2^ = chi-square value.*

Supplemental Appendix E. Chi-square outcome analysis of iICP derived using various PAx thresholds

| Threshold | % Yield | Alive vs Dead | | | Favorable vs Unfavorable | | |
| --- | --- | --- | --- | --- | --- | --- | --- |
|  |  | **χ^2^** | **p value** | **Adjusted p value** | **χ^2^** | **p value** | **Adjusted p value** |
| ICP > 20mmHg | --- | 22.90 | **<0.0001** |  | 10.66 | **0.0011** |  |
| -1.00 | 0 | --- | --- | --- | --- | --- | --- |
| -0.95 | 0 | --- | --- | --- | --- | --- | --- |
| -0.90 | 0 | --- | --- | --- | --- | --- | --- |
| -0.85 | 0 | --- | --- | --- | --- | --- | --- |
| -0.80 | 0.27 | --- | --- | --- | --- | --- | --- |
| -0.75 | 0.55 | --- | --- | --- | --- | --- | --- |
| -0.70 | 0.55 | --- | --- | --- | --- | --- | --- |
| -0.65 | 0.82 | --- | --- | --- | --- | --- | --- |
| -0.60 | 1.10 | --- | --- | --- | --- | --- | --- |
| -0.55 | 1.92 | --- | --- | --- | --- | --- | --- |
| -0.50 | 4.11 | --- | --- | --- | --- | --- | --- |
| -0.45 | 7.67 | --- | --- | --- | --- | --- | --- |
| -0.40 | 9.59 | --- | --- | --- | --- | --- | --- |
| -0.35 | 14.25 | --- | --- | --- | --- | --- | --- |
| -0.30 | 19.45 | 0.16 | 0.6889 | 0.7252 | 0.00 | 1.0000 | 1.0000 |
| -0.25 | 26.30 | 2.87 | 0.0903 | 0.1928 | 1.67 | 0.1959 | 0.2815 |
| -0.20 | 35.07 | 7.82 | **0.0052** | 0.0527 | 8.68 | 0.0032 | 0.0640 |
| -0.15 | 45.48 | 2.47 | 0.1157 | 0.1928 | 2.68 | 0.1017 | 0.2815 |
| -0.10 | 53.97 | 0.52 | 0.4714 | 0.5238 | 2.75 | 0.0973 | 0.2815 |
| -0.05 | 61.37 | 0.03 | 0.8645 | 0.8645 | 1.47 | 0.2252 | 0.2815 |
| 0.00 | 67.12 | 1.40 | 0.2375 | 0.2794 | 1.99 | 0.1585 | 0.2815 |
| 0.05 | 68.22 | 1.77 | 0.1828 | 0.2437 | 2.10 | 0.1472 | 0.2815 |
| 0.10 | 65.21 | 3.04 | 0.0810 | 0.1928 | 1.57 | 0.2107 | 0.2815 |
| 0.15 | 62.74 | 2.52 | 0.1121 | 0.1928 | 1.88 | 0.1699 | 0.2815 |
| 0.20 | 59.73 | 1.44 | 0.2307 | 0.2794 | 1.28 | 0.2588 | 0.3045 |
| 0.25 | 56.16 | 2.67 | 0.1021 | 0.1928 | 1.53 | 0.2155 | 0.2815 |
| 0.30 | 52.05 | 2.24 | 0.1342 | 0.2053 | 1.94 | 0.1637 | 0.2815 |
| 0.35 | 46.58 | 4.37 | **0.0366** | 0.1464 | 1.82 | 0.1769 | 0.2815 |
| 0.40 | 40.00 | 3.68 | 0.0549 | 0.1569 | 0.83 | 0.3615 | 0.3805 |
| 0.45 | 34.25 | 7.06 | **0.0079** | 0.0527 | 3.27 | 0.0707 | 0.2815 |
| 0.50 | 30.68 | 3.89 | **0.0487** | 0.1569 | 1.59 | 0.2068 | 0.2815 |
| 0.55 | 26.03 | 7.33 | **0.0068** | 0.0527 | 3.78 | 0.0519 | 0.2815 |
| 0.60 | 22.47 | 5.23 | **0.0222** | 0.1110 | 2.89 | 0.0889 | 0.2815 |
| 0.65 | 18.36 | 2.14 | 0.1437 | 0.2053 | 0.97 | 0.3237 | 0.3597 |
| 0.70 | 14.79 | --- | --- | --- | --- | --- | --- |
| 0.75 | 11.51 | --- | --- | --- | --- | --- | --- |
| 0.80 | 9.04 | --- | --- | --- | --- | --- | --- |
| 0.85 | 7.67 | --- | --- | --- | --- | --- | --- |
| 0.90 | 6.03 | --- | --- | --- | --- | --- | --- |
| 0.95 | 4.38 | --- | --- | --- | --- | --- | --- |
| 1.00 | 3.01 | --- | --- | --- | --- | --- | --- |

*Bolded p values are those reaching statistical significance, p < 0.05. Adjusted p values were calculated using the False Discovery Rate method. Grey rows represent the PAx range where cerebrovascular reactivity is generally considered to be intact.*

*AMP = pulse amplitude of ICP, ICP = intracranial pressure, iICP = individualized intracranial pressure thresholds, MAP = mean arterial pressure, PAx = pulse amplitude index (correlation between AMP and MAP), χ^2^ = chi-square value.*

Supplemental Appendix F. Chi-square outcome analysis of iICP derived using various RAC thresholds

| Threshold | % Yield | Alive vs Dead | | | Favorable vs Unfavorable | | |
| --- | --- | --- | --- | --- | --- | --- | --- |
|  |  | **χ^2^** | **p value** | **Adjusted p value** | **χ^2^** | **p value** | **Adjusted p value** |
| ICP > 20mmHg | --- | 22.90 | **<0.0001** | --- | 10.66 | **0.0011** | **---** |
| -1.00 | 0.55 | --- | --- | --- | --- | --- | --- |
| -0.95 | 0.82 | --- | --- | --- | --- | --- | --- |
| -0.90 | 0.82 | --- | --- | --- | --- | --- | --- |
| -0.85 | 1.10 | --- | --- | --- | --- | --- | --- |
| -0.80 | 2.74 | --- | --- | --- | --- | --- | --- |
| -0.75 | 6.58 | --- | --- | --- | --- | --- | --- |
| -0.70 | 10.68 | --- | --- | --- | --- | --- | --- |
| -0.65 | 14.79 | --- | --- | --- | --- | --- | --- |
| -0.60 | 21.10 | 0.18 | 0.6723 | 0.8877 | 0.00 | 1.0000 | 1.0000 |
| -0.55 | 25.75 | 0.00 | 1.0000 | 1.0000 | 0.02 | 0.8990 | 1.0000 |
| -0.50 | 31.78 | 3.12 | 0.0772 | 0.2445 | 0.25 | 0.6187 | 0.9043 |
| -0.45 | 34.52 | 12.31 | **0.0004** | **0.0076** | 4.51 | **0.0337** | 0.6403 |
| -0.40 | 36.44 | 5.74 | **0.0165** | 0.1045 | 1.88 | 0.1705 | 0.8099 |
| -0.35 | 42.19 | 1.87 | 0.1712 | 0.3637 | 0.33 | 0.5678 | 0.899 |
| -0.30 | 44.66 | 6.56 | **0.0104** | 0.0988 | 1.18 | 0.2775 | 0.8564 |
| -0.25 | 44.93 | 3.99 | **0.0457** | 0.2171 | 2.15 | 0.1421 | 0.8099 |
| -0.20 | 42.74 | 0.64 | 0.4239 | 0.7322 | 1.01 | 0.3155 | 0.8564 |
| -0.15 | 42.19 | 0.00 | 1.0000 | 1.0000 | 0.13 | 0.7236 | 0.9820 |
| -0.10 | 41.92 | 0.16 | 0.6929 | 0.8877 | 1.96 | 0.1620 | 0.8099 |
| -0.05 | 40.00 | 0.01 | 0.9159 | 1.0000 | 0.00 | 1.0000 | 1.0000 |
| 0.00 | 37.26 | 0.15 | 0.7008 | 0.8877 | 0.40 | 0.5280 | 0.8990 |
| 0.05 | 35.07 | 0.00 | 1.0000 | 1.0000 | 0.00 | 0.9688 | 1.0000 |
| 0.10 | 30.41 | 0.40 | 0.5275 | 0.8352 | 0.01 | 0.9352 | 1.0000 |
| 0.15 | 27.12 | 1.86 | 0.1723 | 0.3637 | 0.67 | 0.4125 | 0.8990 |
| 0.20 | 22.47 | 1.39 | 0.2377 | 0.4516 | 0.60 | 0.4377 | 0.8990 |
| 0.25 | 18.90 | 2.33 | 0.1268 | 0.3442 | 0.36 | 0.5483 | 0.8990 |
| 0.30 | 16.71 | 3.24 | 0.0721 | 0.2445 | 1.28 | 0.2586 | 0.8564 |
| 0.35 | 14.52 | --- | --- | --- | --- | --- | --- |
| 0.40 | 11.51 | --- | --- | --- | --- | --- | --- |
| 0.45 | 9.32 | --- | --- | --- | --- | --- | --- |
| 0.50 | 9.32 | --- | --- | --- | --- | --- | --- |
| 0.55 | 8.77 | --- | --- | --- | --- | --- | --- |
| 0.60 | 8.22 | --- | --- | --- | --- | --- | --- |
| 0.65 | 7.40 | --- | --- | --- | --- | --- | --- |
| 0.70 | 5.75 | --- | --- | --- | --- | --- | --- |
| 0.75 | 3.84 | --- | --- | --- | --- | --- | --- |
| 0.80 | 1.37 | --- | --- | --- | --- | --- | --- |
| 0.85 | 1.37 | --- | --- | --- | --- | --- | --- |
| 0.90 | 1.37 | --- | --- | --- | --- | --- | --- |
| 0.95 | 1.10 | --- | --- | --- | --- | --- | --- |
| 1.00 | 0.82 | --- | --- | --- | --- | --- | --- |

*Bolded p values are those reaching statistical significance, p < 0.05. Adjusted p values were calculated using the False Discovery Rate method. Grey rows represent the RAC range where cerebrovascular reactivity is generally considered to be intact.*

*AMP = pulse amplitude of ICP, CPP = cerebral perfusion pressure, ICP = intracranial pressure, iICP = individualized intracranial pressure thresholds, RAC = correlation (R) between slow waves of AMP (A) and CPP (C), χ^2^ = chi-square value.*

Supplemental Appendix G. Chi-square outcome analysis of iICP.ci derived using various PRx thresholds

| Threshold | % Yield | Alive vs Dead | | | Favorable vs Unfavorable | | |
| --- | --- | --- | --- | --- | --- | --- | --- |
|  |  | **χ^2^** | **p value** | **Adjusted p value** | **χ^2^** | **p value** | **Adjusted p value** |
| ICP > 20mmHg | --- | 22.90 | **<0.0001** | --- | 10.66 | **0.0011** | **---** |
| -1.00 | 0 | --- | --- | --- | --- | --- | --- |
| -0.95 | 0 | --- | --- | --- | --- | --- | --- |
| -0.90 | 0 | --- | --- | --- | --- | --- | --- |
| -0.85 | 0 | --- | --- | --- | --- | --- | --- |
| -0.80 | 0 | --- | --- | --- | --- | --- | --- |
| -0.75 | 0 | --- | --- | --- | --- | --- | --- |
| -0.70 | 0 | --- | --- | --- | --- | --- | --- |
| -0.65 | 0 | --- | --- | --- | --- | --- | --- |
| -0.60 | 0 | --- | --- | --- | --- | --- | --- |
| -0.55 | 0.27 | --- | --- | --- | --- | --- | --- |
| -0.50 | 0.27 | --- | --- | --- | --- | --- | --- |
| -0.45 | 1.64 | --- | --- | --- | --- | --- | --- |
| -0.40 | 2.47 | --- | --- | --- | --- | --- | --- |
| -0.35 | 3.84 | --- | --- | --- | --- | --- | --- |
| -0.30 | 4.66 | --- | --- | --- | --- | --- | --- |
| -0.25 | 8.49 | --- | --- | --- | --- | --- | --- |
| -0.20 | 14.52 | --- | --- | --- | --- | --- | --- |
| -0.15 | 19.45 | 0.27 | 0.6040 | 0.6938 | 0.00 | 1.0000 | 1.0000 |
| -0.10 | 27.95 | 0.57 | 0.4490 | 0.6361 | 0.4 | 0.5270 | 0.6892 |
| -0.05 | 37.53 | 1.03 | 0.3095 | 0.5846 | 2.58 | 0.1080 | 0.2623 |
| 0.00 | 47.40 | 0.66 | 0.4152 | 0.6361 | 1.99 | 0.1588 | 0.3000 |
| 0.05 | 54.79 | 5.86 | **0.0155** | 0.2635 | 8.10 | **0.0044** | **0.0374** |
| 0.10 | 58.08 | 3.02 | 0.0824 | 0.3502 | 9.98 | **0.0016** | **0.0272** |
| 0.15 | 59.73 | 2.09 | 0.1487 | 0.4213 | 3.41 | 0.0649 | 0.1839 |
| 0.20 | 61.10 | 2.23 | 0.1357 | 0.4213 | 6.01 | **0.0142** | 0.0805 |
| 0.25 | 61.64 | 1.64 | 0.2001 | 0.4860 | 3.77 | 0.0521 | 0.1771 |
| 0.30 | 56.16 | 0.26 | 0.6122 | 0.6938 | 0.80 | 0.3725 | 0.5277 |
| 0.35 | 52.60 | 0.27 | 0.6010 | 0.6938 | 0.25 | 0.6155 | 0.7474 |
| 0.40 | 48.77 | 0.02 | 0.9007 | 0.9570 | 0.02 | 0.9015 | 0.9578 |
| 0.45 | 42.74 | 0.00 | 1.0000 | 1.0000 | 0.03 | 0.8709 | 0.9578 |
| 0.50 | 36.71 | 0.65 | 0.4201 | 0.6361 | 4.63 | **0.0314** | 0.1334 |
| 0.55 | 31.23 | 1.31 | 0.2533 | 0.5383 | 2.01 | 0.1558 | 0.3000 |
| 0.60 | 25.21 | 3.30 | 0.0693 | 0.3502 | 1.76 | 0.1849 | 0.3143 |
| 0.65 | 18.36 | 3.21 | 0.0733 | 0.3502 | 1.58 | 0.2081 | 0.3216 |
| 0.70 | 14.79 | --- | --- | --- | --- | --- | --- |
| 0.75 | 11.23 | --- | --- | --- | --- | --- | --- |
| 0.80 | 7.95 | --- | --- | --- | --- | --- | --- |
| 0.85 | 6.03 | --- | --- | --- | --- | --- | --- |
| 0.90 | 2.74 | --- | --- | --- | --- | --- | --- |
| 0.95 | 0.55 | --- | --- | --- | --- | --- | --- |
| 1.00 | 0.27 | --- | --- | --- | --- | --- | --- |

*Bolded p values are those reaching statistical significance, p < 0.05. Adjusted p values were calculated using the False Discovery Rate method. Grey rows represent the PRx range where cerebrovascular reactivity is generally considered to be intact.*

*ICP = intracranial pressure, iICP.ci = individualized intracranial pressure thresholds with confidence intervals less than 0.2 a.u., MAP = mean arterial pressure, PRx = pressure reactivity index (correlation between ICP and MAP), χ^2^ = chi-square value.*

Supplemental Appendix H. Chi-square outcome analysis of iICP.ci derived using various PAx thresholds

| Threshold | % Yield | Alive vs Dead | | | Favorable vs Unfavorable | | |
| --- | --- | --- | --- | --- | --- | --- | --- |
|  |  | **χ^2^** | **p value** | **Adjusted p value** | **χ^2^** | **p value** | **Adjusted p value** |
| ICP > 20mmHg | --- | 22.90 | **<0.0001** | --- | 10.66 | **0.0011** | **---** |
| -1.00 | 0 | --- | --- | --- | --- | --- | --- |
| -0.95 | 0 | --- | --- | --- | --- | --- | --- |
| -0.90 | 0 | --- | --- | --- | --- | --- | --- |
| -0.85 | 0 | --- | --- | --- | --- | --- | --- |
| -0.80 | 0 | --- | --- | --- | --- | --- | --- |
| -0.75 | 0 | --- | --- | --- | --- | --- | --- |
| -0.70 | 0 | --- | --- | --- | --- | --- | --- |
| -0.65 | 0.27 | --- | --- | --- | --- | --- | --- |
| -0.60 | 0.27 | --- | --- | --- | --- | --- | --- |
| -0.55 | 0.82 | --- | --- | --- | --- | --- | --- |
| -0.50 | 2.47 | --- | --- | --- | --- | --- | --- |
| -0.45 | 4.93 | --- | --- | --- | --- | --- | --- |
| -0.40 | 6.58 | --- | --- | --- | --- | --- | --- |
| -0.35 | 11.23 | --- | --- | --- | --- | --- | --- |
| -0.30 | 16.44 | 1.17 | 0.2790 | 0.3541 | 0.28 | 0.5986 | 0.5986 |
| -0.25 | 22.19 | 3.22 | 0.0728 | 0.2779 | 1.26 | 0.2625 | 0.3873 |
| -0.20 | 29.86 | 10.19 | **0.0014** | **0.0210** | 9.43 | **0.0021** | **0.0315** |
| -0.15 | 37.53 | 2.43 | 0.1188 | 0.2779 | 1.62 | 0.2030 | 0.3873 |
| -0.10 | 44.93 | 0.52 | 0.4704 | 0.5405 | 2.68 | 0.1015 | 0.3873 |
| -0.05 | 50.41 | 0.00 | 1.0000 | 1.0000 | 1.30 | 0.2546 | 0.3873 |
| 0.00 | 55.62 | 0.45 | 0.5045 | 0.5405 | 1.30 | 0.2542 | 0.3873 |
| 0.05 | 56.16 | 1.27 | 0.2596 | 0.3541 | 2.14 | 0.1431 | 0.3873 |
| 0.10 | 52.05 | 2.30 | 0.1297 | 0.2779 | 1.02 | 0.3135 | 0.3919 |
| 0.15 | 49.04 | 2.51 | 0.1130 | 0.2779 | 1.78 | 0.1824 | 0.3873 |
| 0.20 | 42.19 | 1.15 | 0.2833 | 0.3541 | 1.15 | 0.2840 | 0.3873 |
| 0.25 | 37.53 | 1.93 | 0.1649 | 0.3092 | 1.52 | 0.2172 | 0.3873 |
| 0.30 | 28.22 | 1.59 | 0.2073 | 0.3455 | 1.62 | 0.2031 | 0.3873 |
| 0.35 | 21.64 | 2.96 | 0.0852 | 0.2779 | 0.82 | 0.3640 | 0.4200 |
| 0.40 | 15.07 | 2.66 | 0.1030 | 0.2779 | 0.57 | 0.4490 | 0.4811 |
| 0.45 | 12.88 | --- | --- | --- | --- | --- | --- |
| 0.50 | 10.68 | --- | --- | --- | --- | --- | --- |
| 0.55 | 9.59 | --- | --- | --- | --- | --- | --- |
| 0.60 | 6.58 | --- | --- | --- | --- | --- | --- |
| 0.65 | 5.75 | --- | --- | --- | --- | --- | --- |
| 0.70 | 4.93 | --- | --- | --- | --- | --- | --- |
| 0.75 | 3.01 | --- | --- | --- | --- | --- | --- |
| 0.80 | 1.37 | --- | --- | --- | --- | --- | --- |
| 0.85 | 1.10 | --- | --- | --- | --- | --- | --- |
| 0.90 | 0.82 | --- | --- | --- | --- | --- | --- |
| 0.95 | 0.27 | --- | --- | --- | --- | --- | --- |
| 1.00 | 0 | --- | --- | --- | --- | --- | --- |

*Bolded p values are those reaching statistical significance, p < 0.05. Adjusted p values were calculated using the False Discovery Rate method. Grey rows represent the PAx range where cerebrovascular reactivity is generally considered to be intact.*

*AMP = pulse amplitude of ICP, ICP = intracranial pressure, iICP.ci = individualized intracranial pressure thresholds with confidence intervals less than 0.2 a.u., MAP = mean arterial pressure, PAx = pulse amplitude index (correlation between AMP and MAP), χ^2^ = chi-square value.*

Supplemental Appendix I. Chi-square outcome analysis of iICP.ci derived using various RAC thresholds

| Threshold | % Yield | Alive vs Dead | | | Favorable vs Unfavorable | | |
| --- | --- | --- | --- | --- | --- | --- | --- |
|  |  | **χ^2^** | **p value** | **Adjusted p value** | **χ^2^** | **p value** | **Adjusted p value** |
| ICP > 20mmHg | --- | 22.90 | **<0.0001** | --- | 10.66 | **0.0011** | **---** |
| -1.00 | 0 | --- | --- | --- | --- | --- | --- |
| -0.95 | 0 | --- | --- | --- | --- | --- | --- |
| -0.90 | 0 | --- | --- | --- | --- | --- | --- |
| -0.85 | 0.55 | --- | --- | --- | --- | --- | --- |
| -0.80 | 1.37 | --- | --- | --- | --- | --- | --- |
| -0.75 | 4.66 | --- | --- | --- | --- | --- | --- |
| -0.70 | 7.95 | --- | --- | --- | --- | --- | --- |
| -0.65 | 10.96 | --- | --- | --- | --- | --- | --- |
| -0.60 | 16.44 | 0.82 | 0.3658 | 0.7466 | 0.00 | 1.0000 | 1.0000 |
| -0.55 | 19.45 | 0.00 | 1.0000 | 1.0000 | 0.01 | 0.9244 | 1.0000 |
| -0.50 | 23.84 | 2.71 | 0.0996 | 0.3486 | 0.53 | 0.4673 | 0.7991 |
| -0.45 | 25.48 | 16.33 | **0.0001** | **0.0014** | 7.43 | **0.0064** | 0.0896 |
| -0.40 | 28.22 | 6.15 | **0.0132** | 0.0924 | 2.44 | 0.1183 | 0.5521 |
| -0.35 | 32.05 | 2.12 | 0.1457 | 0.4080 | 0.90 | 0.3415 | 0.6830 |
| -0.30 | 30.96 | 4.00 | **0.0454** | 0.2119 | 1.23 | 0.2675 | 0.6830 |
| -0.25 | 30.68 | 0.79 | 0.3733 | 0.7466 | 0.91 | 0.3401 | 0.6830 |
| -0.20 | 28.49 | 0.09 | 0.7688 | 1.0000 | 1.02 | 0.3125 | 0.6830 |
| -0.15 | 26.85 | 0.00 | 1.0000 | 1.0000 | 0.32 | 0.5708 | 0.7991 |
| -0.10 | 25.21 | 0.00 | 1.0000 | 1.0000 | 2.93 | 0.0869 | 0.5521 |
| -0.05 | 23.56 | 0.00 | 1.0000 | 1.0000 | 0.00 | 1.0000 | 1.0000 |
| 0.00 | 20.27 | 0.00 | 1.0000 | 1.0000 | 0.33 | 0.5646 | 0.7991 |
| 0.05 | 15.89 | 0.00 | 0.9543 | 1.0000 | 0.00 | 1.0000 | 1.0000 |
| 0.10 | 12.60 | --- | --- | --- | --- | --- | --- |
| 0.15 | 10.68 | --- | --- | --- | --- | --- | --- |
| 0.20 | 8.77 | --- | --- | --- | --- | --- | --- |
| 0.25 | 6.85 | --- | --- | --- | --- | --- | --- |
| 0.30 | 6.03 | --- | --- | --- | --- | --- | --- |
| 0.35 | 4.93 | --- | --- | --- | --- | --- | --- |
| 0.40 | 3.29 | --- | --- | --- | --- | --- | --- |
| 0.45 | 3.01 | --- | --- | --- | --- | --- | --- |
| 0.50 | 2.74 | --- | --- | --- | --- | --- | --- |
| 0.55 | 2.47 | --- | --- | --- | --- | --- | --- |
| 0.60 | 2.19 | --- | --- | --- | --- | --- | --- |
| 0.65 | 2.19 | --- | --- | --- | --- | --- | --- |
| 0.70 | 1.10 | --- | --- | --- | --- | --- | --- |
| 0.75 | 0.55 | --- | --- | --- | --- | --- | --- |
| 0.80 | 0.27 | --- | --- | --- | --- | --- | --- |
| 0.85 | 0.27 | --- | --- | --- | --- | --- | --- |
| 0.90 | 0.27 | --- | --- | --- | --- | --- | --- |
| 0.95 | 0 | --- | --- | --- | --- | --- | --- |
| 1.00 | 0 | --- | --- | --- | --- | --- | --- |

*Bolded p values are those reaching statistical significance, p < 0.05. Adjusted p values were calculated using the False Discovery Rate method. Grey rows represent the RAC range where cerebrovascular reactivity is generally considered to be intact.*

*AMP = pulse amplitude of ICP, CPP = cerebral perfusion pressure, ICP = intracranial pressure, iICP.ci = individualized intracranial pressure thresholds with confidence intervals less than 0.2 a.u., RAC = correlation (R) between slow waves of AMP (A) and CPP (C), χ^2^ = chi-square value.*

Supplemental Appendix J. Chi-square outcome plots of iICP.ci derived using various cerebrovascular reactivity thresholds

*
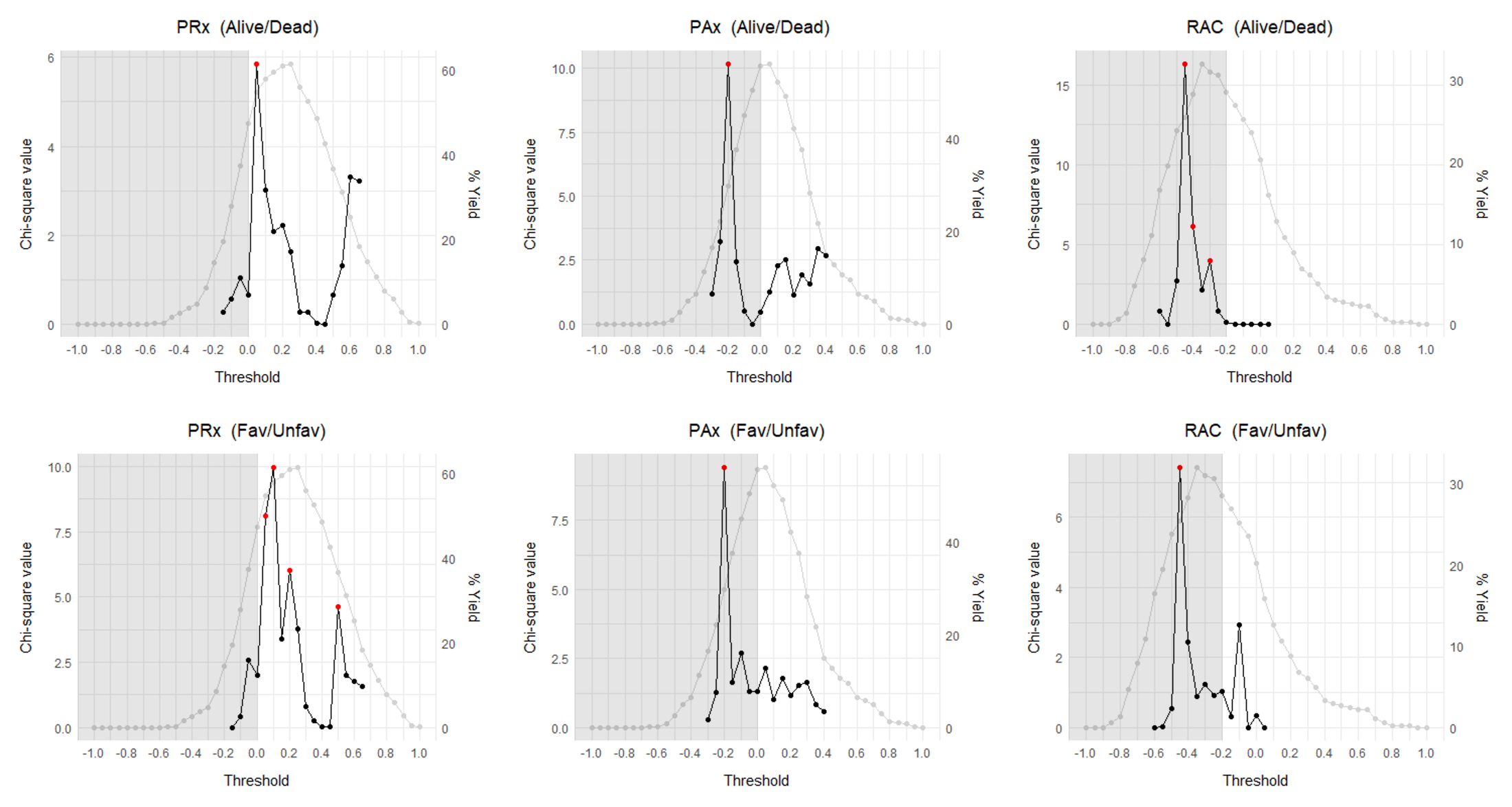
AMP = pulse amplitude of ICP, CPP = cerebral perfusion pressure, ICP = intracranial pressure, iICP.ci = individualized intracranial pressure thresholds with confidence intervals less than 0.2 a.u., MAP = mean arterial pressure, PAx = pulse amplitude index (correlation between AMP and MAP), PRx = pressure reactivity index (correlation between ICP and MAP), RAC = correlation (R) between slow waves of AMP (A) and CPP (C).*

*The grey points illustrate the percent yields of each threshold in deriving iICP.ci. The black and red points illustrate the chi-square values for predicting outcome (Alive vs Dead or Favorable vs Unfavorable) of each threshold that produced a percent yield greater than 15%. Red points indicate thresholds that reached statistical significance, p < 0.05. Greyed out area represents the range where cerebrovascular reactivity is generally considered to be intact.*

Supplemental Appendix K. Spearman rank correlation analysis between iICP.ci derived using various PRx thresholds and measures of cerebral physiologic insult burden

| Threshold | % Yield | % time with CPP < 60mmHg | | % time with CPP > 70mmHg | | % time with PAx > 0.25 | | % time with RAC > 0 | | % time with PbtO_2_ < 20mmHg | |
| --- | --- | --- | --- | --- | --- | --- | --- | --- | --- | --- | --- |
|  |  | Coefficient | p value | Coefficient | p value | Coefficient | p value | Coefficient | p value | Coefficient | p value |
| ICP > 20mmHg | --- | 0.25 | **<0.0001** | -0.02 | 0.7182 | 0.14 | **0.0066** | -0.14 | **0.0068** | -0.09 | 0.3384 |
| -1.00 | 0 | --- | --- | --- | --- | --- | --- | --- | --- | --- | --- |
| -0.95 | 0 | --- | --- | --- | --- | --- | --- | --- | --- | --- | --- |
| -0.90 | 0 | --- | --- | --- | --- | --- | --- | --- | --- | --- | --- |
| -0.85 | 0 | --- | --- | --- | --- | --- | --- | --- | --- | --- | --- |
| -0.80 | 0 | --- | --- | --- | --- | --- | --- | --- | --- | --- | --- |
| -0.75 | 0 | --- | --- | --- | --- | --- | --- | --- | --- | --- | --- |
| -0.70 | 0 | --- | --- | --- | --- | --- | --- | --- | --- | --- | --- |
| -0.65 | 0 | --- | --- | --- | --- | --- | --- | --- | --- | --- | --- |
| -0.60 | 0 | --- | --- | --- | --- | --- | --- | --- | --- | --- | --- |
| -0.55 | 0.27 | --- | --- | --- | --- | --- | --- | --- | --- | --- | --- |
| -0.50 | 0.27 | --- | --- | --- | --- | --- | --- | --- | --- | --- | --- |
| -0.45 | 1.64 | --- | --- | --- | --- | --- | --- | --- | --- | --- | --- |
| -0.40 | 2.47 | --- | --- | --- | --- | --- | --- | --- | --- | --- | --- |
| -0.35 | 3.84 | --- | --- | --- | --- | --- | --- | --- | --- | --- | --- |
| -0.30 | 4.66 | --- | --- | --- | --- | --- | --- | --- | --- | --- | --- |
| -0.25 | 8.49 | --- | --- | --- | --- | --- | --- | --- | --- | --- | --- |
| -0.20 | 14.52 | --- | --- | --- | --- | --- | --- | --- | --- | --- | --- |
| -0.15 | 19.45 | 0.01 | 0.9630 | 0.00 | 0.9868 | 0.61 | **<0.0001** | 0.29 | **0.0136** | -0.06 | 0.7728 |
| -0.10 | 27.95 | 0.09 | 0.3885 | -0.10 | 0.3310 | 0.59 | **<0.0001** | 0.33 | **0.0007** | -0.01 | 0.9661 |
| -0.05 | 37.53 | 0.16 | 0.0544 | -0.17 | 0.0520 | 0.55 | **<0.0001** | 0.30 | **0.0003** | 0.08 | 0.6127 |
| 0.00 | 47.40 | 0.25 | **0.0011** | -0.22 | **0.0031** | 0.51 | **<0.0001** | 0.28 | **0.0002** | 0.05 | 0.7117 |
| 0.05 | 54.79 | 0.20 | **0.0042** | -0.22 | **0.0014** | 0.48 | **<0.0001** | 0.25 | **0.0004** | 0.02 | 0.8557 |
| 0.10 | 58.08 | 0.20 | **0.0042** | -0.20 | **0.0042** | 0.48 | **<0.0001** | 0.25 | **0.0002** | 0.10 | 0.3976 |
| 0.15 | 59.73 | 0.23 | **0.0005** | -0.19 | **0.0040** | 0.46 | **<0.0001** | 0.24 | **0.0003** | 0.00 | 0.9772 |
| 0.20 | 61.10 | 0.19 | **0.0043** | -0.16 | **0.0182** | 0.49 | **<0.0001** | 0.28 | **<0.0001** | 0.06 | 0.6118 |
| 0.25 | 61.64 | 0.16 | **0.0138** | -0.14 | **0.0371** | 0.48 | **<0.0001** | 0.31 | **<0.0001** | 0.06 | 0.6139 |
| 0.30 | 56.16 | 0.14 | **0.0434** | -0.10 | 0.1523 | 0.50 | **<0.0001** | 0.36 | **<0.0001** | 0.05 | 0.7148 |
| 0.35 | 52.60 | 0.24 | **0.0008** | -0.18 | **0.0124** | 0.50 | **<0.0001** | 0.39 | **<0.0001** | -0.05 | 0.7231 |
| 0.40 | 48.77 | 0.21 | **0.0047** | -0.18 | **0.0186** | 0.42 | **<0.0001** | 0.33 | **<0.0001** | -0.13 | 0.3459 |
| 0.45 | 42.74 | 0.19 | **0.0163** | -0.15 | 0.0631 | 0.44 | **<0.0001** | 0.39 | **<0.0001** | -0.02 | 0.8825 |
| 0.50 | 36.71 | 0.19 | **0.0297** | -0.18 | **0.0403** | 0.43 | **<0.0001** | 0.44 | **<0.0001** | 0.01 | 0.9552 |
| 0.55 | 31.23 | 0.26 | **0.0060** | -0.23 | **0.0154** | 0.49 | **<0.0001** | 0.50 | **<0.0001** | -0.06 | 0.7406 |
| 0.60 | 25.21 | 0.35 | **0.0005** | -0.33 | **0.0016** | 0.43 | **<0.0001** | 0.46 | **<0.0001** | -0.23 | 0.2263 |
| 0.65 | 18.36 | 0.42 | **0.0004** | -0.43 | **0.0003** | 0.45 | **0.0002** | 0.52 | **<0.0001** | -0.22 | 0.3607 |
| 0.70 | 14.79 | --- | --- | --- | --- | --- | --- | --- | --- | --- | --- |
| 0.75 | 11.23 | --- | --- | --- | --- | --- | --- | --- | --- | --- | --- |
| 0.80 | 7.95 | --- | --- | --- | --- | --- | --- | --- | --- | --- | --- |
| 0.85 | 6.03 | --- | --- | --- | --- | --- | --- | --- | --- | --- | --- |
| 0.90 | 2.74 | --- | --- | --- | --- | --- | --- | --- | --- | --- | --- |
| 0.95 | 0.55 | --- | --- | --- | --- | --- | --- | --- | --- | --- | --- |
| 1.00 | 0.27 | --- | --- | --- | --- | --- | --- | --- | --- | --- | --- |

Bolded p values are those reaching statistical significance, p < 0.05. *Grey rows represent the PRx range where cerebrovascular reactivity is generally considered to be intact.*

*AMP = pulse amplitude of ICP, CPP = cerebral perfusion pressure, ICP = intracranial pressure, iICP.ci = individualized intracranial pressure thresholds with confidence intervals less than 0.2 a.u., MAP = mean arterial pressure, mmHg = millimeters of mercury, PAx = pulse amplitude index (correlation between AMP and MAP), PbtO2 = brain tissue oxygen tension, PRx = pressure reactivity index (correlation between ICP and MAP), RAC = correlation (R) between slow waves of AMP (A) and CPP (C).*

Supplemental Appendix L. Spearman rank correlation analysis between iICP.ci derived using various PAx thresholds and measures of cerebral physiologic insult burden

| Threshold | % Yield | % time with CPP < 60mmHg | | % time with CPP > 70mmHg | | % time with PRx > 0.25 | | % time with RAC > 0 | | % time with PbtO_2_ < 20mmHg | |
| --- | --- | --- | --- | --- | --- | --- | --- | --- | --- | --- | --- |
|  |  | Coefficient | p value | Coefficient | p value | Coefficient | p value | Coefficient | p value | Coefficient | p value |
| ICP > 20mmHg | --- | 0.25 | **<0.0001** | -0.02 | 0.7182 | 0.14 | **0.0066** | -0.14 | **0.0068** | -0.09 | 0.3384 |
| -1.00 | 0 | --- | --- | --- | --- | --- | --- | --- | --- | --- | --- |
| -0.95 | 0 | --- | --- | --- | --- | --- | --- | --- | --- | --- | --- |
| -0.90 | 0 | --- | --- | --- | --- | --- | --- | --- | --- | --- | --- |
| -0.85 | 0 | --- | --- | --- | --- | --- | --- | --- | --- | --- | --- |
| -0.80 | 0 | --- | --- | --- | --- | --- | --- | --- | --- | --- | --- |
| -0.75 | 0 | --- | --- | --- | --- | --- | --- | --- | --- | --- | --- |
| -0.70 | 0 | --- | --- | --- | --- | --- | --- | --- | --- | --- | --- |
| -0.65 | 0.27 | --- | --- | --- | --- | --- | --- | --- | --- | --- | --- |
| -0.60 | 0.27 | --- | --- | --- | --- | --- | --- | --- | --- | --- | --- |
| -0.55 | 0.82 | --- | --- | --- | --- | --- | --- | --- | --- | --- | --- |
| -0.50 | 2.47 | --- | --- | --- | --- | --- | --- | --- | --- | --- | --- |
| -0.45 | 4.93 | --- | --- | --- | --- | --- | --- | --- | --- | --- | --- |
| -0.40 | 6.58 | --- | --- | --- | --- | --- | --- | --- | --- | --- | --- |
| -0.35 | 11.23 | --- | --- | --- | --- | --- | --- | --- | --- | --- | --- |
| -0.30 | 16.44 | -0.04 | 0.7690 | 0.04 | 0.7895 | 0.36 | **0.0044** | 0.27 | **0.0353** | -0.27 | 0.2292 |
| -0.25 | 22.19 | 0.08 | 0.4819 | -0.05 | 0.6438 | 0.35 | **0.0012** | 0.38 | **0.0006** | -0.19 | 0.3315 |
| -0.20 | 29.86 | 0.20 | **0.0334** | -0.13 | 0.1847 | 0.36 | **0.0002** | 0.23 | **0.0186** | -0.21 | 0.2454 |
| -0.15 | 37.53 | 0.15 | 0.0902 | -0.10 | 0.2276 | 0.37 | **<0.0001** | 0.20 | **0.0165** | -0.06 | 0.6936 |
| -0.10 | 44.93 | 0.17 | **0.0265** | -0.10 | 0.1808 | 0.46 | **<0.0001** | 0.32 | **<0.0001** | -0.19 | 0.1540 |
| -0.05 | 50.41 | 0.19 | **0.0082** | -0.13 | 0.0880 | 0.49 | **<0.0001** | 0.35 | **<0.0001** | -0.16 | 0.2207 |
| 0.00 | 55.62 | 0.23 | **0.0010** | -0.16 | **0.0194** | 0.55 | **<0.0001** | 0.32 | **<0.0001** | -0.12 | 0.3564 |
| 0.05 | 56.16 | 0.28 | **<0.0001** | -0.23 | **0.0008** | 0.57 | **<0.0001** | 0.37 | **<0.0001** | -0.11 | 0.3600 |
| 0.10 | 52.05 | 0.23 | **0.0014** | -0.24 | **0.0010** | 0.57 | **<0.0001** | 0.42 | **<0.0001** | 0.02 | 0.8702 |
| 0.15 | 49.04 | 0.19 | **0.0130** | -0.18 | **0.0145** | 0.55 | **<0.0001** | 0.41 | **<0.0001** | 0.01 | 0.9656 |
| 0.20 | 42.19 | 0.20 | **0.0152** | -0.14 | 0.0817 | 0.57 | **<0.0001** | 0.40 | **<0.0001** | 0.17 | 0.2436 |
| 0.25 | 37.53 | 0.21 | **0.0125** | -0.12 | 0.1601 | 0.55 | **<0.0001** | 0.41 | **<0.0001** | 0.16 | 0.3433 |
| 0.30 | 28.22 | 0.33 | **0.0007** | -0.27 | **0.0057** | 0.57 | **<0.0001** | 0.51 | **<0.0001** | 0.15 | 0.4504 |
| 0.35 | 21.64 | 0.28 | **0.0138** | -0.24 | **0.0348** | 0.58 | **<0.0001** | 0.47 | **<0.0001** | -0.15 | 0.5217 |
| 0.40 | 15.07 | 0.23 | 0.0952 | -0.34 | **0.0104** | 0.67 | **<0.0001** | 0.66 | **<0.0001** | -0.30 | 0.2767 |
| 0.45 | 12.88 | --- | --- | --- | --- | --- | --- | --- | --- | --- | --- |
| 0.50 | 10.68 | --- | --- | --- | --- | --- | --- | --- | --- | --- | --- |
| 0.55 | 9.59 | --- | --- | --- | --- | --- | --- | --- | --- | --- | --- |
| 0.60 | 6.58 | --- | --- | --- | --- | --- | --- | --- | --- | --- | --- |
| 0.65 | 5.75 | --- | --- | --- | --- | --- | --- | --- | --- | --- | --- |
| 0.70 | 4.93 | --- | --- | --- | --- | --- | --- | --- | --- | --- | --- |
| 0.75 | 3.01 | --- | --- | --- | --- | --- | --- | --- | --- | --- | --- |
| 0.80 | 1.37 | --- | --- | --- | --- | --- | --- | --- | --- | --- | --- |
| 0.85 | 1.10 | --- | --- | --- | --- | --- | --- | --- | --- | --- | --- |
| 0.90 | 0.82 | --- | --- | --- | --- | --- | --- | --- | --- | --- | --- |
| 0.95 | 0.27 | --- | --- | --- | --- | --- | --- | --- | --- | --- | --- |
| 1.00 | 0 | --- | --- | --- | --- | --- | --- | --- | --- | --- | --- |

Bolded p values are those reaching statistical significance, p < 0.05. *Grey rows represent the PAx range where cerebrovascular reactivity is generally considered to be intact.*

*AMP = pulse amplitude of ICP, CPP = cerebral perfusion pressure, ICP = intracranial pressure, iICP.ci = individualized intracranial pressure thresholds with confidence intervals less than 0.2 a.u., MAP = mean arterial pressure, mmHg = millimeters of mercury, PAx = pulse amplitude index (correlation between AMP and MAP), PbtO2 = brain tissue oxygen tension, PRx = pressure reactivity index (correlation between ICP and MAP), RAC = correlation (R) between slow waves of AMP (A) and CPP (C).*

Supplemental Appendix M. Spearman rank correlation analysis between iICP.ci derived using various RAC thresholds and measures of cerebral physiologic insult burden

| Threshold | % Yield | % time with CPP < 60mmHg | | % time with CPP > 70mmHg | | % time with PRx > 0.25 | | % time with PAx > 0.25 | | % time with PbtO_2_ < 20mmHg | |
| --- | --- | --- | --- | --- | --- | --- | --- | --- | --- | --- | --- |
|  |  | Coefficient | p value | Coefficient | p value | Coefficient | p value | Coefficient | p value | Coefficient | p value |
| ICP > 20mmHg | --- | 0.25 | **<0.0001** | -0.02 | 0.7182 | 0.14 | **0.0066** | -0.14 | **0.0068** | -0.09 | 0.3384 |
| -1.00 | 0 | --- | --- | --- | --- | --- | --- | --- | --- | --- | --- |
| -0.95 | 0 | --- | --- | --- | --- | --- | --- | --- | --- | --- | --- |
| -0.90 | 0 | --- | --- | --- | --- | --- | --- | --- | --- | --- | --- |
| -0.85 | 0.55 | --- | --- | --- | --- | --- | --- | --- | --- | --- | --- |
| -0.80 | 1.37 | --- | --- | --- | --- | --- | --- | --- | --- | --- | --- |
| -0.75 | 4.66 | --- | --- | --- | --- | --- | --- | --- | --- | --- | --- |
| -0.70 | 7.95 | --- | --- | --- | --- | --- | --- | --- | --- | --- | --- |
| -0.65 | 10.96 | --- | --- | --- | --- | --- | --- | --- | --- | --- | --- |
| -0.60 | 16.44 | 0.03 | 0.8374 | 0.09 | 0.5108 | -0.06 | 0.6476 | 0.11 | 0.4174 | -0.29 | 0.1834 |
| -0.55 | 19.45 | 0.07 | 0.5839 | 0.10 | 0.4233 | -0.07 | 0.5689 | 0.08 | 0.4905 | -0.17 | 0.4095 |
| -0.50 | 23.84 | 0.20 | 0.0643 | -0.02 | 0.8477 | 0.09 | 0.3840 | 0.18 | 0.0863 | 0.01 | 0.9451 |
| -0.45 | 25.48 | 0.41 | **0.0001** | -0.28 | **0.0067** | 0.16 | 0.1316 | 0.31 | **0.0024** | -0.12 | 0.5192 |
| -0.40 | 28.22 | 0.35 | **0.0003** | -0.31 | **0.0015** | 0.27 | **0.0055** | 0.35 | **0.0003** | -0.05 | 0.7847 |
| -0.35 | 32.05 | 0.17 | 0.0735 | -0.14 | 0.1237 | 0.27 | **0.0028** | 0.30 | **0.0013** | 0.03 | 0.8767 |
| -0.30 | 30.96 | 0.14 | 0.1259 | -0.11 | 0.2629 | 0.30 | **0.0011** | 0.45 | **<0.0001** | -0.09 | 0.5900 |
| -0.25 | 30.68 | 0.17 | 0.0748 | -0.15 | 0.1169 | 0.33 | **0.0004** | 0.41 | **<0.0001** | -0.11 | 0.5204 |
| -0.20 | 28.49 | 0.20 | **0.0410** | -0.22 | **0.0272** | 0.34 | **0.0004** | 0.41 | **<0.0001** | -0.02 | 0.8867 |
| -0.15 | 26.85 | 0.30 | **0.0025** | -0.25 | **0.0141** | 0.34 | **0.0006** | 0.43 | **<0.0001** | 0.10 | 0.5768 |
| -0.10 | 25.21 | 0.36 | **0.0004** | -0.33 | **0.0015** | 0.46 | **<0.0001** | 0.45 | **<0.0001** | -0.03 | 0.8630 |
| -0.05 | 23.56 | 0.22 | **0.0408** | -0.24 | **0.0259** | 0.56 | **<0.0001** | 0.58 | **<0.0001** | -0.05 | 0.8280 |
| 0.00 | 20.27 | 0.16 | 0.1662 | -0.17 | 0.1600 | 0.55 | **<0.0001** | 0.53 | **<0.0001** | -0.19 | 0.3957 |
| 0.05 | 15.89 | 0.11 | 0.3894 | -0.15 | 0.2712 | 0.65 | **<0.0001** | 0.68 | **<0.0001** | 0.06 | 0.8315 |
| 0.10 | 12.60 | --- | --- | --- | --- | --- | --- | --- | --- | --- | --- |
| 0.15 | 10.68 | --- | --- | --- | --- | --- | --- | --- | --- | --- | --- |
| 0.20 | 8.77 | --- | --- | --- | --- | --- | --- | --- | --- | --- | --- |
| 0.25 | 6.85 | --- | --- | --- | --- | --- | --- | --- | --- | --- | --- |
| 0.30 | 6.03 | --- | --- | --- | --- | --- | --- | --- | --- | --- | --- |
| 0.35 | 4.93 | --- | --- | --- | --- | --- | --- | --- | --- | --- | --- |
| 0.40 | 3.29 | --- | --- | --- | --- | --- | --- | --- | --- | --- | --- |
| 0.45 | 3.01 | --- | --- | --- | --- | --- | --- | --- | --- | --- | --- |
| 0.50 | 2.74 | --- | --- | --- | --- | --- | --- | --- | --- | --- | --- |
| 0.55 | 2.47 | --- | --- | --- | --- | --- | --- | --- | --- | --- | --- |
| 0.60 | 2.19 | --- | --- | --- | --- | --- | --- | --- | --- | --- | --- |
| 0.65 | 2.19 | --- | --- | --- | --- | --- | --- | --- | --- | --- | --- |
| 0.70 | 1.10 | --- | --- | --- | --- | --- | --- | --- | --- | --- | --- |
| 0.75 | 0.55 | --- | --- | --- | --- | --- | --- | --- | --- | --- | --- |
| 0.80 | 0.27 | --- | --- | --- | --- | --- | --- | --- | --- | --- | --- |
| 0.85 | 0.27 | --- | --- | --- | --- | --- | --- | --- | --- | --- | --- |
| 0.90 | 0.27 | --- | --- | --- | --- | --- | --- | --- | --- | --- | --- |
| 0.95 | 0 | --- | --- | --- | --- | --- | --- | --- | --- | --- | --- |
| 1.00 | 0 | --- | --- | --- | --- | --- | --- | --- | --- | --- | --- |

Bolded p values are those reaching statistical significance, p < 0.05. *Grey rows represent the RAC range where cerebrovascular reactivity is generally considered to be intact.*

*AMP = pulse amplitude of ICP, CPP = cerebral perfusion pressure, ICP = intracranial pressure, iICP.ci = individualized intracranial pressure thresholds with confidence intervals less than 0.2 a.u., MAP = mean arterial pressure, mmHg = millimeters of mercury, PAx = pulse amplitude index (correlation between AMP and MAP), PbtO2 = brain tissue oxygen tension, PRx = pressure reactivity index (correlation between ICP and MAP), RAC = correlation (R) between slow waves of AMP (A) and CPP (C).*

Supplemental Appendix N. Chi-square outcome analysis of iICP derived using various PRx thresholds dichotomized by age

| **Threshold** | **Age < 40 years (n = 189)** | | | | | **Age ≥ 40 years (n = 174)** | | | | |
| --- | --- | --- | --- | --- | --- | --- | --- | --- | --- | --- |
|  | **Yield** | **Alive vs Dead** | | **Favorable vs Unfavorable** | | **Yield** | **Alive vs Dead** | | **Favorable vs Unfavorable** | |
|  |  | **χ^2^** | **p value** | **χ^2^** | **p value** |  | **χ^2^** | **p value** | **χ^2^** | **p value** |
| **-1.00** | 0 | --- | --- | --- | --- | 0 | --- | --- | --- | --- |
| **-0.95** | 0 | --- | --- | --- | --- | 0 | --- | --- | --- | --- |
| **-0.90** | 0 | --- | --- | --- | --- | 0 | --- | --- | --- | --- |
| **-0.85** | 0 | --- | --- | --- | --- | 0 | --- | --- | --- | --- |
| **-0.80** | 0 | --- | --- | --- | --- | 0 | --- | --- | --- | --- |
| **-0.75** | 0 | --- | --- | --- | --- | 1.14 | --- | --- | --- | --- |
| **-0.70** | 1.05 | --- | --- | --- | --- | 1.14 | --- | --- | --- | --- |
| **-0.65** | 1.57 | --- | --- | --- | --- | 1.14 | --- | --- | --- | --- |
| **-0.60** | 2.62 | --- | --- | --- | --- | 1.70 | --- | --- | --- | --- |
| **-0.55** | 2.62 | --- | --- | --- | --- | 2.27 | --- | --- | --- | --- |
| **-0.50** | 4.19 | --- | --- | --- | --- | 2.84 | --- | --- | --- | --- |
| **-0.45** | 4.71 | --- | --- | --- | --- | 4.55 | --- | --- | --- | --- |
| **-0.40** | 6.81 | --- | --- | --- | --- | 5.68 | --- | --- | --- | --- |
| **-0.35** | 7.33 | --- | --- | --- | --- | 6.82 | --- | --- | --- | --- |
| **-0.30** | 9.42 | --- | --- | --- | --- | 9.66 | --- | --- | --- | --- |
| **-0.25** | 12.04 | --- | --- | --- | --- | 14.20 | --- | --- | --- | --- |
| **-0.20** | 19.37 | 0.00 | 1.0000 | 0.17 | 0.6785 | 18.18 | 0.00 | 1.0000 | 0.06 | 0.8100 |
| **-0.15** | 25.65 | 0.38 | 0.5390 | 0.00 | 0.9769 | 23.30 | 0.00 | 1.0000 | 0.00 | 1.0000 |
| **-0.10** | 36.13 | 0.32 | 0.5714 | 0.73 | 0.3924 | 30.11 | 0.00 | 1.0000 | 0.01 | 0.9075 |
| **-0.05** | 43.46 | 0.60 | 0.4401 | 1.81 | 0.1784 | 42.05 | 0.04 | 0.8394 | 1.30 | 0.2542 |
| **0.00** | 54.45 | 0.63 | 0.4262 | 0.75 | 0.3854 | 51.70 | 0.00 | 1.0000 | 0.60 | 0.4368 |
| **0.05** | 62.83 | 5.00 | **0.0253** | 7.06 | **0.0079** | 58.52 | 1.59 | 0.2076 | 1.87 | 0.1714 |
| **0.10** | 67.54 | 1.84 | 0.1752 | 5.86 | **0.0155** | 61.36 | 0.72 | 0.3958 | 1.83 | 0.1757 |
| **0.15** | 70.16 | 5.47 | **0.0193** | 4.51 | **0.0336** | 68.18 | 0.01 | 0.9384 | 0.16 | 0.6924 |
| **0.20** | 71.20 | 3.28 | 0.0702 | 5.26 | **0.0218** | 72.16 | 0.37 | 0.5443 | 1.02 | 0.3116 |
| **0.25** | 73.30 | 0.83 | 0.3627 | 2.81 | 0.0939 | 72.73 | 1.11 | 0.2928 | 1.30 | 0.2541 |
| **0.30** | 72.77 | 0.68 | 0.4090 | 1.36 | 0.2428 | 69.32 | 0.00 | 0.9454 | 0.09 | 0.7658 |
| **0.35** | 72.77 | 0.78 | 0.3777 | 0.74 | 0.3911 | 67.05 | 0.00 | 1.0000 | 0.00 | 0.9713 |
| **0.40** | 67.54 | 0.35 | 0.5569 | 1.16 | 0.2811 | 66.48 | 0.00 | 1.0000 | 0.00 | 1.0000 |
| **0.45** | 62.83 | 0.02 | 0.8890 | 0.56 | 0.4550 | 63.07 | 0.00 | 1.0000 | 0.00 | 1.0000 |
| **0.50** | 58.12 | 0.00 | 0.9480 | 1.50 | 0.2214 | 59.66 | 0.31 | 0.5807 | 1.38 | 0.2394 |
| **0.55** | 55.50 | 0.00 | 1.0000 | 0.07 | 0.7953 | 56.82 | 1.61 | 0.2040 | 0.81 | 0.3673 |
| **0.60** | 49.74 | 0.60 | 0.4386 | 0.07 | 0.7899 | 50.00 | 1.54 | 0.2139 | 0.75 | 0.3872 |
| **0.65** | 45.03 | 1.79 | 0.1803 | 0.61 | 0.4336 | 46.59 | 0.66 | 0.4156 | 0.23 | 0.6345 |
| **0.70** | 41.36 | 0.53 | 0.4668 | 0.05 | 0.8211 | 41.48 | 0.65 | 0.4216 | 0.19 | 0.6661 |
| **0.75** | 36.13 | 0.42 | 0.5148 | 0.04 | 0.8374 | 38.64 | 0.48 | 0.4865 | 0.15 | 0.6983 |
| **0.80** | 32.98 | 5.79 | **0.0162** | 0.29 | 0.5930 | 34.09 | 0.00 | 1.0000 | 0.00 | 1.0000 |
| **0.85** | 27.23 | 6.42 | **0.0113** | 0.56 | 0.4561 | 29.55 | 0.00 | 1.0000 | 4.08 | 0.0433 |
| **0.90** | 21.47 | 4.24 | **0.0396** | 0.47 | 0.4927 | 23.30 | 0.03 | 0.8694 | 4.57 | 0.0326 |
| **0.95** | 16.23 | 3.57 | 0.0588 | 1.29 | 0.2568 | 17.61 | 0.00 | 1.0000 | 2.29 | 0.1306 |
| **1.00** | 13.09 | --- | --- | --- | --- | 13.07 | --- | --- | --- | --- |

*Bolded p values are those reaching statistical significance, p < 0.05. Grey rows represent the PRx range where cerebrovascular reactivity is generally considered to be intact.*

*ICP = intracranial pressure, iICP = individualized intracranial pressure thresholds, MAP = mean arterial pressure, PRx = pressure reactivity index (correlation between ICP and MAP), χ^2^ = chi-square value.*

Supplemental Appendix O. Chi-square outcome analysis of iICP derived using various PAx thresholds dichotomized by age

| **Threshold** | **Age < 40 years (n = 189)** | | | | | **Age ≥ 40 years (n = 174)** | | | | |
| --- | --- | --- | --- | --- | --- | --- | --- | --- | --- | --- |
|  | **Yield** | **Alive vs Dead** | | **Favorable vs Unfavorable** | | **Yield** | **Alive vs Dead** | | **Favorable vs Unfavorable** | |
|  |  | **χ^2^** | **p value** | **χ^2^** | **p value** |  | **χ^2^** | **p value** | **χ^2^** | **p value** |
| **-1.00** | 0 | --- | --- | --- | --- | 0 | --- | --- | --- | --- |
| **-0.95** | 0 | --- | --- | --- | --- | 0 | --- | --- | --- | --- |
| **-0.90** | 0 | --- | --- | --- | --- | 0 | --- | --- | --- | --- |
| **-0.85** | 0 | --- | --- | --- | --- | 0 | --- | --- | --- | --- |
| **-0.80** | 0 | --- | --- | --- | --- | 0.57 | --- | --- | --- | --- |
| **-0.75** | 0.52 | --- | --- | --- | --- | 0.57 | --- | --- | --- | --- |
| **-0.70** | 0.52 | --- | --- | --- | --- | 0.57 | --- | --- | --- | --- |
| **-0.65** | 1.05 | --- | --- | --- | --- | 0.57 | --- | --- | --- | --- |
| **-0.60** | 1.05 | --- | --- | --- | --- | 1.14 | --- | --- | --- | --- |
| **-0.55** | 1.57 | --- | --- | --- | --- | 2.27 | --- | --- | --- | --- |
| **-0.50** | 4.19 | --- | --- | --- | --- | 3.98 | --- | --- | --- | --- |
| **-0.45** | 9.95 | --- | --- | --- | --- | 5.11 | --- | --- | --- | --- |
| **-0.40** | 12.57 | --- | --- | --- | --- | 6.25 | --- | --- | --- | --- |
| **-0.35** | 18.32 | 0.00 | 1 | 0.00 | 1 | 9.66 | --- | --- | --- | --- |
| **-0.30** | 26.18 | 0.00 | 1 | 0.00 | 1 | 11.93 | --- | --- | --- | --- |
| **-0.25** | 35.08 | 0.45 | 0.5035 | 0.22 | 0.6401 | 16.48 | 1.92 | 0.1659 | 1.47 | 0.2256 |
| **-0.20** | 43.46 | 6.68 | **0.0098** | 9.26 | **0.0023** | 25.57 | 1.23 | 0.2667 | 0.65 | 0.4216 |
| **-0.15** | 52.36 | 2.65 | 0.1035 | 1.72 | 0.1891 | 37.5 | 0.33 | 0.5684 | 1.08 | 0.2991 |
| **-0.10** | 63.35 | 0.47 | 0.4911 | 3.52 | 0.0607 | 43.18 | 0.00 | 0.9546 | 0.00 | 0.9666 |
| **-0.05** | 67.54 | 0.40 | 0.5257 | 3.06 | 0.0805 | 53.98 | 0.04 | 0.8405 | 0.00 | 1.0000 |
| **0.00** | 72.77 | 1.55 | 0.2135 | 4.75 | **0.0292** | 59.66 | 0.00 | 0.9835 | 0.06 | 0.8073 |
| **0.05** | 71.73 | 0.72 | 0.3966 | 2.31 | 0.1287 | 62.5 | 0.06 | 0.8027 | 0.00 | 1.0000 |
| **0.10** | 65.97 | 1.96 | 0.1613 | 1.90 | 0.1680 | 62.5 | 0.38 | 0.5388 | 0.00 | 1.0000 |
| **0.15** | 62.3 | 2.48 | 0.1149 | 1.03 | 0.3099 | 61.36 | 0.00 | 1.0000 | 0.02 | 0.8825 |
| **0.20** | 58.64 | 3.88 | **0.0490** | 2.56 | 0.1094 | 59.09 | 0.00 | 1.0000 | 0.00 | 1.0000 |
| **0.25** | 54.97 | 5.59 | **0.0180** | 3.49 | 0.0618 | 56.25 | 0.00 | 1.0000 | 0.00 | 1.0000 |
| **0.30** | 49.74 | 1.81 | 0.1790 | 1.29 | 0.2556 | 53.41 | 0.17 | 0.6767 | 0.20 | 0.6578 |
| **0.35** | 44.5 | 1.88 | 0.1708 | 1.41 | 0.2343 | 47.73 | 1.44 | 0.2295 | 0.11 | 0.7421 |
| **0.40** | 36.13 | 0.89 | 0.3462 | 0.09 | 0.7692 | 43.18 | 1.73 | 0.1883 | 0.32 | 0.5715 |
| **0.45** | 31.94 | 1.82 | 0.1773 | 0.56 | 0.4559 | 35.8 | 3.58 | 0.0585 | 1.73 | 0.1878 |
| **0.50** | 27.75 | 0.40 | 0.5271 | 0.02 | 0.8811 | 32.95 | 2.39 | 0.1224 | 1.12 | 0.2896 |
| **0.55** | 25.65 | 3.20 | 0.0735 | 1.53 | 0.2167 | 25.57 | 2.04 | 0.1529 | 0.82 | 0.3652 |
| **0.60** | 21.99 | 3.33 | 0.0680 | 1.67 | 0.1963 | 22.16 | 0.59 | 0.4412 | 0.20 | 0.6556 |
| **0.65** | 17.28 | 0.15 | 0.6996 | 0.01 | 0.9213 | 18.75 | 0.40 | 0.5249 | 0.07 | 0.7958 |
| **0.70** | 13.61 | --- | --- | --- | --- | 15.34 | 0.32 | 0.5725 | 0.01 | 0.9215 |
| **0.75** | 11.52 | --- | --- | --- | --- | 11.36 | --- | --- | --- | --- |
| **0.80** | 9.95 | --- | --- | --- | --- | 7.95 | --- | --- | --- | --- |
| **0.85** | 8.9 | --- | --- | --- | --- | 6.25 | --- | --- | --- | --- |
| **0.90** | 7.33 | --- | --- | --- | --- | 4.55 | --- | --- | --- | --- |
| **0.95** | 5.24 | --- | --- | --- | --- | 3.41 | --- | --- | --- | --- |
| **1.00** | 4.19 | --- | --- | --- | --- | 1.7 | --- | --- | --- | --- |

*Bolded p values are those reaching statistical significance, p < 0.05. Grey rows represent the PAx range where cerebrovascular reactivity is generally considered to be intact.*

*ICP = intracranial pressure, iICP = individualized intracranial pressure thresholds, MAP = mean arterial pressure, PAx = pulse amplitude index (correlation between AMP and MAP), χ^2^ = chi-square value.*

Supplemental Appendix P. Chi-square outcome analysis of iICP derived using various RAC thresholds dichotomized by age

| **Threshold** | **Age < 40 years (n = 189)** | | | | | **Age ≥ 40 years (n = 174)** | | | | |
| --- | --- | --- | --- | --- | --- | --- | --- | --- | --- | --- |
|  | **Yield** | **Alive vs Dead** | | **Favorable vs Unfavorable** | | **Yield** | **Alive vs Dead** | | **Favorable vs Unfavorable** | |
|  |  | **χ^2^** | **p value** | **χ^2^** | **p value** |  | **χ^2^** | **p value** | **χ^2^** | **p value** |
| **-1.00** | 0.52 | --- | --- | --- | --- | 0.57 | --- | --- | --- | --- |
| **-0.95** | 0.52 | --- | --- | --- | --- | 1.14 | --- | --- | --- | --- |
| **-0.90** | 0.52 | --- | --- | --- | --- | 1.14 | --- | --- | --- | --- |
| **-0.85** | 1.05 | --- | --- | --- | --- | 1.14 | --- | --- | --- | --- |
| **-0.80** | 1.57 | --- | --- | --- | --- | 3.98 | --- | --- | --- | --- |
| **-0.75** | 7.33 | --- | --- | --- | --- | 5.68 | --- | --- | --- | --- |
| **-0.70** | 12.57 | --- | --- | --- | --- | 8.52 | --- | --- | --- | --- |
| **-0.65** | 18.32 | 0.12 | 0.7251 | 0.00 | 1.0000 | 10.8 | --- | --- | --- | --- |
| **-0.60** | 25.65 | 0.15 | 0.6965 | 0.00 | 1.0000 | 15.91 | 0.00 | 1.0000 | 0.00 | 1.0000 |
| **-0.55** | 31.94 | 0.00 | 1.0000 | 0.00 | 0.9517 | 18.75 | 0.19 | 0.6670 | 0.00 | 1.0000 |
| **-0.50** | 36.13 | 0.95 | 0.3297 | 0.00 | 1.0000 | 26.7 | 1.39 | 0.2390 | 0.61 | 0.4337 |
| **-0.45** | 39.79 | 7.60 | **0.0058** | 1.96 | 0.1611 | 28.41 | 3.84 | 0.0500 | 1.55 | 0.2135 |
| **-0.40** | 41.88 | 2.37 | 0.1239 | 1.16 | 0.2823 | 30.11 | 2.18 | 0.1398 | 0.16 | 0.6935 |
| **-0.35** | 46.07 | 0.16 | 0.6852 | 0.01 | 0.9316 | 36.93 | 1.07 | 0.3001 | 0.06 | 0.8009 |
| **-0.30** | 43.98 | 2.95 | 0.0858 | 0.03 | 0.8528 | 44.32 | 2.38 | 0.1227 | 0.85 | 0.3562 |
| **-0.25** | 45.55 | 2.52 | 0.1121 | 1.48 | 0.2243 | 43.75 | 1.59 | 0.2072 | 0.71 | 0.3983 |
| **-0.20** | 44.5 | 0.25 | 0.6168 | 0.69 | 0.4073 | 40.34 | 0.30 | 0.5859 | 0.18 | 0.6674 |
| **-0.15** | 43.46 | 0.00 | 1.0000 | 0.38 | 0.5375 | 40.34 | 0.00 | 1.0000 | 0.00 | 1.0000 |
| **-0.10** | 40.84 | 0.00 | 1.0000 | 1.83 | 0.1759 | 42.05 | 0.00 | 1.0000 | 0.00 | 0.9670 |
| **-0.05** | 39.27 | 0.02 | 0.8777 | 0.15 | 0.6987 | 39.77 | 0.00 | 0.9677 | 0.23 | 0.6336 |
| **0.00** | 36.13 | 0.17 | 0.6777 | 0.45 | 0.5038 | 37.5 | 0.00 | 1.0000 | 0.00 | 1.0000 |
| **0.05** | 34.03 | 0.00 | 1.0000 | 0.00 | 1.0000 | 35.23 | 0.00 | 1.0000 | 0.00 | 1.0000 |
| **0.10** | 28.8 | 0.00 | 1.0000 | 0.00 | 1.0000 | 31.25 | 0.19 | 0.6642 | 0.00 | 1.0000 |
| **0.15** | 26.7 | 1.48 | 0.2244 | 1.45 | 0.2293 | 26.7 | 0.10 | 0.7491 | 0.00 | 1.0000 |
| **0.20** | 21.99 | 1.10 | 0.2940 | 1.29 | 0.2565 | 22.16 | 0.05 | 0.8183 | 0.00 | 1.0000 |
| **0.25** | 18.85 | 1.06 | 0.3042 | 0.17 | 0.6795 | 18.75 | 0.28 | 0.5984 | 0.00 | 1.0000 |
| **0.30** | 15.71 | 0.69 | 0.4060 | 0.16 | 0.6930 | 17.61 | 1.45 | 0.2278 | 0.47 | 0.4913 |
| **0.35** | 13.61 | --- | --- | --- | --- | 15.34 | 0.91 | 0.3392 | 0.32 | 0.5736 |
| **0.40** | 10.99 | --- | --- | --- | --- | 11.93 | --- | --- | --- | --- |
| **0.45** | 7.85 | --- | --- | --- | --- | 10.8 | --- | --- | --- | --- |
| **0.50** | 7.85 | --- | --- | --- | --- | 10.8 | --- | --- | --- | --- |
| **0.55** | 7.33 | --- | --- | --- | --- | 10.23 | --- | --- | --- | --- |
| **0.60** | 7.33 | --- | --- | --- | --- | 9.09 | --- | --- | --- | --- |
| **0.65** | 6.81 | --- | --- | --- | --- | 7.95 | --- | --- | --- | --- |
| **0.70** | 5.76 | --- | --- | --- | --- | 5.68 | --- | --- | --- | --- |
| **0.75** | 3.66 | --- | --- | --- | --- | 3.98 | --- | --- | --- | --- |
| **0.80** | 2.09 | --- | --- | --- | --- | 0.57 | --- | --- | --- | --- |
| **0.85** | 2.09 | --- | --- | --- | --- | 0.57 | --- | --- | --- | --- |
| **0.90** | 2.09 | --- | --- | --- | --- | 0.57 | --- | --- | --- | --- |
| **0.95** | 2.09 | --- | --- | --- | --- | 0 | --- | --- | --- | --- |
| **1.00** | 1.57 | --- | --- | --- | --- | 0 | --- | --- | --- | --- |

*Bolded p values are those reaching statistical significance, p < 0.05. Grey rows represent the RAC range where cerebrovascular reactivity is generally considered to be intact.*

*ICP = intracranial pressure, iICP = individualized intracranial pressure thresholds, MAP = mean arterial pressure, RAC = correlation (R) between slow waves of AMP (A) and CPP (C), χ^2^ = chi-square value.*

Supplemental Appendix Q. Chi-square outcome analysis of iICP derived using various PRx thresholds dichotomized by sex

| **Threshold** | **Male (n = 283)** | | | | | **Female (n = 81)** | | | | |
| --- | --- | --- | --- | --- | --- | --- | --- | --- | --- | --- |
|  | **Yield** | **Alive vs Dead** | | **Favorable vs Unfavorable** | | **Yield** | **Alive vs Dead** | | **Favorable vs Unfavorable** | |
|  |  | **χ^2^** | **p value** | **χ^2^** | **p value** |  | **χ^2^** | **p value** | **χ^2^** | **p value** |
| **-1.00** | 0.35 | --- | --- | --- | --- | 0 | --- | --- | --- | --- |
| **-0.95** | 0.35 | --- | --- | --- | --- | 0 | --- | --- | --- | --- |
| **-0.90** | 0.35 | --- | --- | --- | --- | 0 | --- | --- | --- | --- |
| **-0.85** | 0.35 | --- | --- | --- | --- | 0 | --- | --- | --- | --- |
| **-0.80** | 0.35 | --- | --- | --- | --- | 0 | --- | --- | --- | --- |
| **-0.75** | 0.70 | --- | --- | --- | --- | 0 | --- | --- | --- | --- |
| **-0.70** | 1.06 | --- | --- | --- | --- | 1.22 | --- | --- | --- | --- |
| **-0.65** | 1.41 | --- | --- | --- | --- | 1.22 | --- | --- | --- | --- |
| **-0.60** | 2.46 | --- | --- | --- | --- | 1.22 | --- | --- | --- | --- |
| **-0.55** | 2.82 | --- | --- | --- | --- | 1.22 | --- | --- | --- | --- |
| **-0.50** | 3.87 | --- | --- | --- | --- | 2.44 | --- | --- | --- | --- |
| **-0.45** | 5.28 | --- | --- | --- | --- | 2.44 | --- | --- | --- | --- |
| **-0.40** | 7.39 | --- | --- | --- | --- | 2.44 | --- | --- | --- | --- |
| **-0.35** | 8.45 | --- | --- | --- | --- | 2.44 | --- | --- | --- | --- |
| **-0.30** | 10.92 | --- | --- | --- | --- | 4.88 | --- | --- | --- | --- |
| **-0.25** | 14.44 | --- | --- | --- | --- | 8.54 | --- | --- | --- | --- |
| **-0.20** | 20.07 | 0.36 | 0.5489 | 0 | 1 | 14.63 | --- | --- | --- | --- |
| **-0.15** | 25.35 | 0.97 | 0.3243 | 0.26 | 0.6085 | 21.95 | 0.16 | 0.6926 | 0.1 | 0.7513 |
| **-0.10** | 32.39 | 2.3 | 0.1295 | 1.3 | 0.255 | 37.8 | 1.13 | 0.2872 | 0 | 1 |
| **-0.05** | 42.61 | 2.35 | 0.1257 | 4.6 | 0.032 | 45.12 | 0.06 | 0.8013 | 0.01 | 0.9232 |
| **0.00** | 53.17 | 0.48 | 0.4886 | 0.79 | 0.3754 | 54.88 | 0.13 | 0.715 | 1.64 | 0.2008 |
| **0.05** | 60.92 | 5.18 | **0.0228** | 6.18 | **0.0129** | 62.2 | 0.16 | 0.6895 | 0.93 | 0.3343 |
| **0.10** | 63.38 | 3.19 | 0.0743 | 7.26 | **0.0071** | 70.73 | 0.01 | 0.9409 | 0.53 | 0.468 |
| **0.15** | 68.66 | 3.04 | 0.0812 | 3.13 | 0.0767 | 73.17 | 0.05 | 0.8211 | 0.26 | 0.6104 |
| **0.20** | 73.24 | 2.38 | 0.1232 | 3.63 | 0.0567 | 68.29 | 0.02 | 0.8932 | 1.3 | 0.2534 |
| **0.25** | 73.94 | 2.17 | 0.1407 | 2.87 | 0.09 | 71.95 | 0.27 | 0.6017 | 1.57 | 0.2099 |
| **0.30** | 72.18 | 0.48 | 0.4876 | 1.28 | 0.2576 | 69.51 | 0 | 1 | 0.01 | 0.9279 |
| **0.35** | 71.48 | 0.81 | 0.3667 | 0.69 | 0.4051 | 67.07 | 0 | 1 | 0 | 1 |
| **0.40** | 68.66 | 0.04 | 0.8336 | 0.69 | 0.4055 | 63.41 | 0 | 1 | 0 | 1 |
| **0.45** | 64.44 | 0 | 1 | 0.2 | 0.6588 | 59.76 | 0 | 1 | 0 | 1 |
| **0.50** | 59.86 | 0.03 | 0.8722 | 2.78 | 0.0953 | 57.32 | --- | --- | --- | --- |
| **0.55** | 57.04 | 0.82 | 0.3648 | 1.4 | 0.2367 | 54.88 | --- | --- | --- | --- |
| **0.60** | 50.70 | 0.92 | 0.3368 | 0.2 | 0.6553 | 48.78 | --- | --- | --- | --- |
| **0.65** | 45.42 | 0.93 | 0.3341 | 0.21 | 0.6479 | 48.78 | --- | --- | --- | --- |
| **0.70** | 40.49 | 0.96 | 0.3269 | 0.17 | 0.6795 | 46.34 | --- | --- | --- | --- |
| **0.75** | 35.92 | 0.69 | 0.4053 | 0.14 | 0.7049 | 43.9 | --- | --- | --- | --- |
| **0.80** | 32.39 | --- | --- | --- | --- | 39.02 | --- | --- | --- | --- |
| **0.85** | 26.76 | --- | --- | --- | --- | 35.37 | --- | --- | --- | --- |
| **0.90** | 20.07 | --- | --- | --- | --- | 30.49 | --- | --- | --- | --- |
| **0.95** | 14.79 | --- | --- | --- | --- | 24.39 | --- | --- | --- | --- |
| **1.00** | 11.62 | --- | --- | --- | --- | 18.29 | --- | --- | --- | --- |

*Bolded p values are those reaching statistical significance, p < 0.05. Grey rows represent the PRx range where cerebrovascular reactivity is generally considered to be intact.*

*ICP = intracranial pressure, iICP = individualized intracranial pressure thresholds, MAP = mean arterial pressure, PRx = pressure reactivity index (correlation between ICP and MAP), χ^2^ = chi-square value.*

Supplemental Appendix R. Chi-square outcome analysis of iICP derived using various PAx thresholds dichotomized by sex

| **Threshold** | **Male (n = 283)** | | | | | **Female (n = 81)** | | | | |
| --- | --- | --- | --- | --- | --- | --- | --- | --- | --- | --- |
|  | **Yield** | **Alive vs Dead** | | **Favorable vs Unfavorable** | | **Yield** | **Alive vs Dead** | | **Favorable vs Unfavorable** | |
|  |  | **χ^2^** | **p value** | **χ^2^** | **p value** |  | **χ^2^** | **p value** | **χ^2^** | **p value** |
| **-1.00** | 0 | --- | --- | --- | --- | 0 | --- | --- | --- | --- |
| **-0.95** | 0 | --- | --- | --- | --- | 0 | --- | --- | --- | --- |
| **-0.90** | 0 | --- | --- | --- | --- | 0 | --- | --- | --- | --- |
| **-0.85** | 0 | --- | --- | --- | --- | 0 | --- | --- | --- | --- |
| **-0.80** | 0.35 | --- | --- | --- | --- | 0 | --- | --- | --- | --- |
| **-0.75** | 0.35 | --- | --- | --- | --- | 1.22 | --- | --- | --- | --- |
| **-0.70** | 0.35 | --- | --- | --- | --- | 1.22 | --- | --- | --- | --- |
| **-0.65** | 0.7 | --- | --- | --- | --- | 1.22 | --- | --- | --- | --- |
| **-0.60** | 1.06 | --- | --- | --- | --- | 1.22 | --- | --- | --- | --- |
| **-0.55** | 1.76 | --- | --- | --- | --- | 2.44 | --- | --- | --- | --- |
| **-0.50** | 4.58 | --- | --- | --- | --- | 2.44 | --- | --- | --- | --- |
| **-0.45** | 7.75 | --- | --- | --- | --- | 7.32 | --- | --- | --- | --- |
| **-0.40** | 10.21 | --- | --- | --- | --- | 7.32 | --- | --- | --- | --- |
| **-0.35** | 14.44 | --- | --- | --- | --- | 13.41 | --- | --- | --- | --- |
| **-0.30** | 20.42 | 0.61 | 0.4352 | 0 | 1 | 15.85 | 0 | 1 | 0 | 1 |
| **-0.25** | 27.46 | 4.69 | **0.0303** | 1.22 | 0.2692 | 21.95 | 0.01 | 0.9297 | 0 | 1 |
| **-0.20** | 37.32 | 8.61 | **0.0033** | 8.88 | **0.0029** | 26.83 | 0 | 1 | 0 | 0.9577 |
| **-0.15** | 46.83 | 3.05 | 0.0806 | 3.52 | 0.0606 | 40.24 | 0 | 1 | 0 | 1 |
| **-0.10** | 52.82 | 0.92 | 0.3363 | 3.24 | 0.0718 | 57.32 | 0 | 1 | 0 | 1 |
| **-0.05** | 61.27 | 0.39 | 0.5349 | 1.9 | 0.1681 | 60.98 | 0.04 | 0.8372 | 0 | 1 |
| **0.00** | 66.2 | 0.47 | 0.4917 | 0.91 | 0.34 | 68.29 | 0.76 | 0.3818 | 0.79 | 0.3735 |
| **0.05** | 66.2 | 1.33 | 0.2482 | 1.43 | 0.2315 | 73.17 | 0.22 | 0.6356 | 0.34 | 0.5601 |
| **0.10** | 65.49 | 2.23 | 0.1351 | 0.76 | 0.384 | 62.2 | 0.64 | 0.4237 | 0.65 | 0.421 |
| **0.15** | 63.38 | 1.97 | 0.1607 | 1.83 | 0.1756 | 58.54 | 0.24 | 0.6213 | 0.01 | 0.9231 |
| **0.20** | 60.21 | 1.22 | 0.2696 | 0.89 | 0.345 | 56.1 | 0 | 1 | 0.03 | 0.8679 |
| **0.25** | 56.69 | 1.74 | 0.1871 | 0.54 | 0.4608 | 52.44 | 0.22 | 0.6368 | 0.55 | 0.4583 |
| **0.30** | 51.06 | 0.29 | 0.5879 | 0.07 | 0.7982 | 53.66 | 2.36 | 0.1241 | --- | --- |
| **0.35** | 45.07 | 0.84 | 0.3599 | 0.27 | 0.6062 | 50 | --- | --- | --- | --- |
| **0.40** | 38.38 | 1.09 | 0.297 | 0.06 | 0.8019 | 43.9 | --- | --- | --- | --- |
| **0.45** | 32.04 | 3.56 | 0.0593 | 1.4 | 0.2374 | 40.24 | --- | --- | --- | --- |
| **0.50** | 29.23 | 3.55 | 0.0594 | 1.39 | 0.2392 | 34.15 | --- | --- | --- | --- |
| **0.55** | 23.94 | --- | --- | --- | --- | 31.71 | --- | --- | --- | --- |
| **0.60** | 20.77 | --- | --- | --- | --- | 26.83 | --- | --- | --- | --- |
| **0.65** | 16.55 | --- | --- | --- | --- | 23.17 | --- | --- | --- | --- |
| **0.70** | 12.68 | --- | --- | --- | --- | 20.73 | --- | --- | --- | --- |
| **0.75** | 9.51 | --- | --- | --- | --- | 18.29 | --- | --- | --- | --- |
| **0.80** | 7.04 | --- | --- | --- | --- | 15.85 | --- | --- | --- | --- |
| **0.85** | 5.99 | --- | --- | --- | --- | 13.41 | --- | --- | --- | --- |
| **0.90** | 4.23 | --- | --- | --- | --- | 12.2 | --- | --- | --- | --- |
| **0.95** | 2.82 | --- | --- | --- | --- | 9.76 | --- | --- | --- | --- |
| **1.00** | 1.76 | --- | --- | --- | --- | 7.32 | --- | --- | --- | --- |

*Bolded p values are those reaching statistical significance, p < 0.05. Grey rows represent the PAx range where cerebrovascular reactivity is generally considered to be intact.*

*ICP = intracranial pressure, iICP = individualized intracranial pressure thresholds, MAP = mean arterial pressure, PAx = pulse amplitude index (correlation between AMP and MAP), χ^2^ = chi-square value.*

Supplemental Appendix S. Chi-square outcome analysis of iICP derived using various RAC thresholds dichotomized by sex

| **Threshold** | **Male (n = 283)** | | | | | **Female (n = 81)** | | | | |
| --- | --- | --- | --- | --- | --- | --- | --- | --- | --- | --- |
|  | **Yield** | **Alive vs Dead** | | **Favorable vs Unfavorable** | | **Yield** | **Alive vs Dead** | | **Favorable vs Unfavorable** | |
|  |  | **χ^2^** | **p value** | **χ^2^** | **p value** |  | **χ^2^** | **p value** | **χ^2^** | **p value** |
| **-1.00** | 0 | --- | --- | --- | --- | 2.44 | --- | --- | --- | --- |
| **-0.95** | 0.35 | --- | --- | --- | --- | 2.44 | --- | --- | --- | --- |
| **-0.90** | 0.35 | --- | --- | --- | --- | 2.44 | --- | --- | --- | --- |
| **-0.85** | 0.7 | --- | --- | --- | --- | 2.44 | --- | --- | --- | --- |
| **-0.80** | 2.11 | --- | --- | --- | --- | 4.88 | --- | --- | --- | --- |
| **-0.75** | 5.99 | --- | --- | --- | --- | 8.54 | --- | --- | --- | --- |
| **-0.70** | 9.86 | --- | --- | --- | --- | 13.41 | --- | --- | --- | --- |
| **-0.65** | 13.38 | --- | --- | --- | --- | 19.51 | --- | --- | --- | --- |
| **-0.60** | 20.07 | 0.42 | 0.5155 | 0.1 | 0.7528 | 24.39 | 0 | 1 | 0.19 | 0.6614 |
| **-0.55** | 24.3 | 0 | 1 | 0.38 | 0.5351 | 30.49 | 0 | 1 | 0.05 | 0.8164 |
| **-0.50** | 30.28 | 3.75 | 0.0527 | 1.76 | 0.1851 | 36.59 | 0 | 1 | 0.6 | 0.4376 |
| **-0.45** | 33.45 | 13.47 | **0.0002** | 6.03 | **0.0141** | 37.8 | 0.05 | 0.8153 | 0 | 1 |
| **-0.40** | 36.27 | 3.46 | 0.0628 | 1.41 | 0.2352 | 36.59 | 1.13 | 0.2875 | 0.07 | 0.7919 |
| **-0.35** | 42.61 | 1.09 | 0.2974 | 0.62 | 0.4321 | 39.02 | 0.15 | 0.6994 | 0 | 1 |
| **-0.30** | 45.42 | 4.19 | **0.0408** | 1.14 | 0.2854 | 40.24 | 1.69 | 0.1933 | 0 | 1 |
| **-0.25** | 47.54 | 2.28 | 0.1308 | 1.75 | 0.1856 | 35.37 | 0.72 | 0.3975 | 0 | 1 |
| **-0.20** | 45.77 | 0.11 | 0.7365 | 0.4 | 0.5257 | 31.71 | 1.02 | 0.3135 | 0.35 | 0.5551 |
| **-0.15** | 45.07 | 0 | 1 | 0 | 0.9866 | 31.71 | 0 | 0.9478 | 0.02 | 0.8951 |
| **-0.10** | 44.72 | 0.08 | 0.7832 | 0.37 | 0.5433 | 31.71 | 0.05 | 0.8294 | 2.6 | 0.1068 |
| **-0.05** | 43.31 | 0 | 0.9512 | 0 | 1 | 28.05 | 0.73 | 0.3918 | 0 | 1 |
| **0.00** | 39.79 | 0 | 1 | 0.01 | 0.935 | 28.05 | --- | --- | --- | --- |
| **0.05** | 36.97 | 0 | 1 | 0 | 1 | 28.05 | --- | --- | --- | --- |
| **0.10** | 30.99 | 0 | 1 | 0 | 1 | 28.05 | --- | --- | --- | --- |
| **0.15** | 26.76 | 0 | 1 | 0 | 1 | 28.05 | --- | --- | --- | --- |
| **0.20** | 22.18 | 0 | 1 | 0 | 1 | 23.17 | --- | --- | --- | --- |
| **0.25** | 18.31 | 0.08 | 0.7782 | 0 | 1 | 20.73 | --- | --- | --- | --- |
| **0.30** | 16.2 | 0.63 | 0.4282 | 0.03 | 0.8668 | 18.29 | --- | --- | --- | --- |
| **0.35** | 14.08 | --- | --- | --- | --- | 15.85 | --- | --- | --- | --- |
| **0.40** | 10.56 | --- | --- | --- | --- | 14.63 | --- | --- | --- | --- |
| **0.45** | 8.8 | --- | --- | --- | --- | 10.98 | --- | --- | --- | --- |
| **0.50** | 9.15 | --- | --- | --- | --- | 9.76 | --- | --- | --- | --- |
| **0.55** | 8.45 | --- | --- | --- | --- | 9.76 | --- | --- | --- | --- |
| **0.60** | 7.75 | --- | --- | --- | --- | 9.76 | --- | --- | --- | --- |
| **0.65** | 7.39 | --- | --- | --- | --- | 7.32 | --- | --- | --- | --- |
| **0.70** | 5.28 | --- | --- | --- | --- | 7.32 | --- | --- | --- | --- |
| **0.75** | 3.17 | --- | --- | --- | --- | 6.1 | --- | --- | --- | --- |
| **0.80** | 1.06 | --- | --- | --- | --- | 2.44 | --- | --- | --- | --- |
| **0.85** | 1.06 | --- | --- | --- | --- | 2.44 | --- | --- | --- | --- |
| **0.90** | 1.06 | --- | --- | --- | --- | 2.44 | --- | --- | --- | --- |
| **0.95** | 0.7 | --- | --- | --- | --- | 2.44 | --- | --- | --- | --- |
| **1.00** | 0.7 | --- | --- | --- | --- | 1.22 | --- | --- | --- | --- |

*Bolded p values are those reaching statistical significance, p < 0.05. Grey rows represent the RAC range where cerebrovascular reactivity is generally considered to be intact.*

*ICP = intracranial pressure, iICP = individualized intracranial pressure thresholds, MAP = mean arterial pressure, RAC = correlation (R) between slow waves of AMP (A) and CPP (C), χ^2^ = chi-square value.*

Supplemental Appendix T. Chi-square outcome analysis of iICP derived using various PRx thresholds dichotomized by GCS-motor score

| **Threshold** | **GCS-Motor ≤ 3 (n = 114)** | | | | | **GCS-Motor > 3 (n = 128)** | | | | |
| --- | --- | --- | --- | --- | --- | --- | --- | --- | --- | --- |
|  | **Yield** | **Alive vs Dead** | | **Favorable vs Unfavorable** | | **Yield** | **Alive vs Dead** | | **Favorable vs Unfavorable** | |
|  |  | **χ^2^** | **p value** | **χ^2^** | **p value** |  | **χ^2^** | **p value** | **χ^2^** | **p value** |
| **-1.00** | 0 | --- | --- | --- | --- | 0 | --- | --- | --- | --- |
| **-0.95** | 0 | --- | --- | --- | --- | 0 | --- | --- | --- | --- |
| **-0.90** | 0 | --- | --- | --- | --- | 0 | --- | --- | --- | --- |
| **-0.85** | 0 | --- | --- | --- | --- | 0 | --- | --- | --- | --- |
| **-0.80** | 0 | --- | --- | --- | --- | 0 | --- | --- | --- | --- |
| **-0.75** | 0 | --- | --- | --- | --- | 0.4 | --- | --- | --- | --- |
| **-0.70** | 0 | --- | --- | --- | --- | 0.8 | --- | --- | --- | --- |
| **-0.65** | 0 | --- | --- | --- | --- | 0.8 | --- | --- | --- | --- |
| **-0.60** | 0.42 | --- | --- | --- | --- | 1.2 | --- | --- | --- | --- |
| **-0.55** | 0.84 | --- | --- | --- | --- | 1.2 | --- | --- | --- | --- |
| **-0.50** | 0.84 | --- | --- | --- | --- | 2.39 | --- | --- | --- | --- |
| **-0.45** | 1.69 | --- | --- | --- | --- | 2.79 | --- | --- | --- | --- |
| **-0.40** | 2.95 | --- | --- | --- | --- | 3.98 | --- | --- | --- | --- |
| **-0.35** | 2.95 | --- | --- | --- | --- | 4.78 | --- | --- | --- | --- |
| **-0.30** | 3.8 | --- | --- | --- | --- | 6.77 | --- | --- | --- | --- |
| **-0.25** | 5.49 | --- | --- | --- | --- | 8.37 | --- | --- | --- | --- |
| **-0.20** | 9.28 | --- | --- | --- | --- | 11.55 | --- | --- | --- | --- |
| **-0.15** | 12.24 | --- | --- | --- | --- | 13.55 | --- | --- | --- | --- |
| **-0.10** | 16.88 | 0.05 | 0.826 | 1.42 | 0.2335 | 15.94 | 1.09 | 0.2967 | 3.05 | 0.0808 |
| **-0.05** | 21.1 | 1.06 | 0.3024 | 2.12 | 0.1453 | 21.51 | 3.61 | 0.0575 | 6.33 | **0.0118** |
| **0.00** | 26.16 | 0 | 0.9753 | 0.43 | 0.5132 | 27.89 | 2.16 | 0.1415 | 5.05 | **0.0246** |
| **0.05** | 29.54 | 0.24 | 0.6276 | 0 | 1 | 31.08 | 1.44 | 0.2305 | 1.45 | 0.2287 |
| **0.10** | 29.96 | 0.2 | 0.6525 | 1.05 | 0.3065 | 32.27 | 0.37 | 0.5423 | 0.49 | 0.4849 |
| **0.15** | 32.49 | 3.23 | 0.0721 | 0.65 | 0.4215 | 33.86 | 2.66 | 0.1029 | 1.87 | 0.1716 |
| **0.20** | 32.91 | 1.82 | 0.1768 | 1.2 | 0.2732 | 36.65 | 3.28 | 0.0701 | 2.5 | 0.1137 |
| **0.25** | 34.6 | 0.17 | 0.6779 | 0.02 | 0.8848 | 35.06 | 0.87 | 0.3523 | 0.84 | 0.3583 |
| **0.30** | 32.91 | 0.14 | 0.704 | 0.27 | 0.6049 | 34.26 | 1.96 | 0.1612 | 2.45 | 0.1177 |
| **0.35** | 32.07 | 0 | 1 | 0 | 1 | 33.47 | 0.51 | 0.4739 | 0.22 | 0.6418 |
| **0.40** | 32.49 | 0 | 1 | 0 | 1 | 31.47 | 0.16 | 0.6883 | 0.07 | 0.7985 |
| **0.45** | 30.38 | 0.15 | 0.7007 | 0 | 1 | 28.69 | 1.44 | 0.2305 | 1.11 | 0.2913 |
| **0.50** | 29.11 | 0 | 1 | --- | --- | 27.09 | --- | --- | --- | --- |
| **0.55** | 27.85 | --- | --- | --- | --- | 26.69 | --- | --- | --- | --- |
| **0.60** | 27 | --- | --- | --- | --- | 23.11 | --- | --- | --- | --- |
| **0.65** | 25.74 | --- | --- | --- | --- | 21.51 | --- | --- | --- | --- |
| **0.70** | 24.05 | --- | --- | --- | --- | 18.73 | --- | --- | --- | --- |
| **0.75** | 22.36 | --- | --- | --- | --- | 15.54 | --- | --- | --- | --- |
| **0.80** | 18.14 | --- | --- | --- | --- | 15.14 | --- | --- | --- | --- |
| **0.85** | 15.19 | --- | --- | --- | --- | 11.95 | --- | --- | --- | --- |
| **0.90** | 11.39 | --- | --- | --- | --- | 9.16 | --- | --- | --- | --- |
| **0.95** | 8.02 | --- | --- | --- | --- | 8.76 | --- | --- | --- | --- |
| **1.00** | 6.33 | --- | --- | --- | --- | 7.57 | --- | --- | --- | --- |

*Bolded p values are those reaching statistical significance, p < 0.05. Grey rows represent the PRx range where cerebrovascular reactivity is generally considered to be intact.*

*GCS = Glasgow Coma Scale, ICP = intracranial pressure, iICP = individualized intracranial pressure thresholds, MAP = mean arterial pressure, PRx = pressure reactivity index (correlation between ICP and MAP), χ^2^ = chi-square value.*

Supplemental Appendix U. Chi-square outcome analysis of iICP derived using various PAx thresholds dichotomized by GCS-motor score

| **Threshold** | **GCS-Motor ≤ 3 (n = 114)** | | | | | **GCS-Motor > 3 (n = 128)** | | | | |
| --- | --- | --- | --- | --- | --- | --- | --- | --- | --- | --- |
|  | **Yield** | **Alive vs Dead** | | **Favorable vs Unfavorable** | | **Yield** | **Alive vs Dead** | | **Favorable vs Unfavorable** | |
|  |  | **χ^2^** | **p value** | **χ^2^** | **p value** |  | **χ^2^** | **p value** | **χ^2^** | **p value** |
| **-1.00** | 0 | --- | --- | --- | --- | 0 | --- | --- | --- | --- |
| **-0.95** | 0 | --- | --- | --- | --- | 0 | --- | --- | --- | --- |
| **-0.90** | 0 | --- | --- | --- | --- | 0 | --- | --- | --- | --- |
| **-0.85** | 0 | --- | --- | --- | --- | 0 | --- | --- | --- | --- |
| **-0.80** | 0 | --- | --- | --- | --- | 0 | --- | --- | --- | --- |
| **-0.75** | 0 | --- | --- | --- | --- | 0 | --- | --- | --- | --- |
| **-0.70** | 0 | --- | --- | --- | --- | 0 | --- | --- | --- | --- |
| **-0.65** | 0.42 | --- | --- | --- | --- | 0 | --- | --- | --- | --- |
| **-0.60** | 0.42 | --- | --- | --- | --- | 0.4 | --- | --- | --- | --- |
| **-0.55** | 1.27 | --- | --- | --- | --- | 0.8 | --- | --- | --- | --- |
| **-0.50** | 2.53 | --- | --- | --- | --- | 2.39 | --- | --- | --- | --- |
| **-0.45** | 4.64 | --- | --- | --- | --- | 4.38 | --- | --- | --- | --- |
| **-0.40** | 5.06 | --- | --- | --- | --- | 5.18 | --- | --- | --- | --- |
| **-0.35** | 8.02 | --- | --- | --- | --- | 6.77 | --- | --- | --- | --- |
| **-0.30** | 10.55 | --- | --- | --- | --- | 8.76 | --- | --- | --- | --- |
| **-0.25** | 15.19 | 0.01 | 0.9238 | 0.06 | 0.8063 | 12.35 | --- | --- | --- | --- |
| **-0.20** | 19.83 | 0.15 | 0.7011 | 0 | 1 | 15.14 | 1.81 | 0.1787 | 5.01 | **0.0252** |
| **-0.15** | 22.36 | 0.26 | 0.6126 | 2.12 | 0.1453 | 21.91 | 2.2 | 0.1376 | 6.84 | **0.0089** |
| **-0.10** | 26.16 | --- | --- | 5.86 | **0.0155** | 23.9 | 1.02 | 0.3125 | 3 | 0.0835 |
| **-0.05** | 27 | 0.87 | 0.3506 | 0.68 | 0.4103 | 29.08 | 0.44 | 0.5048 | 1.41 | 0.2351 |
| **0.00** | 27.43 | 0 | 1 | 0.37 | 0.5443 | 32.67 | 0.52 | 0.469 | 0.39 | 0.5335 |
| **0.05** | 27.43 | 0.14 | 0.7076 | 0 | 1 | 31.87 | 0.01 | 0.9415 | 0.02 | 0.8873 |
| **0.10** | 25.74 | 0 | 1 | 0 | 1 | 31.47 | 0.22 | 0.6358 | 0 | 0.9527 |
| **0.15** | 24.89 | 0 | 1 | 0.01 | 0.9434 | 30.28 | 0 | 1 | 0 | 1 |
| **0.20** | 24.47 | 0 | 1 | 0 | 1 | 29.08 | 0.15 | 0.6953 | 0.54 | 0.4609 |
| **0.25** | 23.63 | 0 | 0.9736 | 0.1 | 0.7562 | 26.29 | 0.45 | 0.5036 | 0.12 | 0.7245 |
| **0.30** | 22.78 | 0.92 | 0.3386 | 0.87 | 0.3518 | 23.51 | 0.19 | 0.6634 | 0.03 | 0.8545 |
| **0.35** | 21.1 | 1.73 | 0.1887 | 1.15 | 0.2842 | 20.72 | 0.19 | 0.6591 | 0.02 | 0.8776 |
| **0.40** | 17.3 | --- | --- | --- | --- | 18.73 | 0.77 | 0.3796 | 0.41 | 0.5222 |
| **0.45** | 12.24 | --- | --- | --- | --- | 16.73 | --- | --- | --- | --- |
| **0.50** | 11.81 | --- | --- | --- | --- | 14.74 | --- | --- | --- | --- |
| **0.55** | 10.55 | --- | --- | --- | --- | 11.95 | --- | --- | --- | --- |
| **0.60** | 10.13 | --- | --- | --- | --- | 10.76 | --- | --- | --- | --- |
| **0.65** | 7.59 | --- | --- | --- | --- | 8.76 | --- | --- | --- | --- |
| **0.70** | 6.33 | --- | --- | --- | --- | 6.77 | --- | --- | --- | --- |
| **0.75** | 5.06 | --- | --- | --- | --- | 4.78 | --- | --- | --- | --- |
| **0.80** | 3.38 | --- | --- | --- | --- | 3.59 | --- | --- | --- | --- |
| **0.85** | 2.95 | --- | --- | --- | --- | 3.19 | --- | --- | --- | --- |
| **0.90** | 2.11 | --- | --- | --- | --- | 2.39 | --- | --- | --- | --- |
| **0.95** | 1.69 | --- | --- | --- | --- | 1.59 | --- | --- | --- | --- |
| **1.00** | 1.27 | --- | --- | --- | --- | 1.59 | --- | --- | --- | --- |

*Bolded p values are those reaching statistical significance, p < 0.05. Grey rows represent the PAx range where cerebrovascular reactivity is generally considered to be intact.*

*GCS = Glasgow Coma Scale, ICP = intracranial pressure, iICP = individualized intracranial pressure thresholds, MAP = mean arterial pressure, PAx = pulse amplitude index (correlation between AMP and MAP), χ^2^ = chi-square value.*

Supplemental Appendix V. Chi-square outcome analysis of iICP derived using various RAC thresholds dichotomized by GCS-motor score

| **Threshold** | **GCS-Motor ≤ 3 (n = 114)** | | | | | **GCS-Motor > 3 (n = 128)** | | | | |
| --- | --- | --- | --- | --- | --- | --- | --- | --- | --- | --- |
|  | **Yield** | **Alive vs Dead** | | **Favorable vs Unfavorable** | | **Yield** | **Alive vs Dead** | | **Favorable vs Unfavorable** | |
|  |  | **χ^2^** | **p value** | **χ^2^** | **p value** |  | **χ^2^** | **p value** | **χ^2^** | **p value** |
| **-1.00** | 0 | --- | --- | --- | --- | 0.4 | --- | --- | --- | --- |
| **-0.95** | 0 | --- | --- | --- | --- | 0.4 | --- | --- | --- | --- |
| **-0.90** | 0 | --- | --- | --- | --- | 0.4 | --- | --- | --- | --- |
| **-0.85** | 0.42 | --- | --- | --- | --- | 0.4 | --- | --- | --- | --- |
| **-0.80** | 0.84 | --- | --- | --- | --- | 0.8 | --- | --- | --- | --- |
| **-0.75** | 1.69 | --- | --- | --- | --- | 1.99 | --- | --- | --- | --- |
| **-0.70** | 5.06 | --- | --- | --- | --- | 3.59 | --- | --- | --- | --- |
| **-0.65** | 8.02 | --- | --- | --- | --- | 5.18 | --- | --- | --- | --- |
| **-0.60** | 10.13 | --- | --- | --- | --- | 8.37 | --- | --- | --- | --- |
| **-0.55** | 13.08 | --- | --- | --- | --- | 10.36 | --- | --- | --- | --- |
| **-0.50** | 14.77 | --- | --- | --- | --- | 13.55 | --- | --- | --- | --- |
| **-0.45** | 16.88 | 0.37 | 0.5414 | 0.08 | 0.7767 | 15.94 | 3.67 | 0.0553 | 4.48 | **0.0343** |
| **-0.40** | 17.72 | 0 | 1 | 0 | 1 | 15.94 | 3.09 | 0.0787 | 1.7 | 0.1926 |
| **-0.35** | 21.1 | 0 | 1 | 0.78 | 0.377 | 18.33 | 4.83 | **0.028** | 3.38 | 0.0661 |
| **-0.30** | 21.94 | 0.26 | 0.6118 | 0 | 1 | 19.52 | 1.81 | 0.1782 | 1.03 | 0.3102 |
| **-0.25** | 21.52 | 0.51 | 0.4742 | 0.63 | 0.4282 | 21.12 | 2.36 | 0.1245 | 2.71 | 0.0998 |
| **-0.20** | 20.25 | 0 | 1 | 0 | 1 | 21.12 | 0.67 | 0.4119 | 1.09 | 0.2964 |
| **-0.15** | 18.57 | 0 | 1 | 0 | 1 | 22.31 | 0.03 | 0.8681 | 0.71 | 0.3997 |
| **-0.10** | 18.57 | 0.27 | 0.6051 | 1.39 | 0.2381 | 22.31 | 0.09 | 0.7645 | 0.3 | 0.5864 |
| **-0.05** | 17.3 | 0.06 | 0.8111 | 0.08 | 0.7727 | 23.11 | 0.01 | 0.9411 | 0 | 1 |
| **0.00** | 17.3 | 0 | 1 | 0.48 | 0.4898 | 20.32 | 0 | 1 | 0 | 0.9793 |
| **0.05** | 16.03 | 0.22 | 0.6391 | 0 | 0.9598 | 18.33 | 0 | 0.9812 | 0.81 | 0.3676 |
| **0.10** | 13.92 | --- | --- | --- | --- | 15.14 | 0.36 | 0.5481 | 0.15 | 0.6975 |
| **0.15** | 13.5 | --- | --- | --- | --- | 12.75 | --- | --- | --- | --- |
| **0.20** | 10.97 | --- | --- | --- | --- | 10.76 | --- | --- | --- | --- |
| **0.25** | 7.59 | --- | --- | --- | --- | 9.96 | --- | --- | --- | --- |
| **0.30** | 7.17 | --- | --- | --- | --- | 9.16 | --- | --- | --- | --- |
| **0.35** | 6.75 | --- | --- | --- | --- | 7.97 | --- | --- | --- | --- |
| **0.40** | 5.06 | --- | --- | --- | --- | 7.17 | --- | --- | --- | --- |
| **0.45** | 3.38 | --- | --- | --- | --- | 5.98 | --- | --- | --- | --- |
| **0.50** | 3.38 | --- | --- | --- | --- | 5.98 | --- | --- | --- | --- |
| **0.55** | 2.95 | --- | --- | --- | --- | 5.98 | --- | --- | --- | --- |
| **0.60** | 2.95 | --- | --- | --- | --- | 5.58 | --- | --- | --- | --- |
| **0.65** | 2.95 | --- | --- | --- | --- | 4.38 | --- | --- | --- | --- |
| **0.70** | 2.53 | --- | --- | --- | --- | 2.39 | --- | --- | --- | --- |
| **0.75** | 0.84 | --- | --- | --- | --- | 2.39 | --- | --- | --- | --- |
| **0.80** | 0.42 | --- | --- | --- | --- | 0 | --- | --- | --- | --- |
| **0.85** | 0.42 | --- | --- | --- | --- | 0 | --- | --- | --- | --- |
| **0.90** | 0.42 | --- | --- | --- | --- | 0 | --- | --- | --- | --- |
| **0.95** | 0.42 | --- | --- | --- | --- | 0 | --- | --- | --- | --- |
| **1.00** | 0.42 | --- | --- | --- | --- | 0 | --- | --- | --- | --- |

*Bolded p values are those reaching statistical significance, p < 0.05. Grey rows represent the RAC range where cerebrovascular reactivity is generally considered to be intact.*

*GCS = Glasgow Coma Scale, ICP = intracranial pressure, iICP = individualized intracranial pressure thresholds, MAP = mean arterial pressure, RAC = correlation (R) between slow waves of AMP (A) and CPP (C), χ^2^ = chi-square value.*

Supplemental Appendix W. Chi-square outcome analysis of iICP derived using various PRx thresholds dichotomized by Marshall CT score

| **Threshold** | **Marshall CT score ≤ 3 (n = 199)** | | | | | **Marshall CT score > 3 (n = 151)** | | | | |
| --- | --- | --- | --- | --- | --- | --- | --- | --- | --- | --- |
|  | **Yield** | **Alive vs Dead** | | **Favorable vs Unfavorable** | | **Yield** | **Alive vs Dead** | | **Favorable vs Unfavorable** | |
|  |  | **χ^2^** | **p value** | **χ^2^** | **p value** |  | **χ^2^** | **p value** | **χ^2^** | **p value** |
| **-1.00** | 0.47 | --- | --- | --- | --- | 0 | --- | --- | --- | --- |
| **-0.95** | 0.47 | --- | --- | --- | --- | 0 | --- | --- | --- | --- |
| **-0.90** | 0.47 | --- | --- | --- | --- | 0 | --- | --- | --- | --- |
| **-0.85** | 0.47 | --- | --- | --- | --- | 0 | --- | --- | --- | --- |
| **-0.80** | 0.47 | --- | --- | --- | --- | 0 | --- | --- | --- | --- |
| **-0.75** | 0.93 | --- | --- | --- | --- | 0 | --- | --- | --- | --- |
| **-0.70** | 1.87 | --- | --- | --- | --- | 0 | --- | --- | --- | --- |
| **-0.65** | 2.34 | --- | --- | --- | --- | 0 | --- | --- | --- | --- |
| **-0.60** | 3.27 | --- | --- | --- | --- | 0.6 | --- | --- | --- | --- |
| **-0.55** | 3.27 | --- | --- | --- | --- | 0.6 | --- | --- | --- | --- |
| **-0.50** | 4.67 | --- | --- | --- | --- | 1.2 | --- | --- | --- | --- |
| **-0.45** | 5.61 | --- | --- | --- | --- | 2.41 | --- | --- | --- | --- |
| **-0.40** | 6.54 | --- | --- | --- | --- | 4.82 | --- | --- | --- | --- |
| **-0.35** | 7.48 | --- | --- | --- | --- | 5.42 | --- | --- | --- | --- |
| **-0.30** | 9.35 | --- | --- | --- | --- | 8.43 | --- | --- | --- | --- |
| **-0.25** | 12.62 | --- | --- | --- | --- | 10.84 | --- | --- | --- | --- |
| **-0.20** | 18.69 | 0 | 0.9716 | 0 | 1 | 15.06 | 0.18 | 0.6694 | 0.44 | 0.5057 |
| **-0.15** | 25.23 | 0.15 | 0.7013 | 0.13 | 0.723 | 18.07 | 0 | 1 | 0 | 1 |
| **-0.10** | 35.51 | 0.01 | 0.9097 | 0.56 | 0.4535 | 24.1 | 0.42 | 0.5188 | 0.08 | 0.7778 |
| **-0.05** | 41.59 | 0.01 | 0.9229 | 1.9 | 0.1682 | 36.14 | 5.53 | 0.0187 | 2.65 | 0.1036 |
| **0.00** | 50.93 | 0.12 | 0.7243 | 0.23 | 0.6293 | 46.99 | 2.83 | 0.0925 | 1.83 | 0.1761 |
| **0.05** | 60.28 | 1.85 | 0.1736 | 7.84 | **0.0051** | 51.81 | 2.97 | 0.0848 | 0.6 | 0.4372 |
| **0.10** | 65.89 | 0.79 | 0.3749 | 4.84 | **0.0278** | 54.22 | 1.26 | 0.2609 | 1.97 | 0.1604 |
| **0.15** | 70.09 | 0.4 | 0.5249 | 1.09 | 0.2968 | 59.04 | 2.46 | 0.1164 | 2.04 | 0.1531 |
| **0.20** | 71.96 | 1.13 | 0.2887 | 4.68 | **0.0305** | 61.45 | 2.11 | 0.1462 | 1.09 | 0.2964 |
| **0.25** | 73.36 | 1.06 | 0.303 | 3.64 | 0.0563 | 62.65 | 1.68 | 0.1953 | 1.02 | 0.3126 |
| **0.30** | 70.56 | 0 | 1 | 0.95 | 0.3288 | 62.05 | 1.38 | 0.2398 | 0.31 | 0.576 |
| **0.35** | 67.76 | 0.09 | 0.7617 | 0 | 1 | 63.25 | 1.73 | 0.1886 | 0.47 | 0.4931 |
| **0.40** | 62.62 | 0 | 1 | 0 | 1 | 63.25 | 0.09 | 0.7643 | 0.09 | 0.7642 |
| **0.45** | 58.41 | 0.14 | 0.706 | 0.15 | 0.6939 | 60.24 | 0 | 1 | 0 | 1 |
| **0.50** | 56.07 | 1.02 | 0.3132 | --- | --- | 54.22 | 0 | 1 | 0 | 0.9556 |
| **0.55** | 51.87 | --- | --- | --- | --- | 53.61 | 0 | 1 | 0.01 | 0.9403 |
| **0.60** | 45.79 | --- | --- | --- | --- | 47.59 | 0.24 | 0.6266 | 0.01 | 0.9165 |
| **0.65** | 41.59 | --- | --- | --- | --- | 44.58 | 0.54 | 0.4642 | 0.17 | 0.6828 |
| **0.70** | 36.92 | --- | --- | --- | --- | 42.17 | 0.07 | 0.7889 | 0 | 1 |
| **0.75** | 31.78 | --- | --- | --- | --- | 39.76 | 0.32 | 0.5727 | 0.07 | 0.7892 |
| **0.80** | 28.5 | --- | --- | --- | --- | 36.75 | --- | --- | --- | --- |
| **0.85** | 25.23 | --- | --- | --- | --- | 29.52 | --- | --- | --- | --- |
| **0.90** | 20.56 | --- | --- | --- | --- | 21.69 | --- | --- | --- | --- |
| **0.95** | 15.89 | --- | --- | --- | --- | 16.87 | --- | --- | --- | --- |
| **1.00** | 11.68 | --- | --- | --- | --- | 13.86 | --- | --- | --- | --- |

*Bolded p values are those reaching statistical significance, p < 0.05. Grey rows represent the PRx range where cerebrovascular reactivity is generally considered to be intact.*

*CT = computerized tomography, ICP = intracranial pressure, iICP = individualized intracranial pressure thresholds, MAP = mean arterial pressure, PRx = pressure reactivity index (correlation between ICP and MAP), χ^2^ = chi-square value.*

Supplemental Appendix X. Chi-square outcome analysis of iICP derived using various PAx thresholds dichotomized by Marshall CT score

| **Threshold** | **Marshall CT score ≤ 3 (n = 199)** | | | | | **Marshall CT score > 3 (n = 151)** | | | | |
| --- | --- | --- | --- | --- | --- | --- | --- | --- | --- | --- |
|  | **Yield** | **Alive vs Dead** | | **Favorable vs Unfavorable** | | **Yield** | **Alive vs Dead** | | **Favorable vs Unfavorable** | |
|  |  | **χ^2^** | **p value** | **χ^2^** | **p value** |  | **χ^2^** | **p value** | **χ^2^** | **p value** |
| **-1.00** | 0 | --- | --- | --- | --- | 0 | --- | --- | --- | --- |
| **-0.95** | 0 | --- | --- | --- | --- | 0 | --- | --- | --- | --- |
| **-0.90** | 0 | --- | --- | --- | --- | 0 | --- | --- | --- | --- |
| **-0.85** | 0 | --- | --- | --- | --- | 0 | --- | --- | --- | --- |
| **-0.80** | 0 | --- | --- | --- | --- | 0.6 | --- | --- | --- | --- |
| **-0.75** | 0.47 | --- | --- | --- | --- | 0.6 | --- | --- | --- | --- |
| **-0.70** | 0.47 | --- | --- | --- | --- | 0.6 | --- | --- | --- | --- |
| **-0.65** | 0.47 | --- | --- | --- | --- | 1.2 | --- | --- | --- | --- |
| **-0.60** | 0.93 | --- | --- | --- | --- | 1.2 | --- | --- | --- | --- |
| **-0.55** | 2.34 | --- | --- | --- | --- | 1.2 | --- | --- | --- | --- |
| **-0.50** | 3.74 | --- | --- | --- | --- | 4.22 | --- | --- | --- | --- |
| **-0.45** | 7.94 | --- | --- | --- | --- | 6.02 | --- | --- | --- | --- |
| **-0.40** | 9.35 | --- | --- | --- | --- | 7.83 | --- | --- | --- | --- |
| **-0.35** | 14.02 | --- | --- | --- | --- | 10.84 | --- | --- | --- | --- |
| **-0.30** | 20.56 | 0.52 | 0.4711 | 0 | 1 | 13.86 | --- | --- | --- | --- |
| **-0.25** | 25.7 | 0.86 | 0.3524 | 0.26 | 0.612 | 21.08 | 0.72 | 0.3972 | 0.78 | 0.3783 |
| **-0.20** | 32.71 | 5.59 | **0.0181** | 5.13 | **0.0235** | 30.72 | 1.69 | 0.1938 | 2.47 | 0.1163 |
| **-0.15** | 42.99 | 0.44 | 0.5091 | 0.85 | 0.3575 | 39.76 | 0.99 | 0.3191 | 0.92 | 0.3384 |
| **-0.10** | 53.27 | 0.08 | 0.7758 | 2.68 | 0.1015 | 45.18 | 0.56 | 0.4559 | 0.22 | 0.642 |
| **-0.05** | 60.75 | 0 | 0.955 | 0.8 | 0.3714 | 52.41 | 0.36 | 0.5498 | 0.41 | 0.5237 |
| **0.00** | 69.63 | 0.23 | 0.6307 | 1.77 | 0.1835 | 54.22 | 1.31 | 0.2517 | 0.18 | 0.6728 |
| **0.05** | 68.22 | 0.19 | 0.659 | 1.77 | 0.1838 | 58.43 | 1.35 | 0.2456 | 0.19 | 0.6648 |
| **0.10** | 64.02 | 0 | 0.9549 | 0.13 | 0.7186 | 56.63 | 4.14 | **0.0418** | 1.11 | 0.2918 |
| **0.15** | 62.62 | 0.01 | 0.9101 | 0.19 | 0.6628 | 52.41 | 5.74 | **0.0165** | 2.57 | 0.1087 |
| **0.20** | 58.88 | 0.79 | 0.3755 | 1.41 | 0.2344 | 50.6 | 0.45 | 0.5002 | 0.01 | 0.9403 |
| **0.25** | 53.27 | 0.72 | 0.3948 | 0.98 | 0.3218 | 50 | 1.06 | 0.3024 | 0.15 | 0.6957 |
| **0.30** | 48.13 | 0 | 1 | 0.46 | 0.4968 | 48.19 | 1.77 | 0.1833 | 0.51 | 0.4766 |
| **0.35** | 42.99 | 0 | 1 | 0 | 1 | 42.77 | 2.93 | 0.0869 | 1.27 | 0.2604 |
| **0.40** | 36.92 | 0 | 0.982 | 0 | 1 | 36.14 | 1.08 | 0.2993 | 0.18 | 0.6749 |
| **0.45** | 33.64 | --- | --- | --- | --- | 29.52 | 1.66 | 0.1977 | 0.9 | 0.3419 |
| **0.50** | 29.91 | --- | --- | --- | --- | 26.51 | 0.46 | 0.4968 | 0.22 | 0.6427 |
| **0.55** | 25.7 | --- | --- | --- | --- | 21.69 | --- | --- | --- | --- |
| **0.60** | 20.56 | --- | --- | --- | --- | 21.08 | --- | --- | --- | --- |
| **0.65** | 17.76 | --- | --- | --- | --- | 16.27 | --- | --- | --- | --- |
| **0.70** | 13.55 | --- | --- | --- | --- | 14.46 | --- | --- | --- | --- |
| **0.75** | 11.68 | --- | --- | --- | --- | 10.24 | --- | --- | --- | --- |
| **0.80** | 9.81 | --- | --- | --- | --- | 7.23 | --- | --- | --- | --- |
| **0.85** | 7.94 | --- | --- | --- | --- | 6.63 | --- | --- | --- | --- |
| **0.90** | 7.01 | --- | --- | --- | --- | 4.22 | --- | --- | --- | --- |
| **0.95** | 5.14 | --- | --- | --- | --- | 3.01 | --- | --- | --- | --- |
| **1.00** | 2.8 | --- | --- | --- | --- | 3.01 | --- | --- | --- | --- |

*Bolded p values are those reaching statistical significance, p < 0.05. Grey rows represent the PAx range where cerebrovascular reactivity is generally considered to be intact.*

*CT = computerized tomography, ICP = intracranial pressure, iICP = individualized intracranial pressure thresholds, MAP = mean arterial pressure, PAx = pulse amplitude index (correlation between AMP and MAP), χ^2^ = chi-square value.*

Supplemental Appendix Y. Chi-square outcome analysis of iICP derived using various RAC thresholds dichotomized by Marshall CT score

| **Threshold** | **Marshall CT score ≤ 3 (n = 199)** | | | | | **Marshall CT score > 3 (n = 151)** | | | | |
| --- | --- | --- | --- | --- | --- | --- | --- | --- | --- | --- |
|  | **Yield** | **Alive vs Dead** | | **Favorable vs Unfavorable** | | **Yield** | **Alive vs Dead** | | **Favorable vs Unfavorable** | |
|  |  | **χ^2^** | **p value** | **χ^2^** | **p value** |  | **χ^2^** | **p value** | **χ^2^** | **p value** |
| **-1.00** | 0.93 | --- | --- | --- | --- | 0 | --- | --- | --- | --- |
| **-0.95** | 1.4 | --- | --- | --- | --- | 0 | --- | --- | --- | --- |
| **-0.90** | 1.4 | --- | --- | --- | --- | 0 | --- | --- | --- | --- |
| **-0.85** | 1.4 | --- | --- | --- | --- | 0.6 | --- | --- | --- | --- |
| **-0.80** | 3.74 | --- | --- | --- | --- | 1.2 | --- | --- | --- | --- |
| **-0.75** | 9.35 | --- | --- | --- | --- | 1.2 | --- | --- | --- | --- |
| **-0.70** | 14.49 | --- | --- | --- | --- | 1.81 | --- | --- | --- | --- |
| **-0.65** | 20.09 | 0.52 | 0.4727 | 0 | 1 | 4.22 | --- | --- | --- | --- |
| **-0.60** | 28.04 | 0.53 | 0.4669 | 0 | 0.9653 | 7.83 | --- | --- | --- | --- |
| **-0.55** | 33.18 | 0.01 | 0.9378 | 0.08 | 0.7714 | 11.45 | --- | --- | --- | --- |
| **-0.50** | 35.98 | 1.79 | 0.1809 | 0.13 | 0.7159 | 20.48 | 0.7 | 0.4031 | 0 | 1 |
| **-0.45** | 35.98 | 6.55 | **0.0105** | 2.88 | 0.0898 | 27.11 | 4.3 | **0.0381** | 0.84 | 0.3607 |
| **-0.40** | 35.98 | 4.52 | **0.0336** | 2.43 | 0.1188 | 32.53 | 0.89 | 0.3457 | 0 | 1 |
| **-0.35** | 41.12 | 1.48 | 0.2243 | 0.16 | 0.6905 | 36.75 | 0.12 | 0.724 | 0 | 0.9638 |
| **-0.30** | 42.06 | 0.87 | 0.3503 | 0 | 1 | 41.57 | 5.18 | **0.0229** | 1.85 | 0.1739 |
| **-0.25** | 40.19 | 0 | 1 | 0 | 1 | 44.58 | 8.71 | **0.0032** | 4.43 | **0.0353** |
| **-0.20** | 35.51 | 0 | 1 | 0.17 | 0.6788 | 46.39 | 1.11 | 0.2913 | 0.52 | 0.4707 |
| **-0.15** | 35.51 | 0.02 | 0.8847 | 0.37 | 0.5424 | 45.78 | 0 | 1 | 0 | 1 |
| **-0.10** | 32.71 | 0.08 | 0.7815 | 1.44 | 0.2302 | 48.8 | 0 | 1 | 0.29 | 0.5893 |
| **-0.05** | 30.84 | 0.01 | 0.9034 | 0.52 | 0.471 | 46.99 | 0 | 1 | 0.19 | 0.6643 |
| **0.00** | 27.57 | 0 | 0.9943 | 1.43 | 0.2323 | 45.18 | 0.03 | 0.8584 | 0 | 1 |
| **0.05** | 25.23 | 0 | 1 | 0.05 | 0.8305 | 42.77 | 0.03 | 0.8693 | 0 | 1 |
| **0.10** | 21.96 | 0 | 1 | 0 | 1 | 37.35 | 0.41 | 0.5219 | 0 | 0.9845 |
| **0.15** | 18.69 | 0 | 1 | 0 | 1 | 34.94 | 2.3 | 0.1291 | 0.67 | 0.4131 |
| **0.20** | 14.49 | --- | --- | --- | --- | 30.12 | 2.19 | 0.1391 | 0.65 | 0.4217 |
| **0.25** | 13.08 | --- | --- | --- | --- | 24.7 | 1.9 | 0.1675 | 0.69 | 0.4072 |
| **0.30** | 11.21 | --- | --- | --- | --- | 22.29 | 1.11 | 0.2911 | 0.55 | 0.4566 |
| **0.35** | 9.81 | --- | --- | --- | --- | 19.28 | 0.55 | 0.457 | 0.19 | 0.6624 |
| **0.40** | 8.88 | --- | --- | --- | --- | 13.86 | --- | --- | --- | --- |
| **0.45** | 7.01 | --- | --- | --- | --- | 11.45 | --- | --- | --- | --- |
| **0.50** | 6.54 | --- | --- | --- | --- | 12.05 | --- | --- | --- | --- |
| **0.55** | 6.54 | --- | --- | --- | --- | 10.84 | --- | --- | --- | --- |
| **0.60** | 6.07 | --- | --- | --- | --- | 10.24 | --- | --- | --- | --- |
| **0.65** | 5.61 | --- | --- | --- | --- | 9.04 | --- | --- | --- | --- |
| **0.70** | 4.21 | --- | --- | --- | --- | 7.23 | --- | --- | --- | --- |
| **0.75** | 2.8 | --- | --- | --- | --- | 4.82 | --- | --- | --- | --- |
| **0.80** | 0.93 | --- | --- | --- | --- | 1.81 | --- | --- | --- | --- |
| **0.85** | 0.93 | --- | --- | --- | --- | 1.81 | --- | --- | --- | --- |
| **0.90** | 0.93 | --- | --- | --- | --- | 1.81 | --- | --- | --- | --- |
| **0.95** | 0.47 | --- | --- | --- | --- | 1.81 | --- | --- | --- | --- |
| **1.00** | 0 | --- | --- | --- | --- | 1.81 | --- | --- | --- | --- |

*Bolded p values are those reaching statistical significance, p < 0.05. Grey rows represent the RAC range where cerebrovascular reactivity is generally considered to be intact.*

*CT = computerized tomography, ICP = intracranial pressure, iICP = individualized intracranial pressure thresholds, MAP = mean arterial pressure, RAC = correlation (R) between slow waves of AMP (A) and CPP (C), χ^2^ = chi-square value.*
